# Supplementary material for: Hypermetabolism in mice carrying a near complete human chromosome 21
Source: bioRxiv. 2023 Jan 31:2023.01.30.526183. Preprint. [Version 1] doi: 10.1101/2023.01.30.526183 (PMC9915508; doi:10.1101/2023.01.30.526183)
Supplement: Supplement 1 [file NIHPP2023.01.30.526183v1-supplement-1.pdf]

bioRxiv preprint doi: https://doi.org/10.1101/2023.01.30.526183; this version posted January 31, 2023. The copyright holder for this preprint (which was not certified by peer review) is the author/funder, who has granted bioRxiv a license to display the preprint in perpetuity. It is made available under aCC-BY 4.0 International license.

Fig. SI

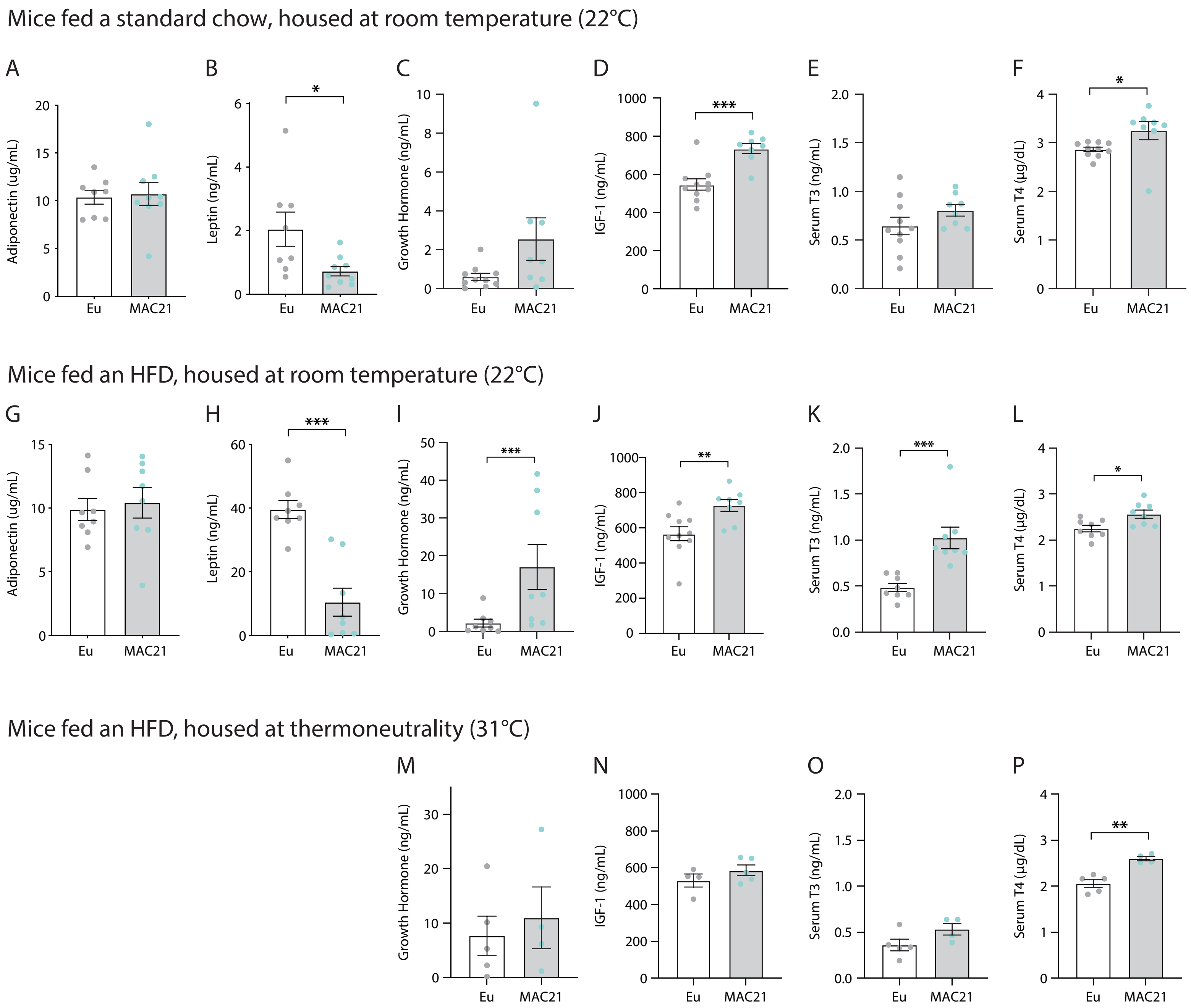

bioRxiv preprint doi: https://doi.org/10.1101/2023.01.30.526183; this version posted January 31, 2023. The copyright holder for this preprint (which was not certified by peer review) is the author/funder, who has granted bioRxiv a license to display the preprint in perpetuity. It is made available under aCC-BY 4.0 International license.

Figure S1. ELISA data from Euploid and MAC21 mice. Circulating levels of adiponectin (A), leptin (B), growth hormone (C), IGF-1 (D), thyroid hormone (T3, E), and thyroxine (T4, F) in chow-fed mice housed at room temperature (22°C). Circulating levels of adiponectin (G), leptin (H), growth hormone (I), IGF-1 (J), T3 (K), and T4 (L) in HFD-fed mice housed at 22°C. Circulating levels of growth hormone (M), IGF-1 (N), T3 (O), and T4 (P) in HFD-fed mice housed at thermoneutrality (30°C).

bioRxiv preprint doi: https://doi.org/10.1101/2023.01.30.526183; this version posted January 31, 2023. The copyright holder for this preprint (which was not certified by peer review) is the author/funder, who has granted bioRxiv a license to display the preprint in perpetuity. It is made available under aCC-BY 4.0 International license.

Fig. S2

Chow diet.

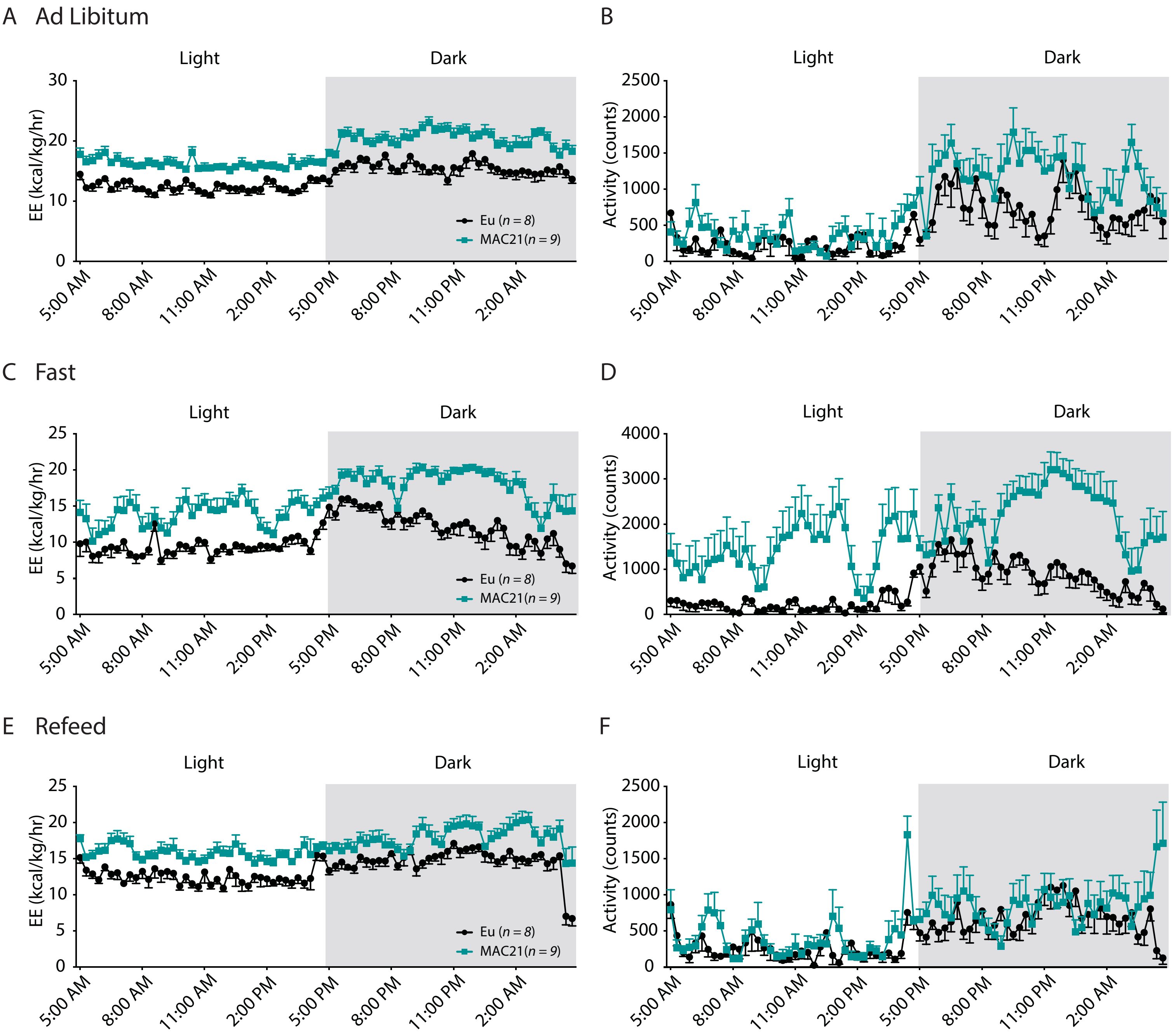

bioRxiv preprint doi: https://doi.org/10.1101/2023.01.30.526183; this version posted January 31, 2023. The copyright holder for this preprint (which was not certified by peer review) is the author/funder, who has granted bioRxiv a license to display the preprint in perpetuity. It is made available under aCC-BY 4.0 International license.

Figure S2. Indirect calorimetry of energy expenditure and physical activity in Euploid and MAC21 mice fed a standard chow. A-B) Energy expenditure (EE) and physical activity over a 24 h period in ad libitum chow-fed mice. **C-D)** Energy expenditure (EE) and physical activity over a 24 h period in fasted mice. **E-F)** Energy expenditure (EE) and physical activity over a 24 h period of refeeding after a fast.

bioRxiv preprint doi: https://doi.org/10.1101/2023.01.30.526183; this version posted January 31, 2023. The copyright holder for this preprint (which was not certified by peer review) is the author/funder, who has granted bioRxiv a license to display the preprint in perpetuity. It is made available under aCC-BY 4.0 International license.

Fig. S3

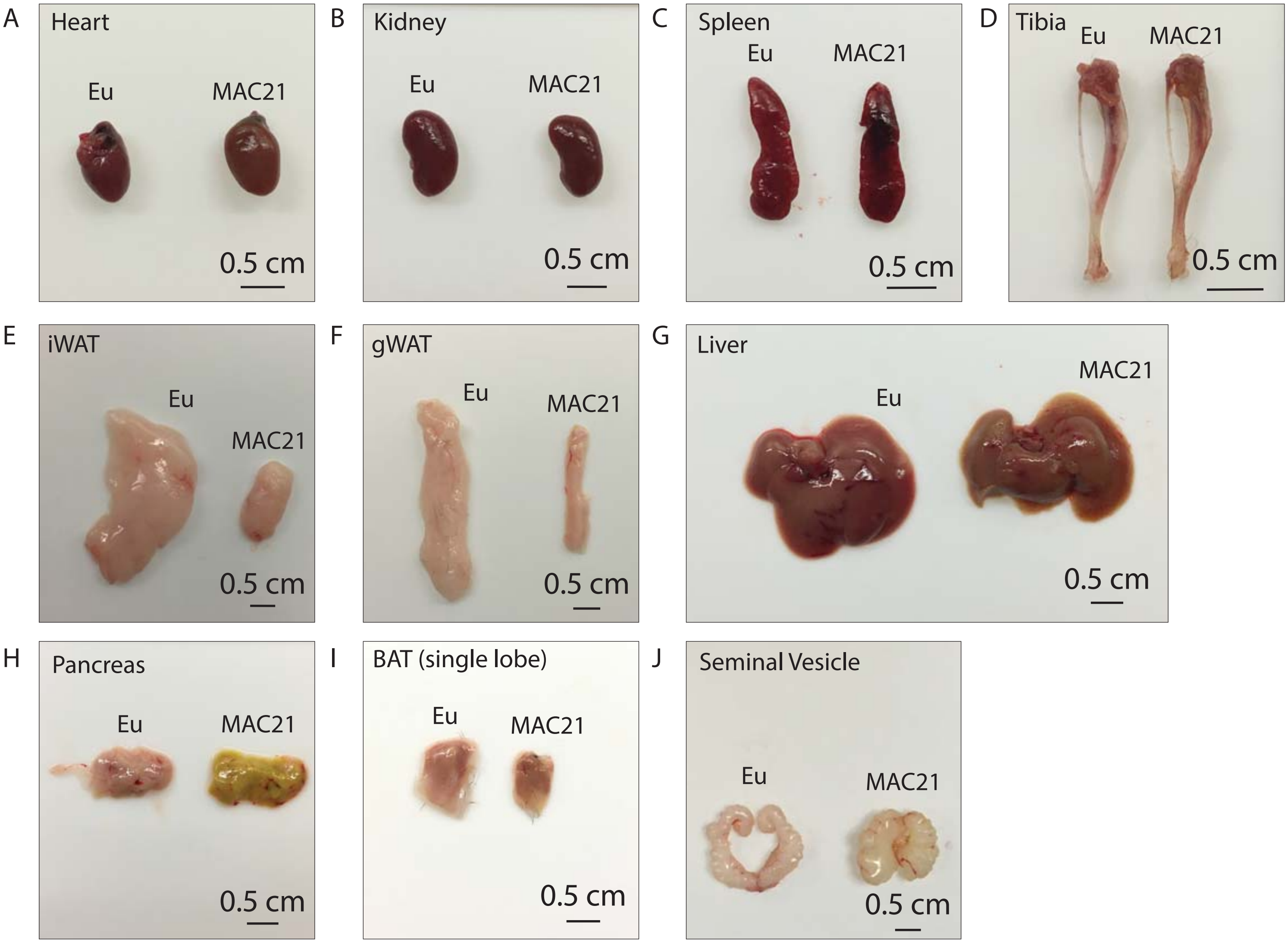

bioRxiv preprint doi: https://doi.org/10.1101/2023.01.30.526183; this version posted January 31, 2023. The copyright holder for this preprint (which was not certified by peer review) is the author/funder, who has granted bioRxiv a license to display the preprint in perpetuity. It is made available under aCC-BY 4.0 International license.

Figure S3. Representative tissue dissection images of Euploid and MAC21 mice fed a high-fat diet.

Representative side-by-side dissection images of **A)** heart, **B)** kidney, **C)** spleen, **D)** tibia, **E)** inguinal white adipose tissue (iWAT), **F)** gonadal white adipose tissue (gWAT), **G)** liver, **H)** pancreas, **I)** brown adipose tissue (BAT), and **J)** seminal vesicle. Mice were housed at ambient room temperature (22°C).

bioRxiv preprint doi: https://doi.org/10.1101/2023.01.30.526183; this version posted January 31, 2023. The copyright holder for this preprint (which was not certified by peer review) is the author/funder, who has granted bioRxiv a license to display the preprint in perpetuity. It is made available under aCC-BY 4.0 International license.

Fig. S4

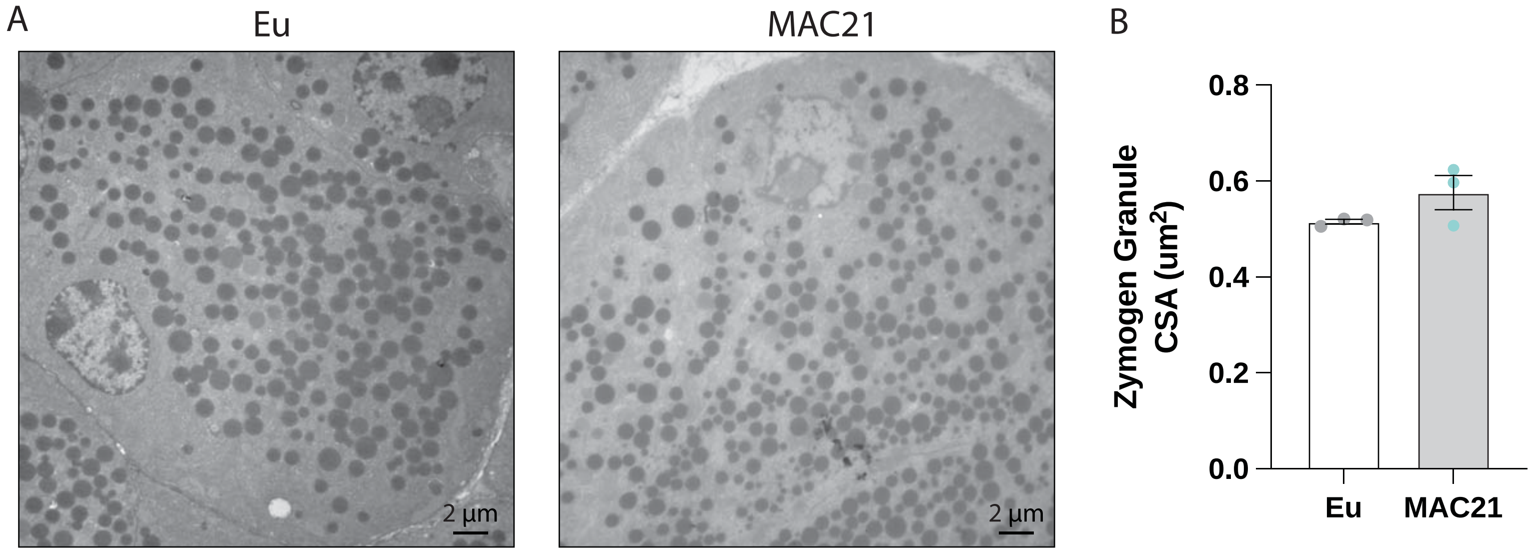

bioRxiv preprint doi: https://doi.org/10.1101/2023.01.30.526183; this version posted January 31, 2023. The copyright holder for this preprint (which was not certified by peer review) is the author/funder, who has granted bioRxiv a license to display the preprint in perpetuity. It is made available under aCC-BY 4.0 International license.

Figure S4. Electron micrograph quantification of pancreatic acinar cell zymogen granules. A and B) Representative Euploid and MAC21 acinar cells. **C)** Average zymogen granule cross-sectional area (CSA) quantification. Each data point represents a mouse average comprised of at least 3,600 zymogen granules from six unique locations within the pancreas. Analyses were performed on a total of 6 randomly selected HFD-fed mice (WT, *n* = 3; KO, *n* = 3).

bioRxiv preprint doi: https://doi.org/10.1101/2023.01.30.526183; this version posted January 31, 2023. The copyright holder for this preprint (which was not certified by peer review) is the author/funder, who has granted bioRxiv a license to display the preprint in perpetuity. It is made available under aCC-BY 4.0 International license.

Fig. S5

Fecal energy data from HFD-fed mice housed at 22°C

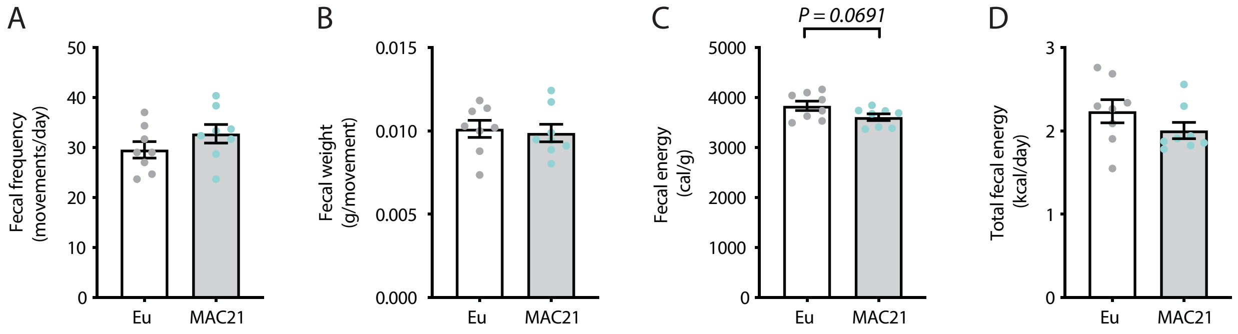

Fecal data from HFD-fed mice housed at thermoneutrality (30°C)

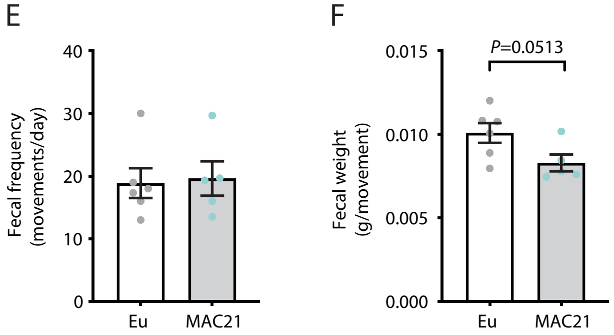

bioRxiv preprint doi: https://doi.org/10.1101/2023.01.30.526183; this version posted January 31, 2023. The copyright holder for this preprint (which was not certified by peer review) is the author/funder, who has granted bioRxiv a license to display the preprint in perpetuity. It is made available under aCC-BY 4.0 International license.

Figure S5. Fecal data from Euploid and MAC21 mice fed a high-fat diet at room temperature (22°C) or thermoneutrality (30°C). Fecal energy data from HFD-fed mice housed at room temperature (22°C) showing **A)** rate of fecal pellet production (bowel movement), **B)** Average fecal pellet weight, and **C-D)** Fecal energy composition as measure by fecal bomb calorimetry. Fecal data from mice fed a high-fat diet housed at thermoneutrality showing **E)** rate of fecal pellet production and **F)** average fecal weight per pellet.

bioRxiv preprint doi: https://doi.org/10.1101/2023.01.30.526183; this version posted January 31, 2023. The copyright holder for this preprint (which was not certified by peer review) is the author/funder, who has granted bioRxiv a license to display the preprint in perpetuity. It is made available under aCC-BY 4.0 International license.

Fig. S6

High-fat diet

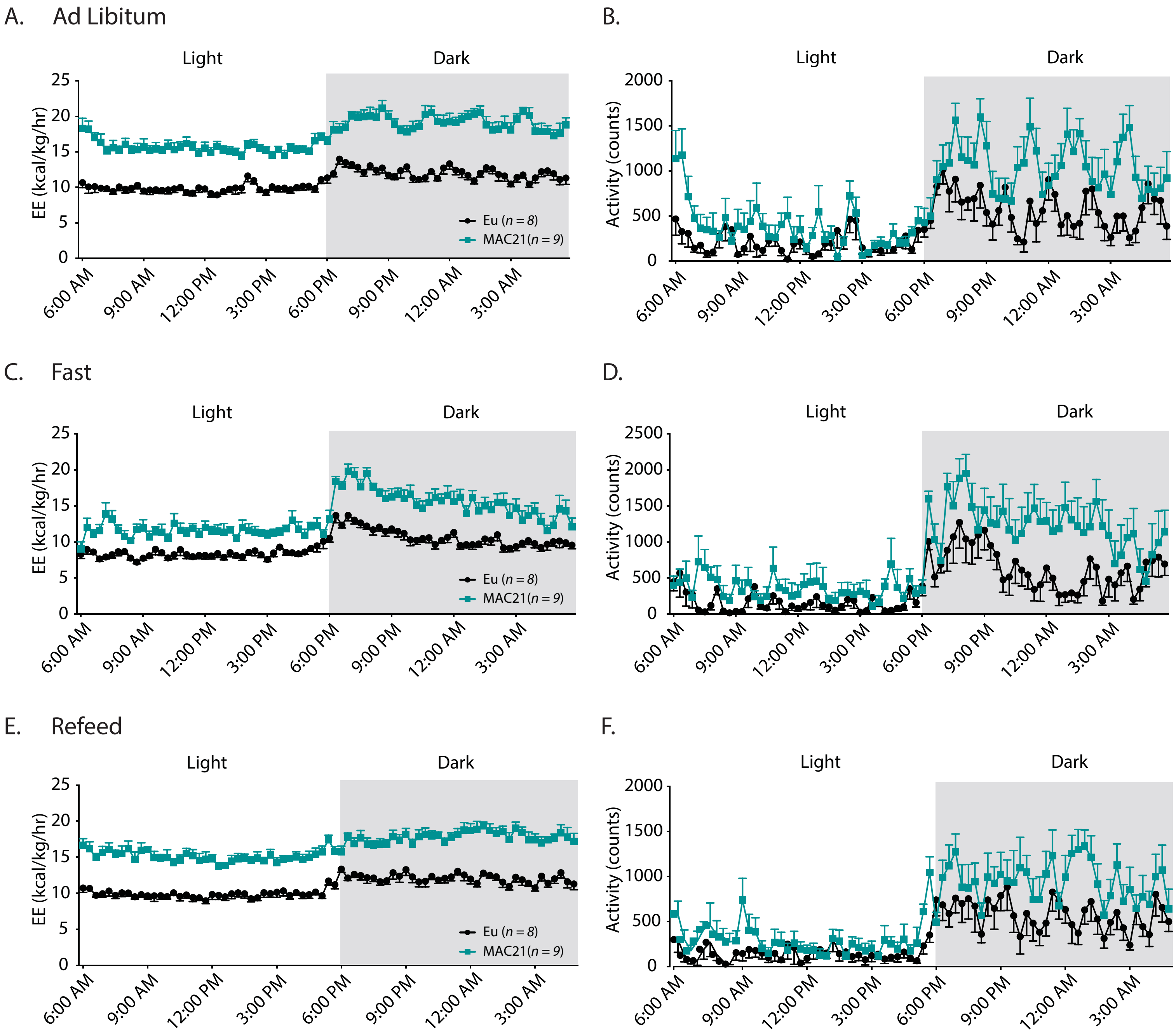

bioRxiv preprint doi: https://doi.org/10.1101/2023.01.30.526183; this version posted January 31, 2023. The copyright holder for this preprint (which was not certified by peer review) is the author/funder, who has granted bioRxiv a license to display the preprint in perpetuity. It is made available under aCC-BY 4.0 International license.

Figure S6. Indirect calorimetry of energy expenditure and physical activity in Euploid and MAC21 mice fed a high-fat diet. A-B) Energy expenditure (EE) and physical activity over a 24 h period in ad libitum HFD-fed mice. **C-D)** Energy expenditure (EE) and physical activity over a 24 h period in fasted mice. **E-F)** Energy expenditure (EE) and physical activity over a 24 h period of refeeding after a fast.

bioRxiv preprint doi: https://doi.org/10.1101/2023.01.30.526183; this version posted January 31, 2023. The copyright holder for this preprint (which was not certified by peer review) is the author/funder, who has granted bioRxiv a license to display the preprint in perpetuity. It is made available under aCC-BY 4.0 International license.

Fig. S7

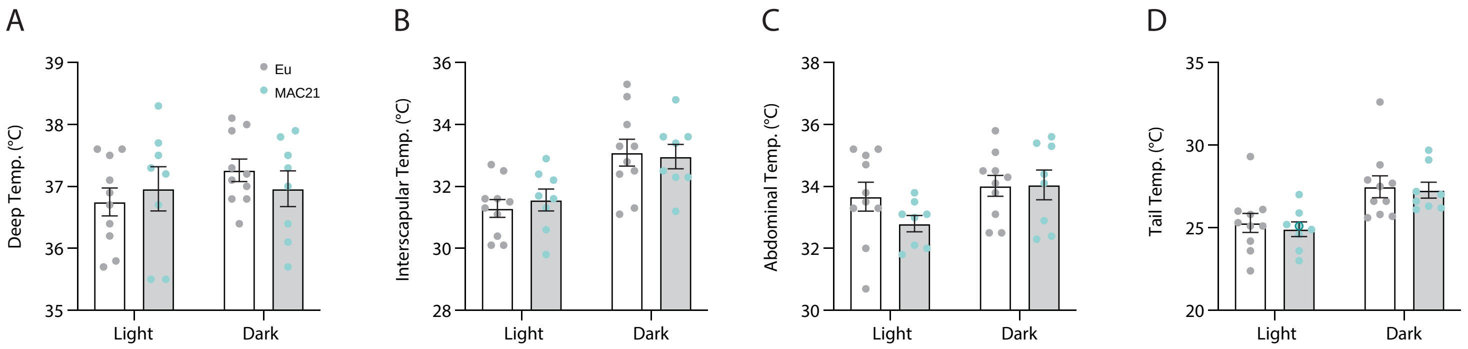

bioRxiv preprint doi: https://doi.org/10.1101/2023.01.30.526183; this version posted January 31, 2023. The copyright holder for this preprint (which was not certified by peer review) is the author/funder, who has granted bioRxiv a license to display the preprint in perpetuity. It is made available under aCC-BY 4.0 International license.

Figure S7. Light and dark cycle body temperature of Euploid and MAC21 mice fed a standard chow. A) Deep colonic temperature. **B)** Interscapular skin temperature. **C)** Abdominal Skin temperature. **D)** Tail skin temperature. Each data point represents the average of three days of independent data collection.

bioRxiv preprint doi: https://doi.org/10.1101/2023.01.30.526183; this version posted January 31, 2023. The copyright holder for this preprint (which was not certified by peer review) is the author/funder, who has granted bioRxiv a license to display the preprint in perpetuity. It is made available under aCC-BY 4.0 International license.

Fig. S8

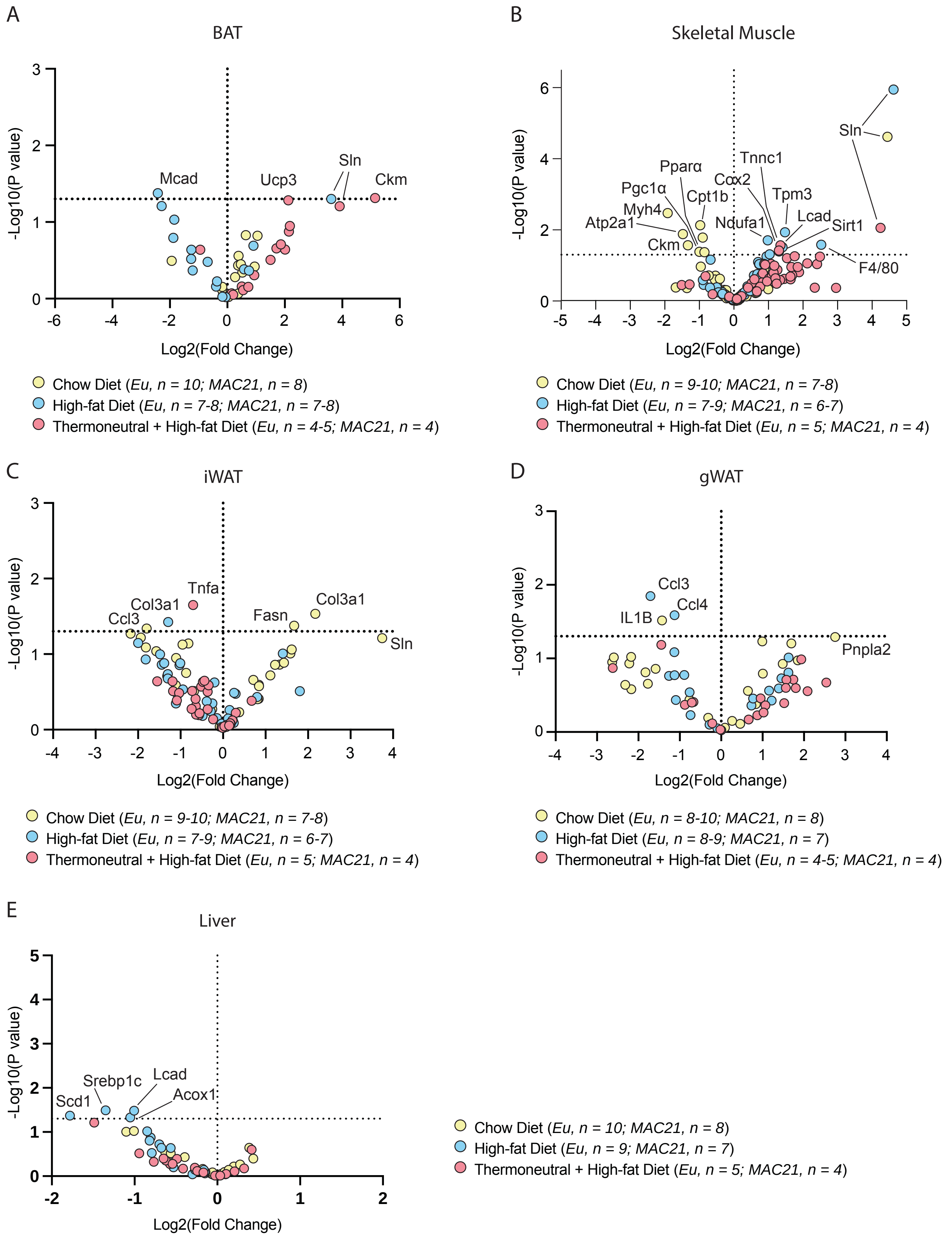

bioRxiv preprint doi: https://doi.org/10.1101/2023.01.30.526183; this version posted January 31, 2023. The copyright holder for this preprint (which was not certified by peer review) is the author/funder, who has granted bioRxiv a license to display the preprint in perpetuity. It is made available under aCC-BY 4.0 International license.

Figure S8. Volcano plot of all qPCR results from BAT, skeletal muscle, and white adipose tissue.

Overlaid results of Euploid and MAC21 mice across all conditions assayed (chow diet at 22°C, high-fat diet at 22°C, and high-fat diet at 30°C) for **A)** Brown adipose tissue (BAT), **B)** Gastrocnemius (skeletal muscle), **C)** inguinal white adipose tissue (iWAT), **D)** gonadal white adipose tissue (gWAT), and **E)** Liver. Significant and/or relevant genes are labeled. Thermoneutral = 30°C housing condition.

bioRxiv preprint doi: https://doi.org/10.1101/2023.01.30.526183; this version posted January 31, 2023. The copyright holder for this preprint (which was not certified by peer review) is the author/funder, who has granted bioRxiv a license to display the preprint in perpetuity. It is made available under aCC-BY 4.0 International license.

Fig. S9

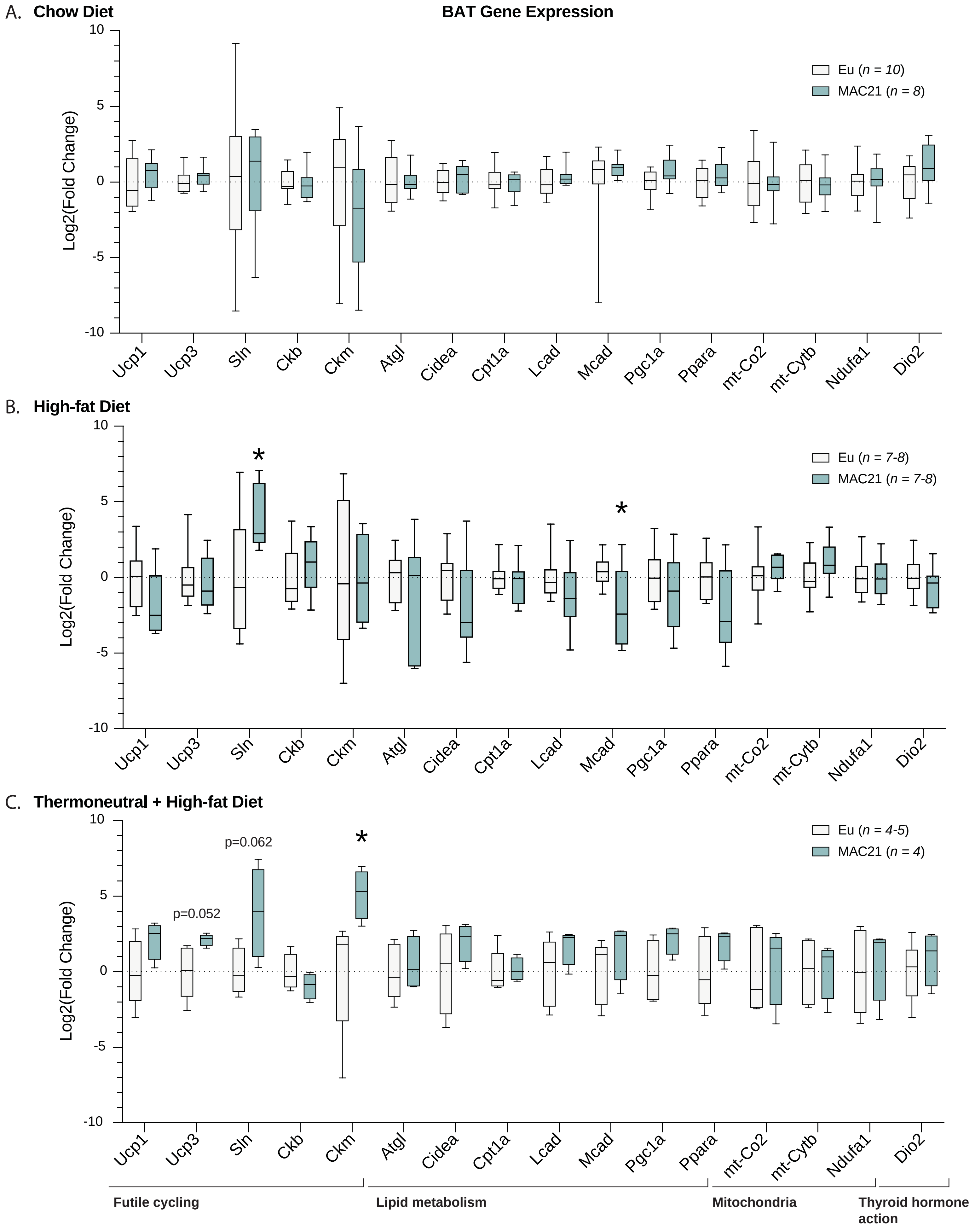

bioRxiv preprint doi: https://doi.org/10.1101/2023.01.30.526183; this version posted January 31, 2023. The copyright holder for this preprint (which was not certified by peer review) is the author/funder, who has granted bioRxiv a license to display the preprint in perpetuity. It is made available under aCC-BY 4.0 International license.

Figure S9. Gene expression analysis in brown adipose tissue. qPCR analysis of brown adipose tissue genes important for futile cycling, lipid metabolism, mitochondrial function, and thyroid hormone action in Euploid and MAC21 male mice fed a standard chow and housed at 22°C (A), fed a high-fat diet and housed at 22°C (B), and fed a high-fat diet and housed at thermoneutral 30°C (C)**.**

Fig. S10

bioRxiv preprint doi: https://doi.org/10.1101/2023.01.30.526183; this version posted January 31, 2023. The copyright holder for this preprint (which was not certified by peer review) is the author/funder, who has granted bioRxiv a license to display the preprint in perpetuity. It is made available under aCC-BY 4.0 International license.

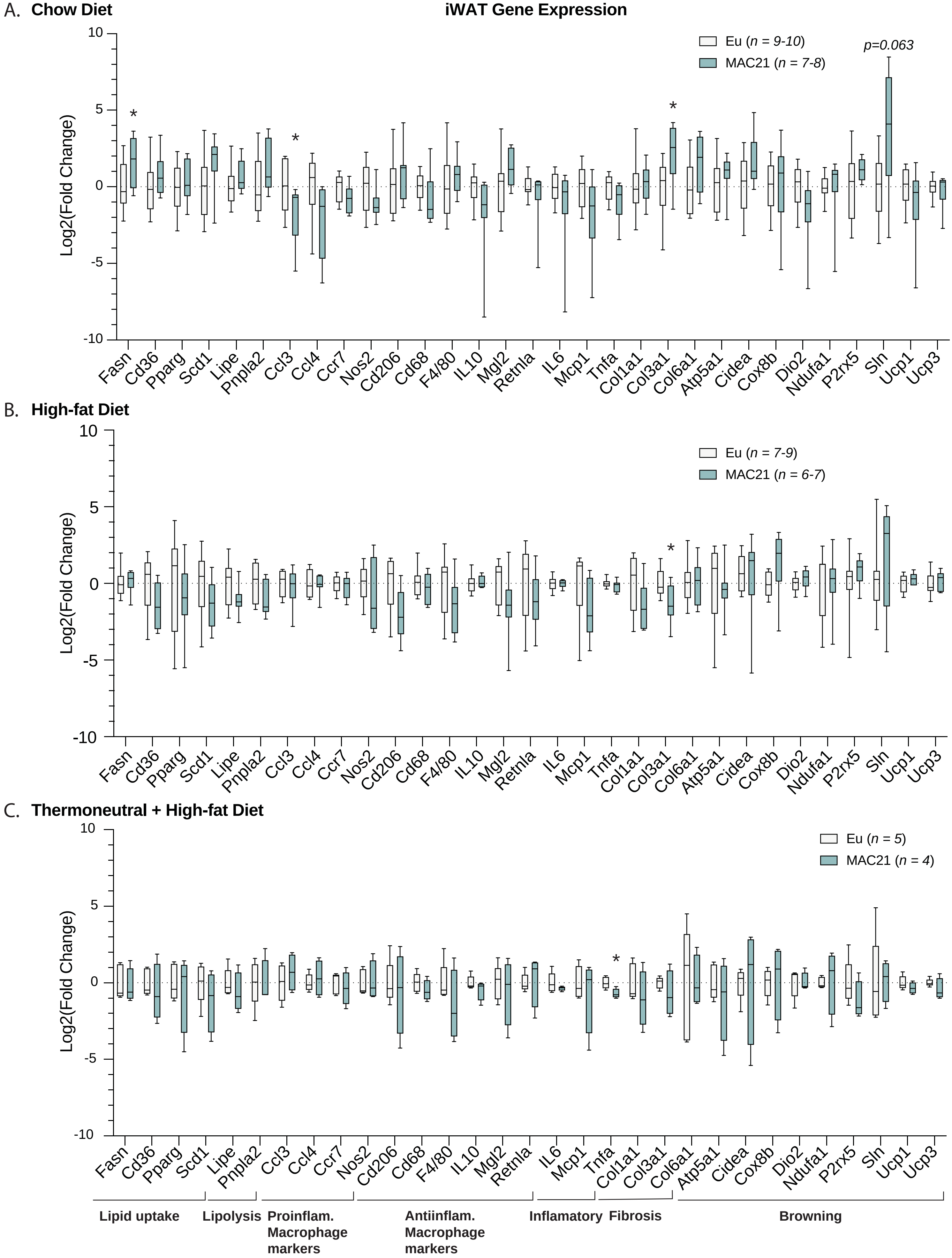

bioRxiv preprint doi: https://doi.org/10.1101/2023.01.30.526183; this version posted January 31, 2023. The copyright holder for this preprint (which was not certified by peer review) is the author/funder, who has granted bioRxiv a license to display the preprint in perpetuity. It is made available under aCC-BY 4.0 International license.

Figure S10. Gene expression analysis in inguinal white adipose tissue. qPCR analysis of inguinal white adipose tissue (iWAT) genes important for lipid uptake, lipolysis, inflammation, fibrosis, and browning in Euploid and MAC21 male mice fed a standard chow diet and housed at 22°C (A), fed a high-fat diet and housed at 22°C (B), and fed a high-fat diet and housed at thermoneutral 30°C (C)**.**

bioRxiv preprint doi: https://doi.org/10.1101/2023.01.30.526183; this version posted January 31, 2023. The copyright holder for this preprint (which was not certified by peer review) is the author/funder, who has granted bioRxiv a license to display the preprint in perpetuity. It is made available under aCC-BY 4.0 International license.

Fig. S11

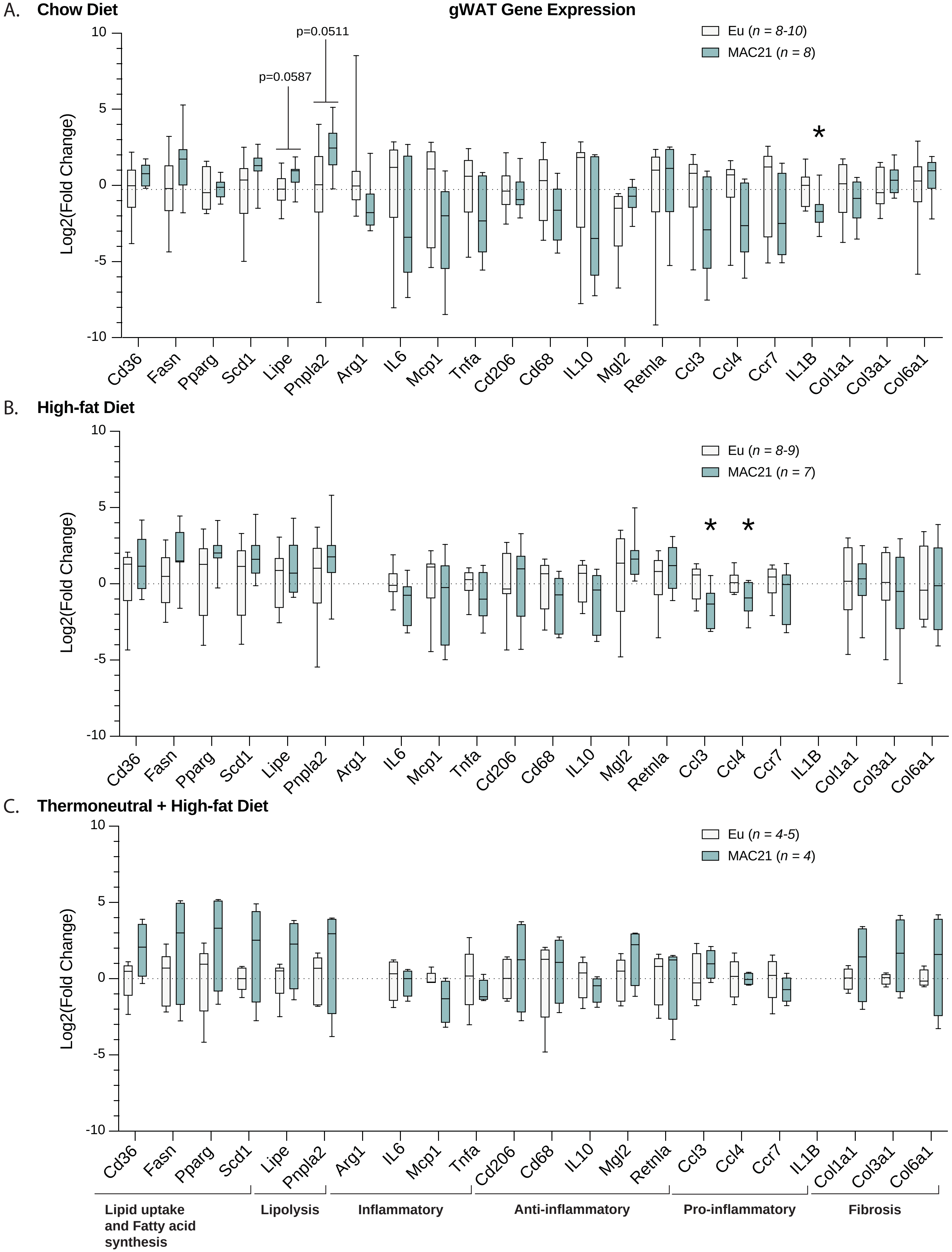

bioRxiv preprint doi: https://doi.org/10.1101/2023.01.30.526183; this version posted January 31, 2023. The copyright holder for this preprint (which was not certified by peer review) is the author/funder, who has granted bioRxiv a license to display the preprint in perpetuity. It is made available under aCC-BY 4.0 International license.

Figure S11. Gene expression analysis in gonadal white adipose tissue. qPCR analysis of gonadal white adipose tissue (gWAT) genes important for lipid uptake, fatty acid synthesis, lipolysis, inflammation, and fibrosis in Euploid and MAC21 male mice fed a standard chow diet and housed at 22°C (A), fed a high-fat diet and housed at 22°C (B), and fed a high-fat diet and housed at thermoneutral 30°C (C)**.**

bioRxiv preprint doi: https://doi.org/10.1101/2023.01.30.526183; this version posted January 31, 2023. The copyright holder for this preprint (which was not certified by peer review) is the author/funder, who has granted bioRxiv a license to display the preprint in perpetuity. It is made available under aCC-BY 4.0 International license.

Fig. S12

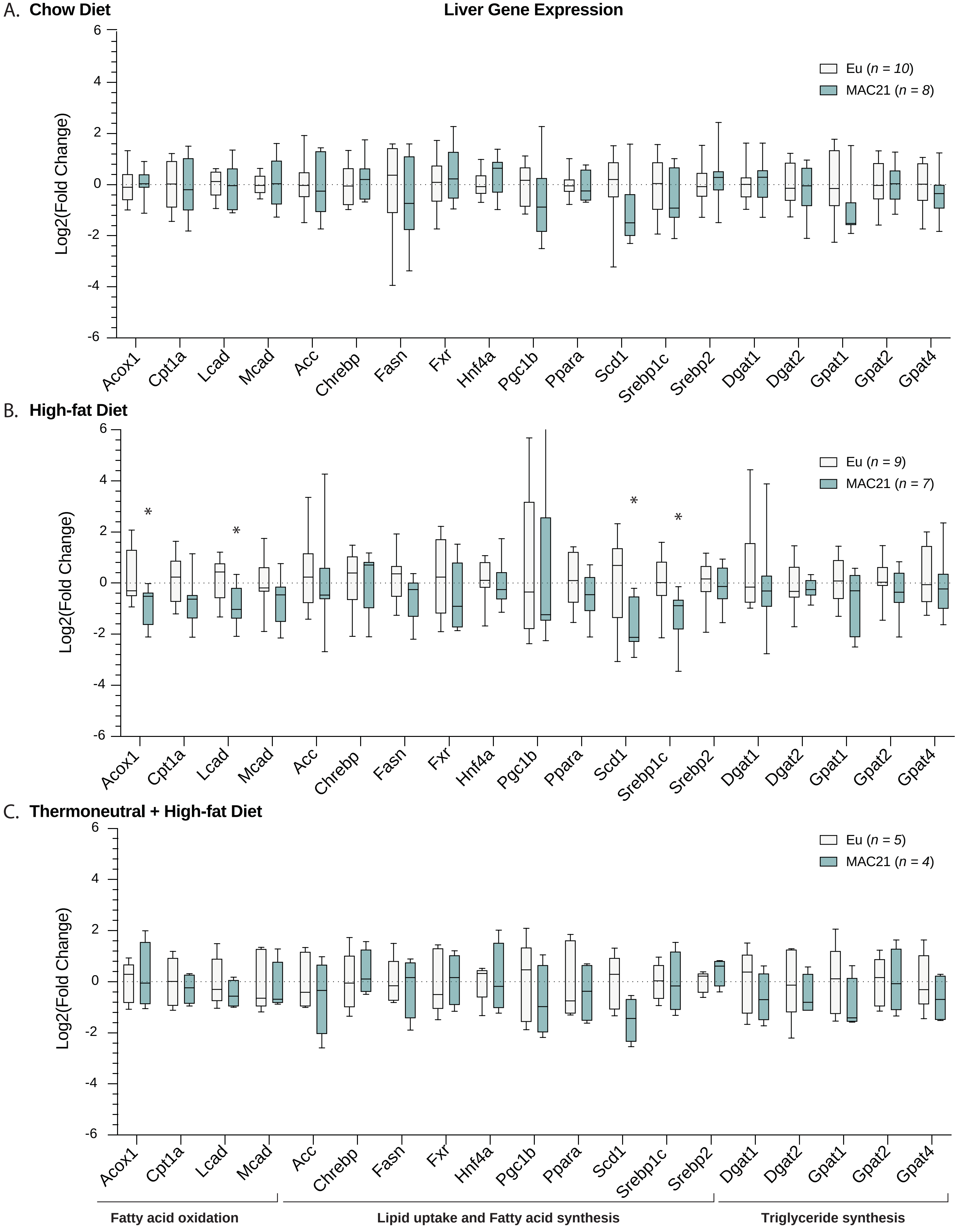

bioRxiv preprint doi: https://doi.org/10.1101/2023.01.30.526183; this version posted January 31, 2023. The copyright holder for this preprint (which was not certified by peer review) is the author/funder, who has granted bioRxiv a license to display the preprint in perpetuity. It is made available under aCC-BY 4.0 International license.

Figure S12. Gene expression analysis in liver. qPCR analysis of liver genes important for lipid uptake, fatty acid synthesis and oxidation, and triglyceride synthesis in Euploid and MAC21 male mice fed a standard chow and housed at 22°C (A), fed a high-fat diet and housed at 22°C (B), and fed a high-fat diet and housed at thermoneutral 30°C (C).

bioRxiv preprint doi: https://doi.org/10.1101/2023.01.30.526183; this version posted January 31, 2023. The copyright holder for this preprint (which was not certified by peer review) is the author/funder, who has granted bioRxiv a license to display the preprint in perpetuity. It is made available under aCC-BY 4.0 International license.

Fig. S13

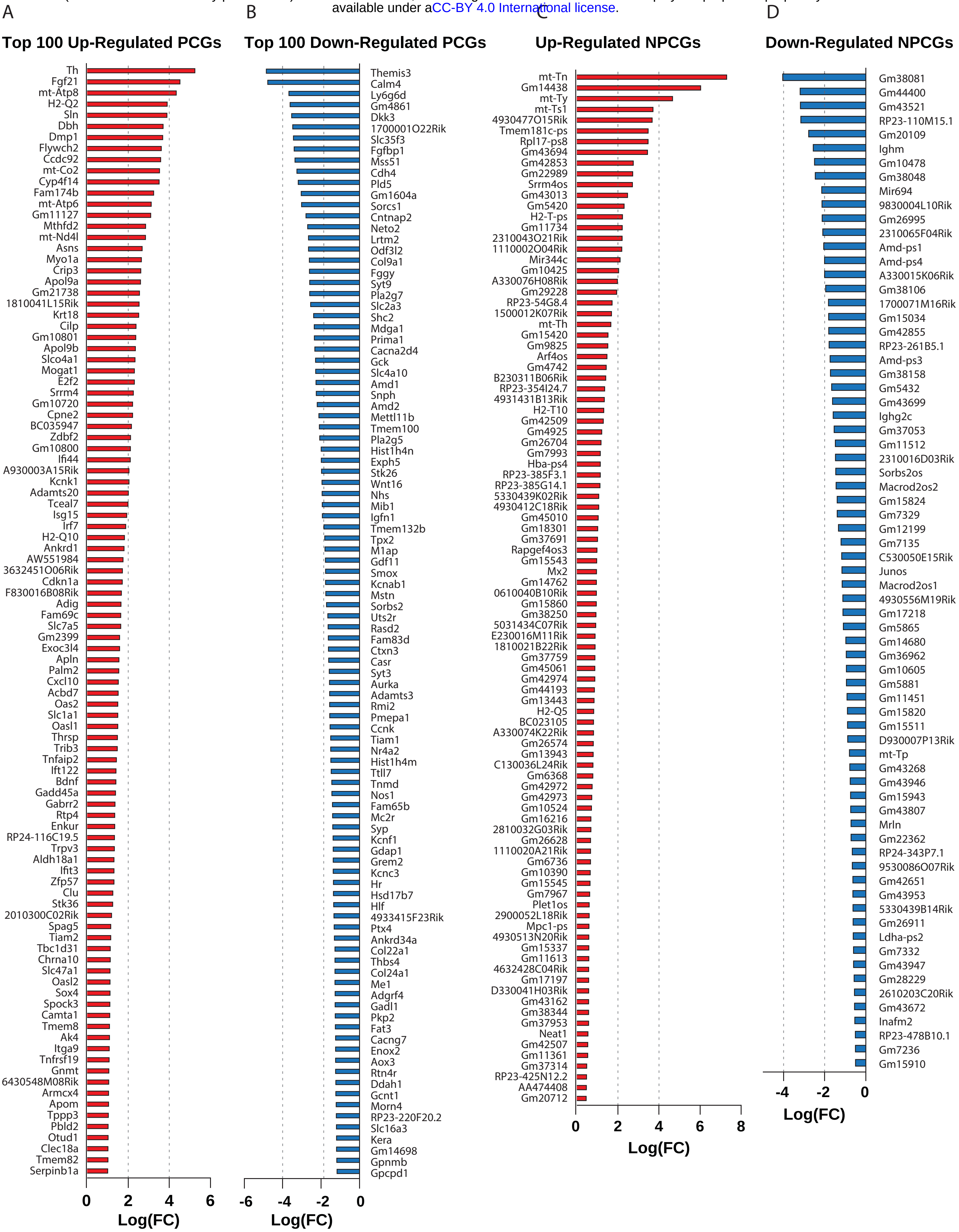

bioRxiv preprint doi: https://doi.org/10.1101/2023.01.30.526183; this version posted January 31, 2023. The copyright holder for this preprint (which was not certified by peer review) is the author/funder, who has granted bioRxiv a license to display the preprint in perpetuity. It is made available under aCC-BY 4.0 International license.

Figure S13. The top up- and down-regulated genes in skeletal muscle based on RNA-seq data. A) Top 100 up-regulated protein-coding genes. **B)** Top 100 down-regulated protein-coding genes. **C)** All up-regulated non-protein-coding genes. **D)** All down-regulated non-protein-coding genes. *n* = 5 Euploid and 4 MAC21 for RNA-sequencing experiments. All mice were fed a high-fat diet and housed at ambient room temperature (22°C).

bioRxiv preprint doi: https://doi.org/10.1101/2023.01.30.526183; this version posted January 31, 2023. The copyright holder for this preprint (which was not certified by peer review) is the author/funder, who has granted bioRxiv a license to display the preprint in perpetuity. It is made available under aCC-BY 4.0 International license.

Fig S14

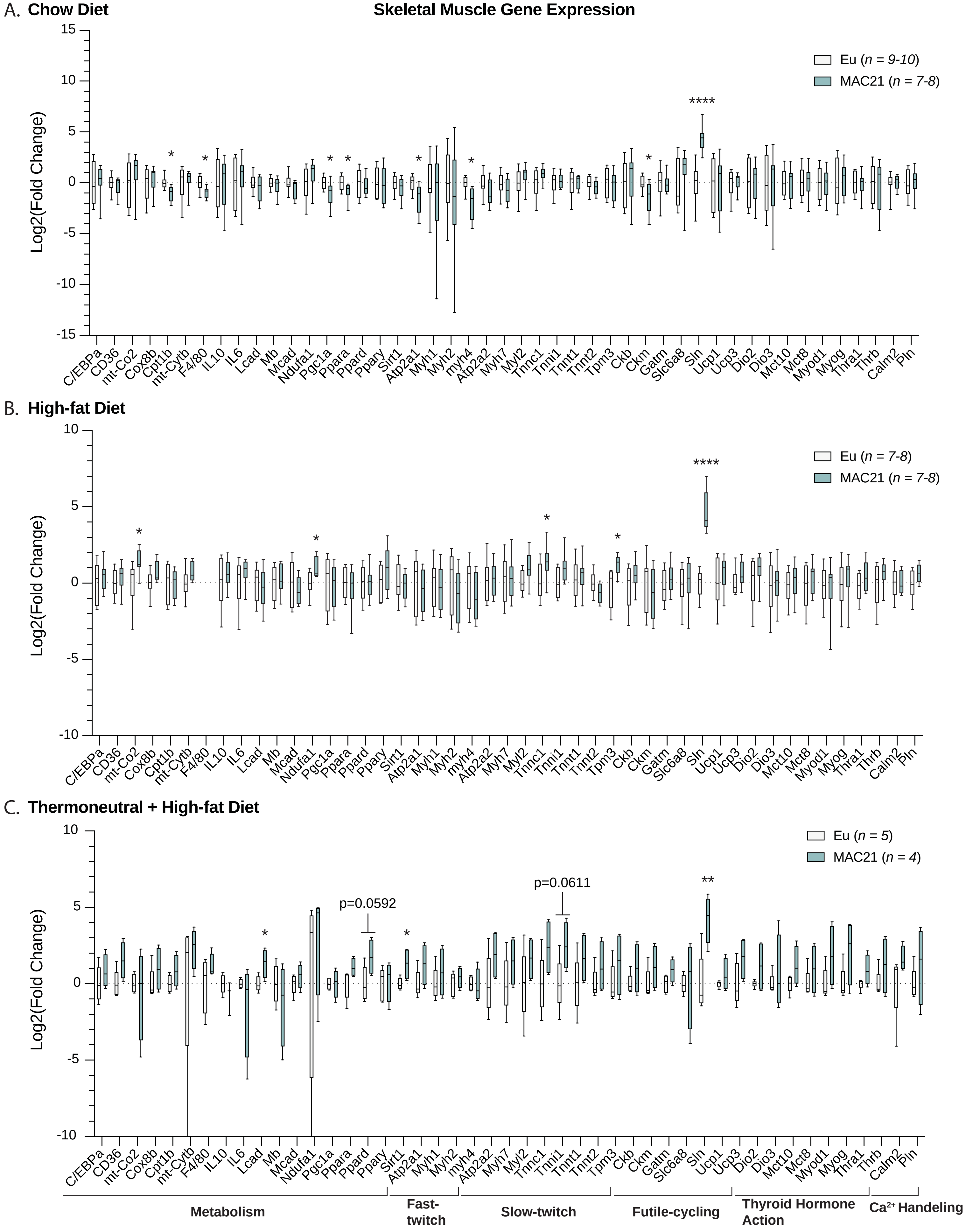

bioRxiv preprint doi: https://doi.org/10.1101/2023.01.30.526183; this version posted January 31, 2023. The copyright holder for this preprint (which was not certified by peer review) is the author/funder, who has granted bioRxiv a license to display the preprint in perpetuity. It is made available under aCC-BY 4.0 International license.

Figure S14. Gene expression analysis in skeletal muscle. qPCR analysis of gastrocnemius skeletal muscle genes important for general metabolism, fast- and slow-twitch fiber types, futile cycling, thyroid hormone action, and calcium handling in Euploid and MAC21 male mice fed a standard chow diet and housed at 22°C (A), fed a high-fat diet and housed at 22°C (B), and fed a high-fat diet and housed at thermoneutral 30°C (C)**.**

bioRxiv preprint doi: https://doi.org/10.1101/2023.01.30.526183; this version posted January 31, 2023. The copyright holder for this preprint (which was not certified by peer review) is the author/funder, who has granted bioRxiv a license to display the preprint in perpetuity. It is made available under aCC-BY 4.0 International license.

Fig. S15

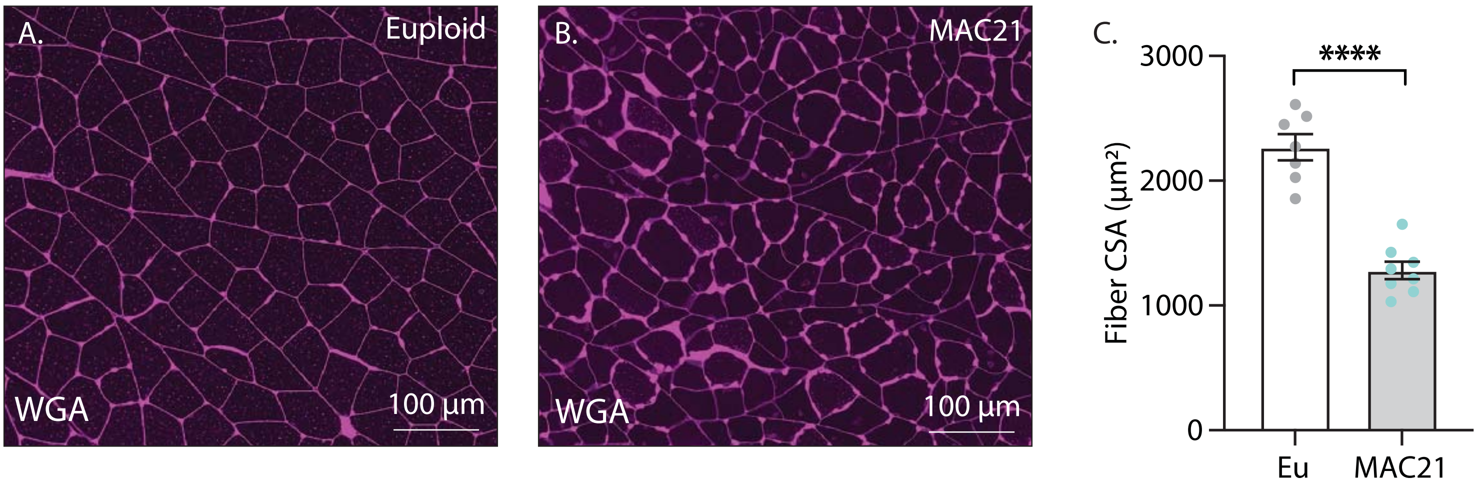

bioRxiv preprint doi: https://doi.org/10.1101/2023.01.30.526183; this version posted January 31, 2023. The copyright holder for this preprint (which was not certified by peer review) is the author/funder, who has granted bioRxiv a license to display the preprint in perpetuity. It is made available under aCC-BY 4.0 International license.

Figure S15. Skeletal muscle cross-sectional area analysis of MAC21 and Euploid mice fed a high-fat diet. A-B) Representative wheat-germ agglutinin (WGA) stained gastrocnemius skeletal muscle samples**. C)** Muscle fiber cross-sectional area (CSA) analysis. Mice were housed at ambient room temperature (22°C).

bioRxiv preprint doi: https://doi.org/10.1101/2023.01.30.526183; this version posted January 31, 2023. The copyright holder for this preprint (which was not certified by peer review) is the author/funder, who has granted bioRxiv a license to display the preprint in perpetuity. It is made available under aCC-BY 4.0 International license.

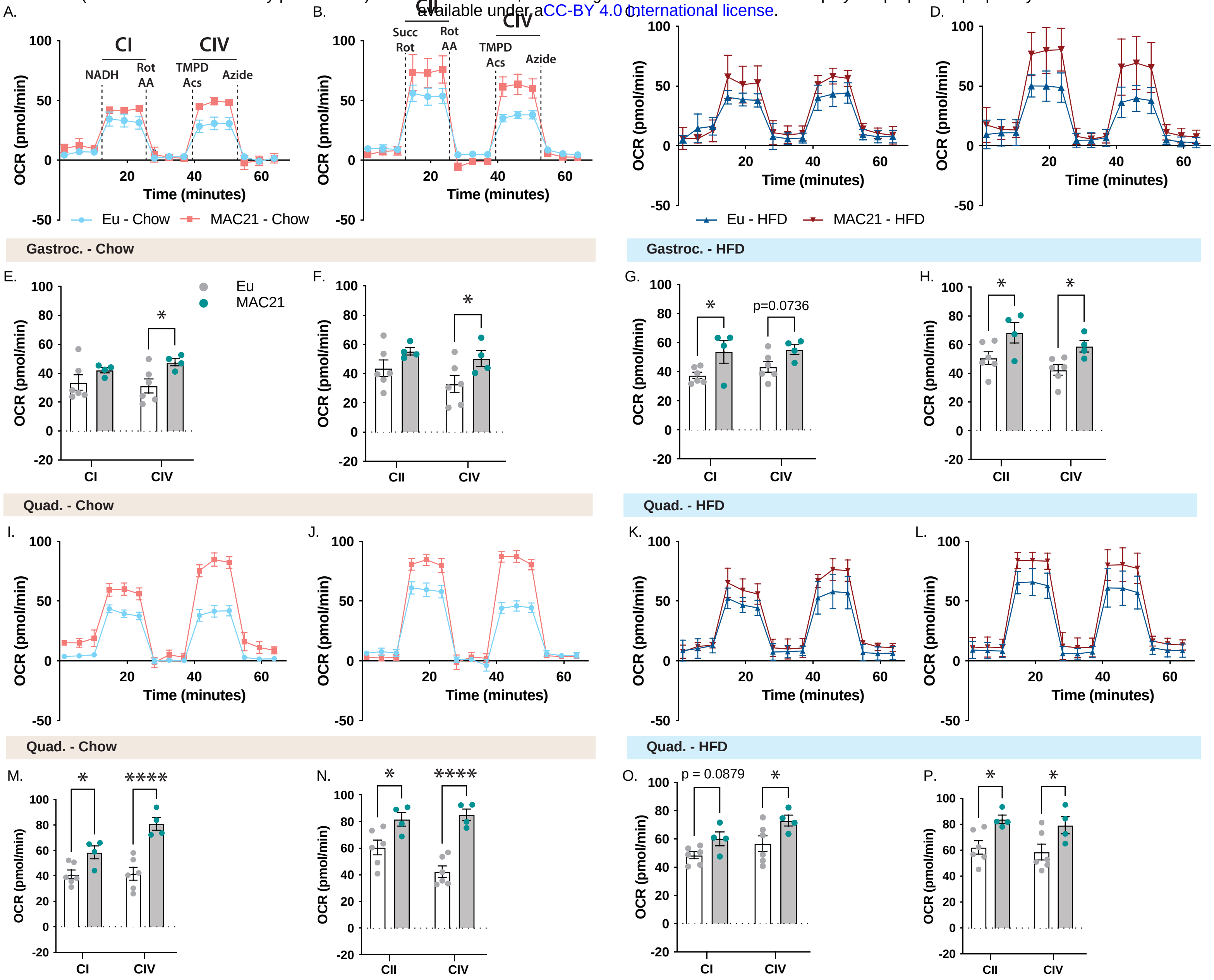

Fig. S16

bioRxiv preprint doi: https://doi.org/10.1101/2023.01.30.526183; this version posted January 31, 2023. The copyright holder for this preprint (which was not certified by peer review) is the author/funder, who has granted bioRxiv a license to display the preprint in perpetuity. It is made available under aCC-BY 4.0 International license.

Figure S16. Mitochondrial respirometry analysis of gastrocnemius and quadricep muscle. A and C) Average group gastrocnemius (Gastroc) oxygen consumption rate (OCR) traces using NADH as a substrate for Euploid (Eu) and MAC21 male mice fed a standard chow or high-fat diet (HFD), respectively. **B and D)** Average group Gastroc OCR traces using succinate as a substrate in the presence of rotenone (Rot) for Euploid and MAC21 mice fed a standard chow or HFD, respectively. **E and G)** Mitochondrial complex I, and IV OCR quantification for A and C, respectively. **F and H)** Mitochondrial complex II and IV OCR quantification for B and D, respectively. **I and K)** Average group quadricep (Quad) OCR traces using NADH as a substrate for Euploid and MAC21 mice fed a standard chow or HFD, respectively. **J and L)** Average group Quad OCR traces using succinate as a substrate in the presence of rotenone (Rot) for Euploid and MAC21 mice fed a standard chow or HFD, respectively. **M and O)** Mitochondrial complex I and IV OCR quantification for I and K, respectively. **N and P)** Mitochondrial complex II and IV OCR quantification for J and L, respectively. All mice used for respirometry were house at ambient room temperature (22°C). AA, antimycin A; TMPD, N,N,N',N'-tetramethyl-p-phenylenediamine; Acs, ascorbate; CI, mitochondrial complex I; CII, mitochondrial complex II; CIV, mitochondrial complex IV.

bioRxiv preprint doi: https://doi.org/10.1101/2023.01.30.526183; this version posted January 31, 2023. The copyright holder for this preprint (which was not certified by peer review) is the author/funder, who has granted bioRxiv a license to display the preprint in perpetuity. It is made available under aCC-BY 4.0 International license.

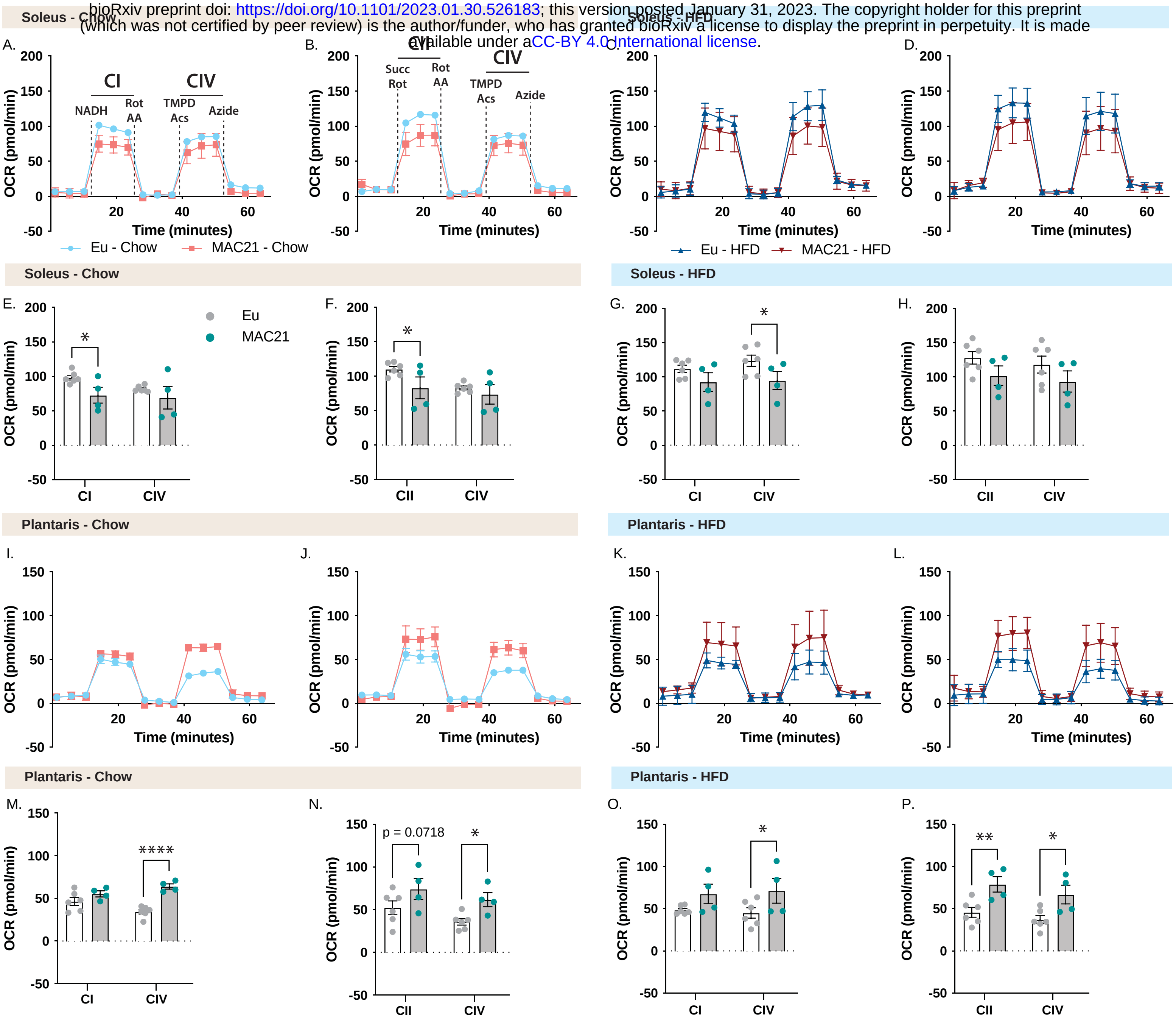

Fig. S17

bioRxiv preprint doi: https://doi.org/10.1101/2023.01.30.526183; this version posted January 31, 2023. The copyright holder for this preprint (which was not certified by peer review) is the author/funder, who has granted bioRxiv a license to display the preprint in perpetuity. It is made available under aCC-BY 4.0 International license.

Figure S17. Mitochondrial respirometry analysis of soleus and plantaris muscle. A and C) Average group soleus oxygen consumption rate (OCR) traces using NADH as a substrate for Euploid (Eu) and MAC21 male mice fed a standard chow or high-fat diet (HFD), respectively. **B and D)** Average group soleus OCR traces using succinate as a substrate in the presence of Rotenone (Rot) for Euploid and MAC21 mice fed a standard chow or HFD, respectively. **E and G)** Mitochondrial complex I and IV OCR quantification for A and C, respectively. **F and H)** Mitochondrial complex II and IV OCR quantification for B and D, respectively. **I and K)** Average group plantaris OCR traces using NADH as a substrate for Euploid and MAC21 mice fed a standard chow or HFD, respectively. **J and L)** Average group plantaris OCR traces using succinate as a substrate in the presence of rotenone (Rot) for Euploid and MAC21 mice fed a standard chow or HFD, respectively. **M and O)** Mitochondrial complex I and IV OCR quantification for I and K, respectively. **N and P)** Mitochondrial complex II and IV OCR quantification for J and L, respectively. All mice used for respirometry were house at ambient room temperature (22°C). AA, antimycin A; TMPD, N,N,N’,N’-tetramethyl-p-phenylenediamine; Acs, ascorbate; CI, mitochondrial complex I; CII, mitochondrial complex II; CIV, mitochondrial complex IV.

bioRxiv preprint doi: https://doi.org/10.1101/2023.01.30.526183; this version posted January 31, 2023. The copyright holder for this preprint (which was not certified by peer review) is the author/funder, who has granted bioRxiv a license to display the preprint in perpetuity. It is made available under aCC-BY 4.0 International license.

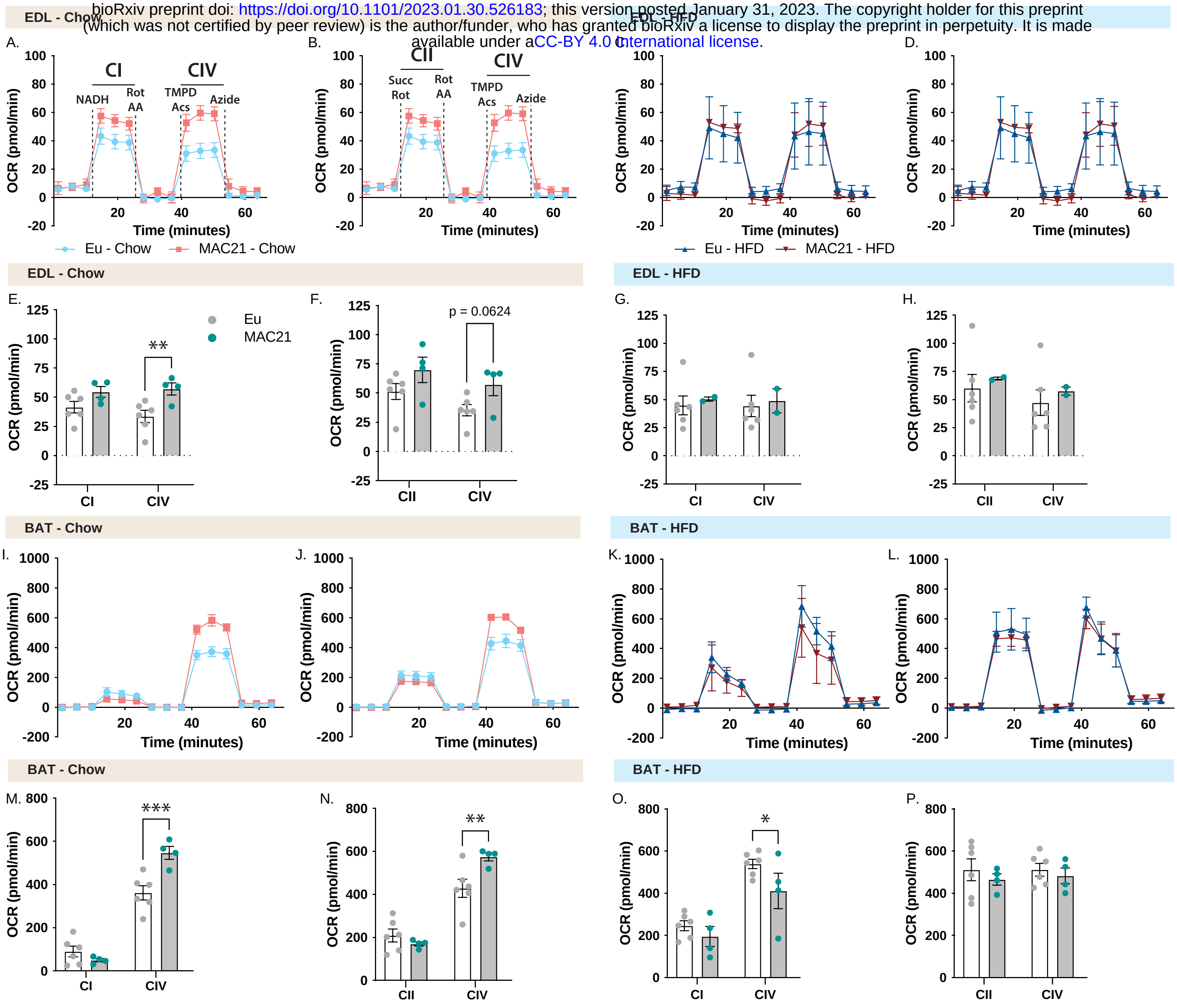

Fig. S18

bioRxiv preprint doi: https://doi.org/10.1101/2023.01.30.526183; this version posted January 31, 2023. The copyright holder for this preprint (which was not certified by peer review) is the author/funder, who has granted bioRxiv a license to display the preprint in perpetuity. It is made available under aCC-BY 4.0 International license.

Figure S18. Mitochondrial respirometry analysis of EDL and BAT. A and C) Average group extensor digitorum longus (EDL) oxygen consumption rate (OCR) traces using NADH as a substrate for Euploid (Eu) and MAC21 male mice fed a standard chow or high-fat diet (HFD), respectively. **B and D)** Average group EDL OCR traces using succinate as a substrate in the presence of rotenone (Rot) for Euploid and MAC21 mice fed a standard chow or HFD, respectively. **E and G)** Mitochondrial complex I and IV OCR quantification for A and C, respectively. **F and H)** Mitochondrial complex II and IV OCR quantification for B and D, respectively. **I and K)** Average group brown adipose tissue (BAT) OCR traces using NADH as a substrate for Euploid and MAC21 mice fed a standard chow or HFD, respectively. **J and L)** Average group brown adipose tissue (BAT) OCR traces using succinate as a substrate in the presence of rotenone (Rot) for Euploid and MAC21 mice fed a standard chow or HFD, respectively. **M and O)** Mitochondrial complex I and IV OCR quantification for I and K, respectively. **N and P)** Mitochondrial complex II and IV OCR quantification for J and L, respectively. All mice used for respirometry were house at ambient room temperature (22°C). AA, antimycin A; TMPD, N,N,N',N'-tetramethyl-p-phenylenediamine; Acs, ascorbate; CI, mitochondrial complex I; CII, mitochondrial complex II; CIV, mitochondrial complex IV.

bioRxiv preprint doi: https://doi.org/10.1101/2023.01.30.526183; this version posted January 31, 2023. The copyright holder for this preprint (which was not certified by peer review) is the author/funder, who has granted bioRxiv a license to display the preprint in perpetuity. It is made available under aCC-BY 4.0 International license.

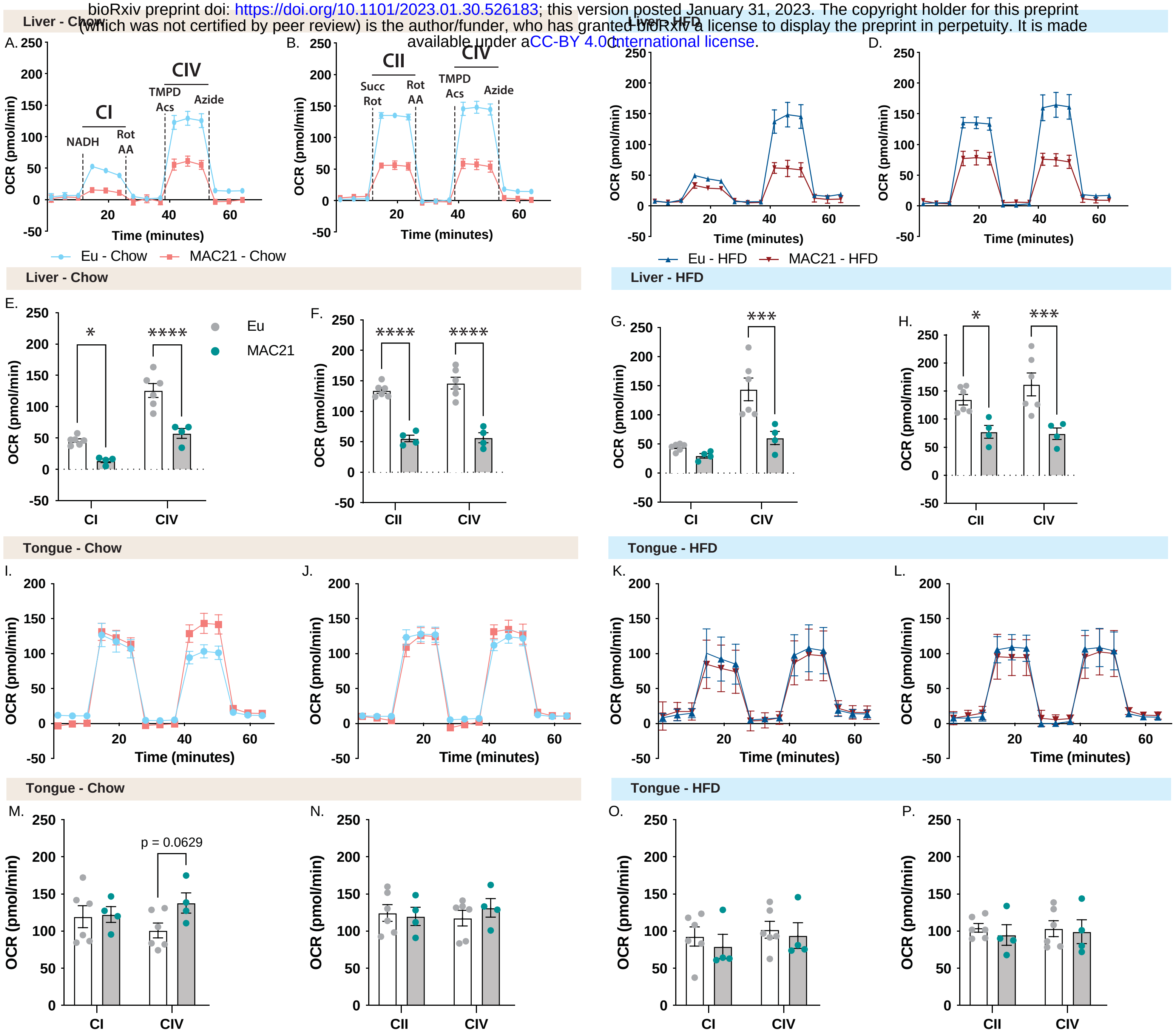

Fig. S19

bioRxiv preprint doi: https://doi.org/10.1101/2023.01.30.526183; this version posted January 31, 2023. The copyright holder for this preprint (which was not certified by peer review) is the author/funder, who has granted bioRxiv a license to display the preprint in perpetuity. It is made available under aCC-BY 4.0 International license.

Figure S19. Mitochondrial respirometry analysis of liver and tongue. A and C) Average group liver oxygen consumption rate (OCR) traces using NADH as a substrate for Euploid (Eu) and MAC21 male mice fed a standard chow or high-fat diet (HFD), respectively. **B and D)** Average group liver OCR traces using succinate as a substrate in the presence of rotenone (Rot) for Euploid and MAC21 mice fed a standard chow or HFD, respectively. **E and G)** Mitochondrial complex I and IV OCR quantification for A and C, respectively. **F and H)** Mitochondrial complex II and IV OCR quantification for B and D, respectively. **I and K)** Average group tongue OCR traces using NADH as a substrate for Euploid and MAC21 mice fed a standard chow or HFD, respectively. **J and L)** Average group tongue OCR traces using succinate as a substrate in the presence of rotenone (Rot) for Euploid and MAC21 mice fed a standard chow or HFD, respectively. **M and O)** Mitochondrial complex I and IV OCR quantification for I and K, respectively. **N and P)** Mitochondrial complex II and IV OCR quantification for J and L, respectively. All mice used for respirometry were house at ambient room temperature (22°C). AA, antimycin A; TMPD, N,N,N',N'-tetramethyl-p-phenylenediamine; Acs, ascorbate; CI, mitochondrial complex I; CII, mitochondrial complex II; CIV, mitochondrial complex IV.

bioRxiv preprint doi: https://doi.org/10.1101/2023.01.30.526183; this version posted January 31, 2023. The copyright holder for this preprint (which was not certified by peer review) is the author/funder, who has granted bioRxiv a license to display the preprint in perpetuity. It is made available under aCC-BY 4.0 International license.

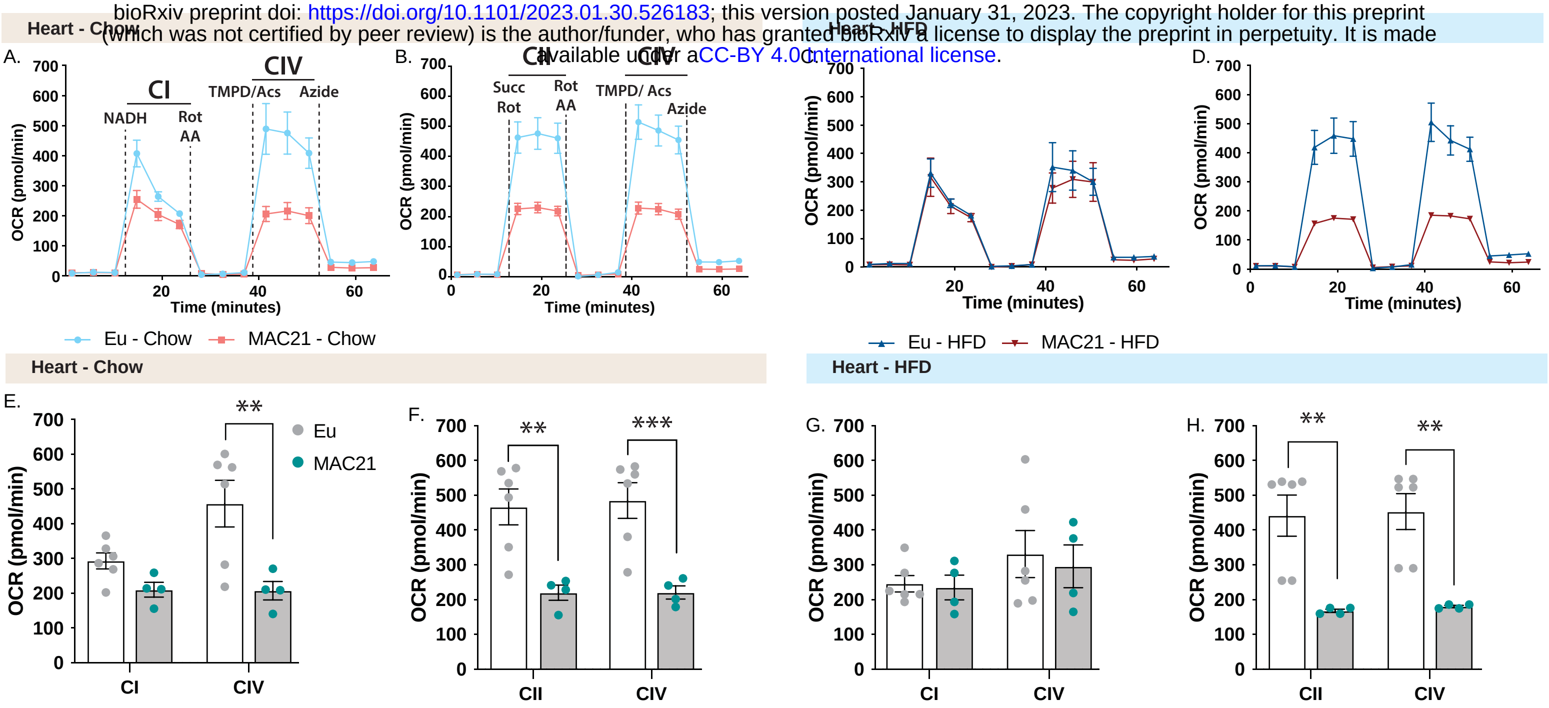

Fig. S20

bioRxiv preprint doi: https://doi.org/10.1101/2023.01.30.526183; this version posted January 31, 2023. The copyright holder for this preprint (which was not certified by peer review) is the author/funder, who has granted bioRxiv a license to display the preprint in perpetuity. It is made available under aCC-BY 4.0 International license.

Figure S20. Mitochondrial respirometry analysis of heart. A and C) Average group heart oxygen consumption rate (OCR) traces using NADH as a substrate for Euploid (Eu) and MAC21 male mice fed a standard chow or high-fat diet (HFD), respectively. **B and D)** Average group heart OCR traces using succinate as a substrate in the presence of rotenone (Rot) for Euploid and MAC21 mice fed a standard chow or HFD, respectively. **E and G)** Mitochondrial complex I and IV OCR quantification for A and C, respectively. **F and H)** Mitochondrial complex II and IV OCR quantification for B and D, respectively. All mice used for respirometry were house at ambient room temperature (22°C). AA, antimycin A; TMPD, N,N,N’,N’-tetramethyl-p-phenylenediamine; Acs, ascorbate; CI, mitochondrial complex I; CII, mitochondrial complex II; CIV, mitochondrial complex IV.

bioRxiv preprint doi: https://doi.org/10.1101/2023.01.30.526183; this version posted January 31, 2023. The copyright holder for this preprint (which was not certified by peer review) is the author/funder, who has granted bioRxiv a license to display the preprint in perpetuity. It is made available under aCC-BY 4.0 International license.

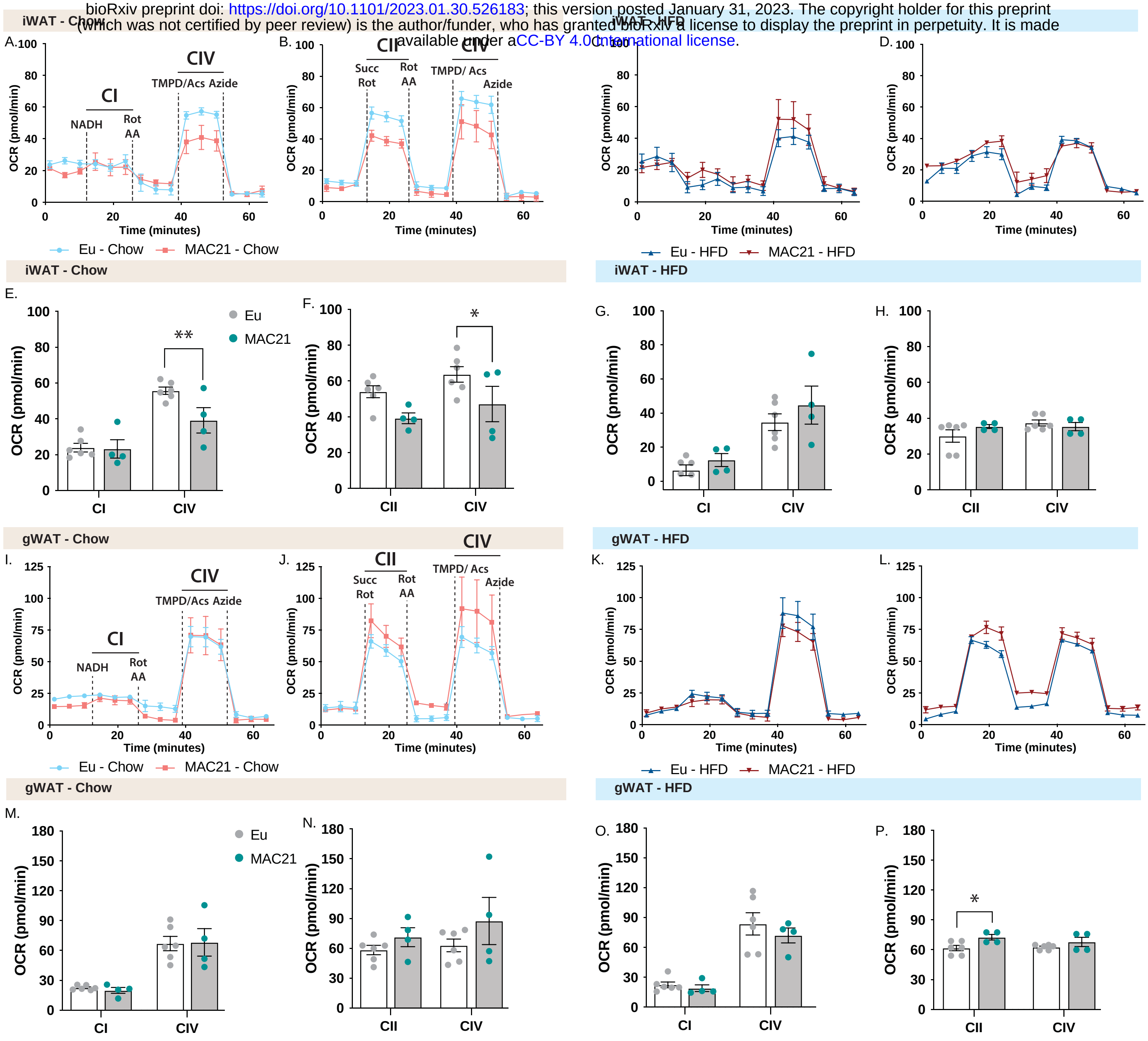

Fig. S21

bioRxiv preprint doi: https://doi.org/10.1101/2023.01.30.526183; this version posted January 31, 2023. The copyright holder for this preprint (which was not certified by peer review) is the author/funder, who has granted bioRxiv a license to display the preprint in perpetuity. It is made available under aCC-BY 4.0 International license.

Figure S21. Mitochondrial respirometry analysis of inguinal (iWAT) and gonadal (gWAT) white adipose tissue. A and C) Average group iWAT oxygen consumption rate (OCR) traces using NADH as a substrate for Euploid (Eu) and MAC21 male mice fed a standard chow or high-fat diet (HFD), respectively. **B and D)** Average group liver OCR traces using succinate as a substrate in the presence of rotenone (Rot) for Euploid and MAC21 mice fed a standard chow or HFD, respectively. **E and G)** Mitochondrial complex I and IV OCR quantification for A and C, respectively. **F and H)** Mitochondrial complex II and IV OCR quantification for B and D, respectively. **I and K)** Average group gWAT OCR traces using NADH as a substrate for Euploid and MAC21 mice fed a standard chow or HFD, respectively. **J and L)** Average group gWAT OCR traces using succinate as a substrate in the presence of rotenone (Rot) for Euploid and MAC21 mice fed a standard chow or HFD, respectively. **M and O)** Mitochondrial complex I and IV OCR quantification for I and K, respectively. **N and P)** Mitochondrial complex II and IV OCR quantification for J and L, respectively. All mice used for respirometry were house at ambient room temperature (22°C). AA, antimycin A; TMPD, N,N,N',N'-tetramethyl-p-phenylenediamine; Acs, ascorbate; CI, mitochondrial complex I; CII, mitochondrial complex II; CIV, mitochondrial complex IV.

bioRxiv preprint doi: https://doi.org/10.1101/2023.01.30.526183; this version posted January 31, 2023. The copyright holder for this preprint (which was not certified by peer review) is the author/funder, who has granted bioRxiv a license to display the preprint in perpetuity. It is made available under aCC-BY 4.0 International license.

Fig. S22

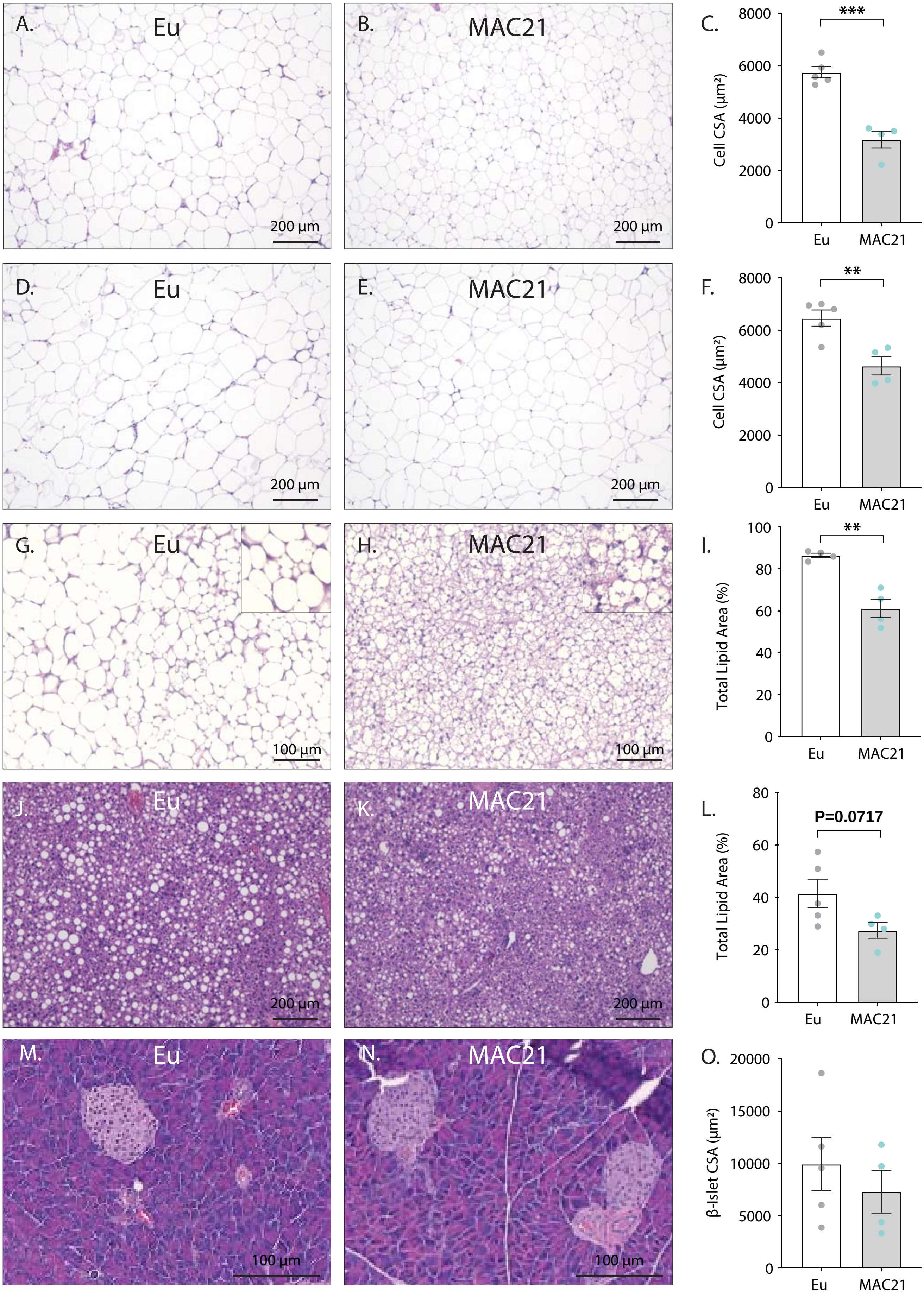

bioRxiv preprint doi: https://doi.org/10.1101/2023.01.30.526183; this version posted January 31, 2023. The copyright holder for this preprint (which was not certified by peer review) is the author/funder, who has granted bioRxiv a license to display the preprint in perpetuity. It is made available under aCC-BY 4.0 International license.

Figure S22. Histology of HFD-fed Euploid and MAC21 mice housed at thermoneutrality (30°C).

Representative hematoxylin and eosin (H&E) stained sections of inguinal white adipose tissue (iWAT, A-B), gonadal white adipose tissue (gWAT, D and E), brown adipose tissue (BAT, H and I), liver (J and K), and pancreas (M and N). **C)** Average iWAT adipocyte cross-sectional area (CSA) quantification. **F)** Average gWAT adipocyte CSA quantification. **I)** Average BAT area covered by lipid droplets per focal plane. **L)** Average liver area covered by lipid droplets per focal plane. **O)** Average pancreas β-islet CSA quantification.

bioRxiv preprint doi: https://doi.org/10.1101/2023.01.30.526183; this version posted January 31, 2023. The copyright holder for this preprint (which was not certified by peer review) is the author/funder, who has granted bioRxiv a license to display the preprint in perpetuity. It is made available under aCC-BY 4.0 International license.

Table S1. Tissue weights of chow-fed Euploid and MAC21 male mice at termination of the study.

	Eu	MAC21	*P*
N	7	6	
BW (g)	**47.17 ± 3.73**	**26.77 ± 3.14**	**0.002**
gWAT (g)	**1.47 ± 0.29**	**0.60 ± 0.19**	**0.040**
iWAT (g)	1.15 ± 0.30	0.43 ± 0.20	0.080
Liver (g)	**2.42 ± 0.31**	**1.54 ± 0.18**	**0.034**
Pancreas (g)	0.30 ± 0.02	0.27 ± 0.01	0.109
Kidney (g)	0.19 ± 0.01	0.15 ± 0.02	0.051
Heart (g)	**0.16 ± 0.01**	**0.14 ± 0.01**	**0.025**

Eu: Euploid; BW: body weight; gWAT: gondal white adipose tissue; iWAT: inguinal white adipose tissue; BAT: brown adipose tissue

Table S2. Indirect calorimetry analysis of male (16.5 weeks of age) Euploid and MAC21 littermate mice fed a standard chow

	Eu	MAC21	*P*	Eu	MAC21	*P*	Eu	MAC21	*P*
**Low-fat chow diet (Male)**	***Ad-libitum* (dark cycle)**			***Ad-libitum* (light cycle)**			***Ad-libitum* (24 hr)**		
N	8	9		8	9		8	9	
Body weight (g)							**28.9 ± 1.09**	**20.6 ± 0.29**	**<0.001**
Food intake (kcal)	9.76 ± 0.65	10.66 ± 0.58	0.320	2.44 ± 0.45	2.67 ± 0.20	0.639	12.2 ± 0.92	13.3 ± 0.56	0.302
VO2 (mL/kg lean mass/h)	**4381 ± 180**	**5674 ± 69**	**<0.001**	**3589 ± 123**	**4616 ± 83**	**<0.001**	**3984 ± 151**	**5145 ± 79**	**<0.001**
VCO2 (mL/kg lean mass/h)	**4231 ± 182**	**5600 ± 97**	**<0.001**	**3158 ± 122**	**4170 ± 109**	**<0.001**	**3695 ± 150**	**4889 ± 81**	**<0.001**
RER (VCO2/VO2)	**0.96 ± 0.006**	**0.99 ± 0.007**	**0.030**	0.88 ± 0.008	0.90 ± 0.010	0.099	**0.92 ± 0.006**	**0.94 ± 0.005**	**0.011**
EE (kcal/kg lean mass/h)	**21.9 ± 0.91**	**28.5 ± 0.49**	**<0.001**	**17.6 ± 0.62**	**22.7 ± 0.45**	**<0.001**	**19.8 ± 0.76**	**25.6 ± 0.40**	**<0.001**
Total activity (beam breaks)	**30160 ± 2377**	**47055 ± 5532**	**0.017**	**9639 ± 1589**	**14530 ± 1107**	**0.021**	**39798 ± 3869**	**61586 ± 6366**	**0.013**
Ambulatory activity (counts)	**16577 ± 3138**	**28148 ± 4020**	**0.042**	4605 ± 1662	7135 ± 778	0.172	21181 ± 4736	35283 ± 4680	0.052
	**Fasting (dark cycle)**			**Fasting (light cycle)**			**Fasting (24 hr)**		
Body weight (g)							**25.5 ± 1.15**	**16.6 ± 0.41**	**<0.001**
Food intake (kcal)	0	0		0	0		0	0	
VO2 (mL/kg lean mass/h)	**3604 ± 118**	**5151 ± 149**	**<0.001**	**2794 ± 60**	**4099 ± 199**	**<0.001**	**3199 ± 83**	**4675 ± 169**	**<0.001**
VCO2 (mL/kg lean mass/h)	**2800 ± 98**	**4115 ± 106**	**<0.001**	**2107 ± 57**	**3098 ± 161**	**<0.001**	**2454 ± 71**	**3606 ± 129**	**<0.001**
RER (VCO2/VO2)	0.77 ± 0.002	0.78 ± 0.006	0.105	0.75 ± 0.006	0.75 ± 0.008	0.982	0.76 ± 0.003	0.77 ± 0.006	0.492
EE (kcal/kg lean mass/h)	**17.2 ± 0.57**	**25.1 ± 0.69**	**<0.001**	**13.3 ± 0.30**	**19.5 ± 0.95**	**<0.001**	**15.2 ± 0.41**	**22.3 ± 0.80**	**<0.001**
Total activity (beam breaks)	**36169 ± 1838**	**87131 ± 11695**	**0.001**	**9221 ± 1546**	**56436 ± 14366**	**0.008**	**45389 ± 3068**	**143567 ± 25225**	**0.002**
Ambulatory activity (counts)	**21867 ± 1863**	**62399 ± 9337**	**0.001**	**4886 ± 1252**	**42324 ± 11664**	**0.009**	**26753 ± 2956**	**104723 ± 20414**	**0.003**
	**Re-feed (dark cycle)**			**Re-feed (light cycle)**			**Re-feed (24 hr)**		
Body weight (g)							**27.9 ± 1.03**	**19.4 ± 0.55**	**<0.001**
Food intake (kcal)	12.9 ± 0.62	13.4 ± 0.36	0.524	3.41 ± 0.37	4.01 ± 0.47	0.337	17.6 ± 1.05	17.3 ± 0.49	0.836
VO2 (mL/kg lean mass/h)	**4216 ± 145**	**4980 ± 184**	**0.006**	**3459 ± 154**	**4356 ± 126**	**<0.001**	**3850 ± 147**	**4678 ± 132**	**<0.001**
VCO2 (mL/kg lean mass/h)	**4200 ± 159**	**4966 ± 179**	**0.008**	**3502 ± 186**	**4431 ± 177**	**0.003**	**3873 ± 167**	**4706 ± 132**	**0.001**
RER (VCO2/VO2)	1.00 ± 0.005	1.00 ± 0.006	0.517	1.01 ± 0.014	1.01 ± 0.020	0.920	1.01 ± 0.009	1.00 ± 0.011	0.921
EE (kcal/kg lean mass/h)	**21.3 ± 0.75**	**25.1 ± 0.92**	**0.006**	**17.5 ± 0.81**	**22.1 ± 0.69**	**<0.001**	**19.5 ± 0.76**	**23.6 ± 0.66**	**<0.001**
Total activity (beam breaks)	**49750 ± 4033**	**65119 ± 5703**	**0.048**	9175 ± 1219	12966 ± 1506	0.073	37102 ± 4356	45853 ± 7582	0.349
Ambulatory activity (counts)	27781 ± 4810	38270 ± 3686	0.099	4326 ± 1186	6693 ± 1045	0.153	19302 ± 4436	26753 ± 5461	0.314

Eu: Euploid; VO2: rate of oxygen consumption; VCO2: rate of carbon dioxide production; RER: respiratory exchange ratio; EE: energy expenditure

bioRxiv preprint doi: https://doi.org/10.1101/2023.01.30.526183; this version posted January 31, 2023. The copyright holder for this preprint (which was not certified by peer review) is the author/funder, who has granted bioRxiv a license to display the preprint in perpetuity. It is made available under aCC-BY 4.0 International license.

Table S3. Tissue weight of high fat diet-fed male mice (33 weeks of age; fed diet for 16.5 weeks) at termination of the study

	**Eu**	**MAC21**	***P***
N	8	8	
Body weight (g)	**46.62 ± 2.16**	**28.16 ±1.76**	**<0.001**
Gondal fat (gWAT, g)	**1.09 ±0.11**	**0.64 ±0.12**	**0.016**
Inguinal fat (iWAT, g)	**1.02 ±0.06**	**0.32 ±0.08**	**<0.001**
Liver (g)	**2.02 ±0.22**	**1.39 ±0.08**	**0.018**
Pancreas (g)	0.25 ±0.03	0.25 ±0.01	0.933
Kidney (g)	**0.18 ±0.01**	**0.15 ±0.01**	**0.026**
Spleen (g)	0.09 ±0.01	0.09 ±0.01	0.851
Diaphragm (g)	**0.11 ±0.01**	**0.09 ±0.01**	**0.010**
Heart (g)	0.18 ±0.02	0.16 ±0.01	0.266
BAT (g)	**0.32 ±0.04**	**0.12 ±0.02**	**0.001**
Brain (g)	**0.45 ±0.01**	**0.48 ±0.005**	**0.004**
Tibia (mm)	18.25 ±0.21	18.04 ±0.15	0.421

Eu: Euploidy; gWAT: gondal white adipose tissue; iWAT: inguinal white adipose tissue; BAT: brown adipose tissue

bioRxiv preprint doi: https://doi.org/10.1101/2023.01.30.526183; this version posted January 31, 2023. The copyright holder for this preprint (which was not certified by peer review) is the author/funder, who has granted bioRxiv a license to display the preprint in perpetuity. It is made available under aCC-BY 4.0 International license.

Table S4. Complete blood count of Euploid and MAC21 mice fed a high-fat diet (50 weeks of age; on diet for 13 weeks)

	**Eu**	**MAC21**	***P***
N	5	6	
RBC (10^6/μL)	8.66±0.61	9.22±0.26	0.452
HGB (g/dL)	12.85±1.24	14.02±0.31	0.424
HCT (%)	41.88±3.59	42.88±1.09	0.813
MCV (fL)	48.12±1.46	46.56±0.54	0.381
MCH (pg)	14.72±0.59	15.26±0.16	0.436
MCHC (g/dL)	30.53±0.58	32.80±0.28	0.009
RET (10^3/μL)	747.12±141.32	412.34±25.07	0.063
PLT (10^3/μL)	722.33±176.68	1230.40±86.03	0.039
WBC (10^3/μL)	9.62±2.41	9.93±0.47	0.902
NEUT (10^3/μL)	2.29±0.87	1.55±0.04	0.421
LYMPH (10^3/μL)	7.86±2.06	7.95±0.43	0.969
MONO (10^3/μL)	0.31±0.13	0.19±0.05	0.370
EO (10^3/μL)	0.23±0.10	0.22±0.03	0.985
BASO (10^3/μL)	0.01±0.01	0.03±0.01	0.267

RBC, red blood cell count; HGB hemoglobin; HCT, hematocrit; MCV, mean corpuscular (erythrocyte) volume; MCH, mean corpuscular hemoglobin; MCHC, mean corpuscular hemoglobin concentration; RET, reticulocyte count; PLT, platelet count; WBC, white blood cells count; NEUT, neutrophil count; LYMPH, lymphocyte count; MONO, monocyte count; EO, eosinophil count; BASO, basophil count; Eu, euploid

Table S5. Indirect calorimetry analysis of male (25 weeks of age) Euploid and MAC21 littermate mice fed a high-fat diet (8.5 weeks on diet)

	**Eu**	**MAC21**	***P***	**Eu**	**MAC21**	***P***	**Eu**	**MAC21**	***P***
**High-fat diet (Male)**	***Ad-libitum* (dark cycle)**			***Ad-libitum* (light cycle)**			***Ad-libitum* (24 hr)**		
N	8	9		8	9		8	9	
Body weight (g)							**43.0 ± 1.99**	**25.8 ± 1.06**	**<0.001**
Food intake (kcal)	11.7 ± 1.02	12.6 ± 0.78	0.502	3.6 ± 0.61	3.6 ± 0.34	0.987	15.3 ± 1.20	16.2 ± 0.83	0.556
VO2 (mL/kg lean mass/h)	**4871 ± 107**	**6378 ± 316**	**<0.001**	**4046 ± 74**	**5222 ± 250**	**<0.001**	**4459 ± 84**	**5800 ± 281**	**<0.001**
VCO2 (mL/kg lean mass/h)	**3956 ± 88**	**5316 ± 255**	**<0.001**	**3270 ± 65**	**4274 ± 216**	**<0.001**	**3613 ± 68**	**4795 ± 232**	**<0.001**
RER (VCO2/VO2)	**0.81 ± 0.004**	**0.83 ± 0.003**	**0.001**	0.81 ± 0.004	0.82 ± 0.006	0.244	**0.81 ± 0.003**	**0.83 ± 0.004**	**0.011**
EE (kcal/kg lean mass/h)	**23.5 ± 0.51**	**30.9 ± 1.52**	**<0.001**	**19.5 ± 0.36**	**25.2 ± 1.22**	**<0.001**	**21.5 ± 0.40**	**28.0 ± 1.36**	**<0.001**
Total activity (beam breaks)	**22378 ± 1644**	**41249 ± 4280**	**0.001**	**8153 ± 1249**	**14099 ± 1570**	**0.011**	**30531 ± 2553**	**16508 ± 5503**	**0.001**
Ambulatory activity (counts)	**11114 ± 1669**	**20126 ± 2350**	**0.008**	3571 ± 1295	5798 ± 730	0.144	**14685 ± 2859**	**25924 ± 2898**	**0.015**
	**Fasting (dark cycle)**			**Fasting (light cycle)**			**Fasting (24 hr)**		
Body weight (g)							**40.3 ± 1.89**	**23.1 ± 1.06**	**<0.001**
Food intake (kcal)	0	0		0	0		0	0	
VO2 (mL/kg lean mass/h)	**4389 ± 138**	**5063 ± 238**	**0.028**	3475 ± 84	3832 ± 176	0.089	**3932 ± 108**	**4447 ± 201**	**0.040**
VCO2 (mL/kg lean mass/h)	**3317 ± 101**	**3887 ± 172**	**0.013**	2650 ± 64	2953 ± 131	0.057	**2984 ± 80**	**3419 ± 146**	**0.020**
RER (VCO2/VO2)	0.76 ± 0.002	0.77 ± 0.007	0.096	0.76 ± 0.003	0.77 ± 0.004	0.149	0.76 ± 0.002	0.77 ± 0.005	0.109
EE (kcal/kg lean mass/h)	**20.8 ± 0.65**	**24.1 ± 1.12**	**0.024**	16.5 ± 0.40	18.3 ± 0.83	0.081	**18.7 ± 0.51**	**21.2 ± 0.94**	**0.035**
Total activity (beam breaks)	**25504 ± 3251**	**47893 ± 5189**	**0.003**	**6259 ± 815**	**14298 ± 1499**	**<0.001**	**31763 ± 3793**	**62191 ± 6644**	**0.001**
Ambulatory activity (counts)	**13987 ± 1797**	**28435 ± 3571**	**0.003**	**2381 ± 392**	**7565 ± 1177**	**<0.001**	**16368 ± 1981**	**36000 ± 4676**	**0.002**
	**Re-feed (dark cycle)**			**Re-feed (light cycle)**			**Re-feed (24 hr)**		
Body weight (g)							**41.4 ± 1.93**	**25.5 ± 0.90**	**<0.001**
Food intake (kcal)	12.9 0.62	13.4 ± 0.38	0.524	2.90 ± 0.62	3.6 ± 0.34	0.353	**13.0 ± 0.87**	**17.7 ± 0.82**	**0.002**
VO2 (mL/kg lean mass/h)	**4907 ± 133**	**5707 ± 225**	**0.008**	**3981 ± 77**	**4829 ± 187**	**<0.001**	**4459 ± 100**	**5282 ± 203**	**0.003**
VCO2 (mL/kg lean mass/h)	**3916 ± 104**	**4812 ± 164**	**<0.001**	**3233 ± 65**	**4130 ± 162**	**<0.001**	**3586 ± 79**	**4482 ± 159**	**<0.001**
RER (VCO2/VO2)	**0.80 ± 0.005**	**0.84 ± 0.008**	**<0.001**	**0.81 ± 0.004**	**0.86 ± 0.011**	**0.002**	**0.80 ± 0.004**	**0.85 ± 0.009**	**<0.001**
EE (kcal/kg lean mass/h)	**23.5 ± 0.63**	**27.7 ± 1.06**	**0.005**	**19.2 ± 0.37**	**23.5 ± 0.90**	**<0.001**	**21.4 ± 0.48**	**25.7 ± 0.97**	**0.001**
Total activity (beam breaks)	**49750 ± 4033**	**65119 ± 6049**	**0.048**	**5118 ± 712**	**9820 ± 1222**	**0.005**	**27579 ± 2805**	**46524 ± 5236**	**0.007**
Ambulatory activity (counts)	27781 ± 4810	38270 ± 3910	0.100	**1618 ± 186**	**3424 ± 434**	**0.002**	**11970 ± 847**	**22377 ± 2708**	**0.003**

Eu: Euploid; VO2: rate of oxygen consumption; VCO2: rate of carbon dioxide production; RER: respiratory exchange ratio; EE: energy expenditure

bioRxiv preprint doi: https://doi.org/10.1101/2023.01.30.526183; this version posted January 31, 2023. The copyright holder for this preprint (which was not certified by peer review) is the author/funder, who has granted bioRxiv a license to display the preprint in perpetuity. It is made available under aCC-BY 4.0 International license.

Table S6

Gonadal White Adipose Tissue (HFD, 22°C)

Human genes expressed

Gene Symbol	Log(Fold Change)	Adj.p-Value	Gene Type	GencodeID	EntrezID
DSCR3	10.74	6.24E-07	protein_coding	ENSG00000157538.13	10311
MCM3AP	10.31	6.24E-07	protein_coding	ENSG00000160294.10	8888
TRAPPC10	10.92	6.24E-07	protein_coding	ENSG00000160218.12	7109
USP16	10.61	6.24E-07	protein_coding	ENSG00000156256.14	10600
PTTG1IP	12.07	6.24E-07	protein_coding	ENSG00000183255.11	754
PRMT2	11.14	6.24E-07	protein_coding	ENSG00000160310.16	3275
BRWD1	10.52	6.24E-07	protein_coding	ENSG00000185658.13	54014
IFNGR2	10.30	6.24E-07	protein_coding	ENSG00000159128.14	3460
PIGP	10.71	6.24E-07	protein_coding	ENSG00000185808.13	51227
HMGN1	10.55	6.24E-07	protein_coding	ENSG00000205581.10	3150
SUMO3	11.29	6.24E-07	protein_coding	ENSG00000184900.15	6612
C2CD2	11.70	6.24E-07	protein_coding	ENSG00000157617.16	25966
TTC3	12.50	6.24E-07	protein_coding	ENSG00000182670.13	7267
APP	13.12	6.24E-07	protein_coding	ENSG00000142192.20	351
MX2	10.87	6.24E-07	protein_coding	ENSG00000183486.12	4600
S100B	13.02	6.24E-07	protein_coding	ENSG00000160307.9	6285
HLCS	9.83	7.70E-07	protein_coding	ENSG00000159267.14	3141
CRYZL1	9.72	7.70E-07	protein_coding	ENSG00000205758.11	9946
BTG3	10.05	7.70E-07	protein_coding	ENSG00000154640.14	10950
ATP5J	11.35	7.70E-07	protein_coding	ENSG00000154723.12	522
IFNAR1	10.75	7.70E-07	protein_coding	ENSG00000142166.12	3454
TSPEAR-AS1	11.19	7.70E-07	antisense	ENSG00000235890.2	NA
GABPA	10.39	7.93E-07	protein_coding	ENSG00000154727.10	2551
PAXBP1	9.31	7.93E-07	protein_coding	ENSG00000159086.14	94104
ATP5O	9.47	7.93E-07	protein_coding	ENSG00000241837.6	539
RRP1B	10.07	8.08E-07	protein_coding	ENSG00000160208.12	23076
TMEM50B	9.68	8.08E-07	protein_coding	ENSG00000142188.16	757
CCT8	11.07	8.08E-07	protein_coding	ENSG00000156261.12	10694
PDXK	12.34	8.08E-07	protein_coding	ENSG00000160209.18	8566
DYRK1A	10.18	8.08E-07	protein_coding	ENSG00000157540.19	1859
BACE2	10.18	8.08E-07	protein_coding	ENSG00000182240.15	25825
SLC37A1	9.40	8.08E-07	protein_coding	ENSG00000160190.13	54020
TSPEAR-AS2	10.46	8.08E-07	antisense	ENSG00000182912.6	NA
SCAF4	9.65	8.34E-07	protein_coding	ENSG00000156304.14	57466
RUNX1	9.80	8.34E-07	protein_coding	ENSG00000159216.18	861
DIP2A	11.62	1.06E-06	protein_coding	ENSG00000160305.17	23181
UBE2G2	10.76	1.19E-06	protein_coding	ENSG00000184787.18	7327
CBR1	9.67	1.23E-06	protein_coding	ENSG00000159228.12	873
LINC00649	9.71	1.24E-06	antisense	ENSG00000237945.7	100506334
SYNJ1	9.77	1.25E-06	protein_coding	ENSG00000159082.17	8867
SETD4	8.93	1.25E-06	protein_coding	ENSG00000185917.13	54093
NDUFV3	10.48	1.25E-06	protein_coding	ENSG00000160194.17	4731
ETS2	9.78	1.25E-06	protein_coding	ENSG00000157557.11	2114
SON	12.02	1.25E-06	protein_coding	ENSG00000159140.18	6651
RWDD2B	8.94	1.43E-06	protein_coding	ENSG00000156253.6	10069
RRP1	10.20	1.61E-06	protein_coding	ENSG00000160214.12	8568
WRB	9.77	1.75E-06	protein_coding	ENSG00000182093.14	7485
URB1	9.90	1.76E-06	protein_coding	ENSG00000142207.6	9875
PSMG1	8.59	2.01E-06	protein_coding	ENSG00000183527.11	8624
PKNOX1	8.48	2.04E-06	protein_coding	ENSG00000160199.14	5316
PRDM15	8.20	2.06E-06	protein_coding	ENSG00000141956.13	63977
ZBTB21	10.33	2.15E-06	protein_coding	ENSG00000173276.13	49854
PFKL	9.27	2.31E-06	protein_coding	ENSG00000141959.16	5211
HSPA13	10.09	2.31E-06	protein_coding	ENSG00000155304.5	6782
IFNAR2	9.48	2.33E-06	protein_coding	ENSG00000159110.19	3455
N6AMT1	8.08	2.49E-06	protein_coding	ENSG00000156239.11	29104
CYYR1	8.86	2.49E-06	protein_coding	ENSG00000166265.11	116159
ADAMTS1	8.42	2.66E-06	protein_coding	ENSG00000154734.14	9510
IL10RB	9.62	2.74E-06	protein_coding	ENSG00000243646.9	3588
ITSN1	11.09	2.83E-06	protein_coding	ENSG00000205726.14	6453
AGPAT3	10.66	3.98E-06	protein_coding	ENSG00000160216.18	56894
SH3BGR	9.20	4.62E-06	protein_coding	ENSG00000185437.13	6450
SOD1	11.57	4.79E-06	protein_coding	ENSG00000142168.14	6647
CLIC6	11.05	4.81E-06	protein_coding	ENSG00000159212.12	54102
DONSON	7.83	5.33E-06	protein_coding	ENSG00000159147.17	29980
CH507-9B2.5	8.41	5.52E-06	protein_coding	ENSG00000275464.4	102724159
ITGB2-AS1	10.41	5.92E-06	antisense	ENSG00000227039.6	100505746
ABCG1	8.34	6.16E-06	protein_coding	ENSG00000160179.18	9619
MX1	9.88	9.40E-06	protein_coding	ENSG00000157601.13	4599
C21orf59	7.62	9.53E-06	protein_coding	ENSG00000159079.18	56683
LTN1	8.26	9.53E-06	protein_coding	ENSG00000198862.13	26046
AP001065.15	8.02	9.97E-06	lincRNA	ENSG00000228709.1	NA
MAP3K7CL	8.76	1.01E-05	protein_coding	ENSG00000156265.15	56911
TFF3	8.29	1.11E-05	protein_coding	ENSG00000160180.15	7033
MRPL39	8.29	1.12E-05	protein_coding	ENSG00000154719.13	54148
U2AF1	7.36	1.15E-05	protein_coding	ENSG00000160201.11	7307
CSTB	13.49	1.15E-05	protein_coding	ENSG00000160213.5	1476
JAM2	8.61	1.20E-05	protein_coding	ENSG00000154721.14	58494
TSPEAR	7.99	1.73E-05	protein_coding	ENSG00000175894.14	54084
ADARB1	7.52	1.84E-05	protein_coding	ENSG00000197381.15	104
MRPS6	9.88	1.84E-05	protein_coding	ENSG00000243927.5	64968
LRRC3	8.99	1.85E-05	protein_coding	ENSG00000160233.7	81543
AP000235.2	6.96	2.25E-05	lincRNA	ENSG00000226983.2	339622
EVA1C	7.49	2.28E-05	protein_coding	ENSG00000166979.12	59271
PAXBP1-AS1	7.03	2.49E-05	antisense	ENSG00000238197.5	100506215
LINC01547	8.45	2.49E-05	lincRNA	ENSG00000183250.11	84536
TRPM2	9.51	2.52E-05	protein_coding	ENSG00000142185.16	7226
MIS18A	7.08	2.59E-05	protein_coding	ENSG00000159055.3	54069
CXADR	7.87	2.88E-05	protein_coding	ENSG00000154639.18	1525
BACH1	10.86	3.05E-05	protein_coding	ENSG00000156273.15	571
POFUT2	10.87	3.22E-05	protein_coding	ENSG00000186866.16	23275
CH507-9B2.3	7.95	3.31E-05	protein_coding	ENSG00000280071.3	102724023
RCAN1	9.20	3.31E-05	protein_coding	ENSG00000159200.17	1827
AATBC	8.52	3.54E-05	antisense	ENSG00000215458.8	284837
AIRE	6.54	3.60E-05	protein_coding	ENSG00000160224.16	326
SLC5A3	11.26	3.73E-05	protein_coding	ENSG00000198743.6	6526
DNAJC28	6.64	3.79E-05	protein_coding	ENSG00000177692.11	54943
C21orf33	8.07	5.04E-05	protein_coding	ENSG00000160221.16	8209
CHAF1B	9.77	5.58E-05	protein_coding	ENSG00000159259.7	8208
C21orf2	6.81	5.77E-05	protein_coding	ENSG00000160226.15	755
ANKRD30BP2	7.08	5.77E-05	transcribed_unprocessed_pseudogene	ENSG00000224309.7	NA
KCNJ15	7.41	5.77E-05	protein_coding	ENSG00000157551.17	3772
GART	9.42	5.82E-05	protein_coding	ENSG00000159131.16	2618
MRAP	6.45	6.31E-05	protein_coding	ENSG00000170262.12	56246
PCNT	9.44	6.57E-05	protein_coding	ENSG00000160299.16	5116
DSCR8	6.34	6.63E-05	protein_coding	ENSG00000198054.11	84677
HUNK	6.50	7.11E-05	protein_coding	ENSG00000142149.8	30811
CH507-9B2.9	6.27	7.83E-05	protein_coding	ENSG00000280433.1	102724200
LCA5L	6.61	7.98E-05	protein_coding	ENSG00000157578.13	150082
LINC01436	6.51	7.98E-05	lincRNA	ENSG00000231106.2	NA
WDR4	6.88	8.44E-05	protein_coding	ENSG00000160193.11	10785
TRPM2-AS	6.00	9.22E-05	antisense	ENSG00000230061.2	NA
URB1-AS1	6.63	1.47E-04	lincRNA	ENSG00000256073.3	84996
AF015262.2	6.60	1.47E-04	lincRNA	ENSG00000234703.1	NA
TIAM1	8.88	1.85E-04	protein_coding	ENSG00000156299.13	7074
HSF2BP	6.05	2.00E-04	protein_coding	ENSG00000160207.8	11077
BX322557.10	6.29	2.21E-04	processed_transcript	ENSG00000215447.7	NA
ITGB2	14.12	2.52E-04	protein_coding	ENSG00000160255.17	3689
FAM207A	8.51	2.66E-04	protein_coding	ENSG00000160256.12	85395
RPL23P2	6.74	2.85E-04	processed_pseudogene	ENSG00000176054.6	NA
UBASH3A	6.51	3.56E-04	protein_coding	ENSG00000160185.14	53347
LINC00205	5.54	6.66E-04	lincRNA	ENSG00000223768.1	NA
LINC01426	6.95	7.00E-04	antisense	ENSG00000234380.1	100506385
DSCR4	5.72	7.48E-04	protein_coding	ENSG00000184029.9	10281
BRWD1-AS2	5.16	7.67E-04	antisense	ENSG00000255568.3	103091865
SAMSN1	11.44	7.67E-04	protein_coding	ENSG00000155307.17	64092
DSTNP1	6.75	1.10E-03	processed_pseudogene	ENSG00000230982.1	NA
CBSL	5.37	1.10E-03	protein_coding	ENSG00000274276.4	102724560
C21orf58	7.65	1.18E-03	protein_coding	ENSG00000160298.17	54058
ANKRD30BP1	7.72	1.43E-03	unprocessed_pseudogene	ENSG00000175302.5	NA
21NC02-1C16	6.06	1.80E-03	antisense	ENSG00000272825.1	NA
LINC00479	5.07	3.25E-03	lincRNA	ENSG00000236384.7	150135
AF129075.5	5.93	3.40E-03	sense_intronic	ENSG00000231125.2	NA
FAM3B	5.17	3.85E-03	protein_coding	ENSG00000183844.16	54097
C21orf62	4.91	4.17E-03	protein_coding	ENSG00000205929.10	56245
AP001505.10	4.58	4.85E-03	lincRNA	ENSG00000276529.1	NA
TMPRSS3	5.24	5.16E-03	protein_coding	ENSG00000160183.13	64699
CBR3	5.57	5.16E-03	protein_coding	ENSG00000159231.5	874
RSPH1	6.75	5.16E-03	protein_coding	ENSG00000160188.9	89765
AP001628.7	4.74	6.12E-03	lincRNA	ENSG00000233754.2	NA
AP001432.14	5.26	7.95E-03	lincRNA	ENSG00000242553.1	NA
KCNE2	4.58	8.49E-03	protein_coding	ENSG00000159197.3	9992
YBEY	4.81	8.74E-03	protein_coding	ENSG00000182362.13	54059
LINC00112	4.10	9.20E-03	antisense	ENSG00000232401.1	54089
SNORA81	4.16	1.23E-02	snoRNA	ENSG00000238390.1	NA
AP000266.7	4.43	1.28E-02	antisense	ENSG00000232623.1	NA
AP001052.1	4.10	1.31E-02	pseudogene	ENSG00000281420.1	NA
AP001627.1	4.14	1.51E-02	antisense	ENSG00000225731.1	NA
ZNF295-AS1	4.23	2.39E-02	lincRNA	ENSG00000237232.7	150142
POTED	4.39	2.39E-02	protein_coding	ENSG00000166351.10	317754
AP000254.8	4.08	3.03E-02	antisense	ENSG00000273271.1	NA
KB-68A7.2	4.13	3.63E-02	lincRNA	ENSG00000277352.1	NA
LINC00189	5.51	3.87E-02	sense_overlapping	ENSG00000215533.8	193629
AP001625.4	4.15	4.06E-02	sense_intronic	ENSG00000239930.2	101930094

Mouse up-regulated genes

Gene Symbol	Log(Fold Change)	Adj.p-Value	Gene Type	GencodeID	EntrezID
Orm3	3.68	3.12E-02	protein_coding	ENSMUSG00000028359.4	18407
Zfp970	1.44	4.62E-02	protein_coding	ENSMUSG00000078866.10	628308
Chst11	0.99	4.89E-02	protein_coding	ENSMUSG00000034612.7	58250

Mouse down-regulated genes

Gene Symbol	Log(Fold Change)	Adj.p-Value	Gene Type	GencodeID	EntrezID
Sgcg	-11.75	2.27E-02	protein_coding	ENSMUSG00000035296.13	24053
Mcpt2	-11.17	2.33E-02	protein_coding	ENSMUSG00000022226.5	17225
Igkv19-93	-8.37	1.67E-02	IG_V_gene	ENSMUSG00000098814.2	NA
Sult4a1	-6.01	7.08E-03	protein_coding	ENSMUSG00000018865.8	29859
Gm3375	-5.67	4.69E-03	processed_pseudogene	ENSMUSG00000107470.1	NA
Pex5l	-4.85	1.35E-02	protein_coding	ENSMUSG00000027674.16	58869
Cpz	-2.73	2.70E-02	protein_coding	ENSMUSG00000036596.6	242939
Col6a5	-1.84	4.47E-02	protein_coding	ENSMUSG00000091345.8	665033

bioRxiv preprint doi: https://doi.org/10.1101/2023.01.30.526183; this version posted January 31, 2023. The copyright holder for this preprint (which was not certified by peer review) is the author/funder, who has granted bioRxiv a license to display the preprint in perpetuity. It is made available under aCC-BY 4.0 International license.

Table S7

Inguinal White Adipose Tissue (HFD, 22°C)

Human genes expressed

Gene Symbol	Log(Fold Change)	Adj.p-Value	Gene Type	GencodeID	EntrezID
ATP5O	12.02	0.014	protein_coding	ENSG00000241837.6	539
NDUFV3	11.81	0.021	protein_coding	ENSG00000160194.17	4731
AGPAT3	11.50	0.003	protein_coding	ENSG00000160216.18	56894
ATP5J	11.47	0.021	protein_coding	ENSG00000154723.12	522
S100B	11.44	0.022	protein_coding	ENSG00000160307.9	6285
TRAPPC10	11.08	0.003	protein_coding	ENSG00000160218.12	7109
UBE2G2	10.53	0.015	protein_coding	ENSG00000184787.18	7327
RRP1	10.35	0.014	protein_coding	ENSG00000160214.12	8568
MCM3AP	10.33	0.014	protein_coding	ENSG00000160294.10	8888
HMGN1	10.28	0.024	protein_coding	ENSG00000205581.10	3150
IL10RB	10.06	0.017	protein_coding	ENSG00000243646.9	3588
GABPA	9.82	0.017	protein_coding	ENSG00000154727.10	2551
PAXBP1	9.80	0.014	protein_coding	ENSG00000159086.14	94104
CBR1	9.77	0.014	protein_coding	ENSG00000159228.12	873
HLCS	9.40	0.007	protein_coding	ENSG00000159267.14	3141
CRYZL1	9.10	0.014	protein_coding	ENSG00000205758.11	9946
USP16	9.09	0.037	protein_coding	ENSG00000156256.14	10600
URB1	9.07	0.018	protein_coding	ENSG00000142207.6	9875
BRWD1	8.78	0.017	protein_coding	ENSG00000185658.13	54014
CH507-9B2.5	8.60	0.014	protein_coding	ENSG00000275464.4	102724159
SH3BGR	8.48	0.018	protein_coding	ENSG00000185437.13	6450
LRRC3	8.25	0.030	protein_coding	ENSG00000160233.7	81543
SETD4	8.17	0.017	protein_coding	ENSG00000185917.13	54093
PSMG1	8.06	0.014	protein_coding	ENSG00000183527.11	8624
PKNOX1	8.01	0.038	protein_coding	ENSG00000160199.14	5316
MRPL39	7.72	0.017	protein_coding	ENSG00000154719.13	54148
ADARB1	7.27	0.034	protein_coding	ENSG00000197381.15	104

Mouse up-regulated genes

Gene Symbol	Log(Fold Change)	Adj.p-Value	Gene Type	GencodeID	EntrezID
NONE					

Mouse down-regulated genes

Gene Symbol	Log(Fold Change)	Adj.p-Value	Gene Type	GencodeID	EntrezID
Krt5	-19.19	0.046	protein_coding	ENSMUSG00000061527.6	110308
Dsp	-16.95	0.014	protein_coding	ENSMUSG00000054889.9	109620
Ano9	-11.97	0.027	protein_coding	ENSMUSG00000054662.8	71345
Rarres1	-6.35	0.030	protein_coding	ENSMUSG00000049404.7	109222
Tmem132b	-5.93	0.014	protein_coding	ENSMUSG00000070498.3	208151
Grid1	-4.89	0.018	protein_coding	ENSMUSG00000041078.6	14803

bioRxiv preprint doi: https://doi.org/10.1101/2023.01.30.526183; this version posted January 31, 2023. The copyright holder for this preprint (which was not certified by peer review) is the author/funder, who has granted bioRxiv a license to display the preprint in perpetuity. It is made available under aCC-BY 4.0 International license.

Table S8 Liver (HFD, 22°C)

Human genes expressed

Gene Symbol	Log(Fold Change)	Adj.p-Value	Gene Type	GencodeID	EntrezID
SOD1	12.78	7.85E-07	protein_coding	ENSG00000142168.14	6647
MX1	11.52	2.61E-05	protein_coding	ENSG00000157601.13	4599
APP	11.48	7.85E-07	protein_coding	ENSG00000142192.20	351
PDXK	11.13	5.31E-06	protein_coding	ENSG00000160209.18	8566
PTTG1IP	10.80	9.91E-07	protein_coding	ENSG00000183255.11	754
SON	10.76	7.23E-06	protein_coding	ENSG00000159140.18	6651
CBR1	10.65	1.44E-05	protein_coding	ENSG00000159228.12	873
ATP5J	10.59	1.61E-06	protein_coding	ENSG00000154723.12	522
AGPAT3	10.51	4.02E-05	protein_coding	ENSG00000160216.18	56894
CSTB	10.14	2.54E-06	protein_coding	ENSG00000160213.5	1476
POFUT2	10.14	1.47E-05	protein_coding	ENSG00000186866.16	23275
DSCR3	10.11	1.07E-06	protein_coding	ENSG00000157538.13	10311
PRMT2	10.08	4.17E-06	protein_coding	ENSG00000160310.16	3275
UBE2G2	10.06	2.16E-06	protein_coding	ENSG00000184787.18	7327
DIP2A	10.00	5.84E-05	protein_coding	ENSG00000160305.17	23181
NDUFV3	9.92	6.07E-06	protein_coding	ENSG00000160194.17	4731
SUMO3	9.88	1.95E-06	protein_coding	ENSG00000184900.15	6612
BACH1	9.83	2.53E-05	protein_coding	ENSG00000156273.15	571
TRAPPC10	9.83	2.54E-06	protein_coding	ENSG00000160218.12	7109
WRB	9.82	1.25E-06	protein_coding	ENSG00000182093.14	7485
HMGN1	9.79	7.00E-06	protein_coding	ENSG00000205581.10	3150
CCT8	9.77	2.16E-06	protein_coding	ENSG00000156261.12	10694
ETS2	9.74	2.69E-04	protein_coding	ENSG00000157557.11	2114
C21orf91	9.73	2.36E-05	protein_coding	ENSG00000154642.10	54149
CBS	9.73	2.87E-06	protein_coding	ENSG00000160200.17	875
BRWD1	9.66	1.44E-05	protein_coding	ENSG00000185658.13	54014
IFNAR1	9.65	6.01E-06	protein_coding	ENSG00000142166.12	3454
TTC3	9.53	1.15E-05	protein_coding	ENSG00000182670.13	7267
GART	9.46	2.54E-06	protein_coding	ENSG00000159131.16	2618
AATBC	9.46	6.93E-04	antisense	ENSG00000215458.8	284837
DYRK1A	9.40	2.16E-06	protein_coding	ENSG00000157540.19	1859
ITGB2	9.38	1.74E-04	protein_coding	ENSG00000160255.17	3689
USP16	9.26	9.29E-06	protein_coding	ENSG00000156256.14	10600
PFKL	9.20	2.54E-05	protein_coding	ENSG00000141959.16	5211
CXADR	9.07	2.16E-06	protein_coding	ENSG00000154639.18	1525
MCM3AP	9.01	2.16E-06	protein_coding	ENSG00000160294.10	8888
ITSN1	8.98	2.16E-06	protein_coding	ENSG00000205726.14	6453
GABPA	8.89	7.99E-06	protein_coding	ENSG00000154727.10	2551
PIGP	8.86	2.16E-06	protein_coding	ENSG00000185808.13	51227
SCAF4	8.85	2.41E-04	protein_coding	ENSG00000156304.14	57466
TMEM50B	8.85	3.94E-06	protein_coding	ENSG00000142188.16	757
RRP1	8.84	1.34E-05	protein_coding	ENSG00000160214.12	8568
RRP1B	8.80	2.27E-05	protein_coding	ENSG00000160208.12	23076
HLCS	8.75	3.53E-04	protein_coding	ENSG00000159267.14	3141
LRRC3	8.72	2.63E-03	protein_coding	ENSG00000160233.7	81543
TSPEAR-AS2	8.71	9.97E-06	antisense	ENSG00000182912.6	NA
URB1	8.70	2.54E-06	protein_coding	ENSG00000142207.6	9875
BACE2	8.64	1.47E-05	protein_coding	ENSG00000182240.15	25825
RCAN1	8.60	4.02E-05	protein_coding	ENSG00000159200.17	1827
TSPEAR-AS1	8.59	6.07E-06	antisense	ENSG00000235890.2	NA
IFNGR2	8.57	2.36E-05	protein_coding	ENSG00000159128.14	3460
MX2	8.54	1.77E-04	protein_coding	ENSG00000183486.12	4600
ATP5O	8.46	9.29E-06	protein_coding	ENSG00000241837.6	539
TMPRSS2	8.40	1.47E-05	protein_coding	ENSG00000184012.11	7113
RUNX1	8.36	2.64E-05	protein_coding	ENSG00000159216.18	861
TMPRSS3	8.31	1.71E-03	protein_coding	ENSG00000160183.13	64699
SETD4	8.31	3.80E-06	protein_coding	ENSG00000185917.13	54093
PSMG1	8.31	3.69E-06	protein_coding	ENSG00000183527.11	8624
RIPK4	8.30	7.72E-05	protein_coding	ENSG00000183421.11	54101
CH507-9B2.3	8.19	1.47E-05	protein_coding	ENSG00000280071.3	102724023
CLIC6	8.18	3.20E-03	protein_coding	ENSG00000159212.12	54102
RWDD2B	8.13	9.29E-06	protein_coding	ENSG00000156253.6	10069
PRDM15	8.12	3.94E-06	protein_coding	ENSG00000141956.13	63977
IFNAR2	8.07	4.98E-05	protein_coding	ENSG00000159110.19	3455
SLC37A1	8.07	1.47E-05	protein_coding	ENSG00000160190.13	54020
SLC5A3	8.05	2.41E-04	protein_coding	ENSG00000198743.6	6526
PAXBP1	8.01	8.78E-06	protein_coding	ENSG00000159086.14	94104
SYNJ1	7.99	1.44E-05	protein_coding	ENSG00000159082.17	8867
FAM207A	7.99	6.53E-05	protein_coding	ENSG00000160256.12	85395
CRYZL1	7.94	7.23E-06	protein_coding	ENSG00000205758.11	9946
C2CD2	7.92	1.27E-05	protein_coding	ENSG00000157617.16	25966
IL10RB	7.87	8.65E-06	protein_coding	ENSG00000243646.9	3588
BTG3	7.82	7.72E-05	protein_coding	ENSG00000154640.14	10950
SH3BGR	7.75	6.07E-06	protein_coding	ENSG00000185437.13	6450
DONSON	7.71	7.80E-05	protein_coding	ENSG00000159147.17	29980
TRPM2	7.70	1.44E-05	protein_coding	ENSG00000142185.16	7226
ZBTB21	7.65	2.23E-05	protein_coding	ENSG00000173276.13	49854
CBSL	7.52	2.71E-03	protein_coding	ENSG00000274276.4	102724560
MRPL39	7.52	7.30E-05	protein_coding	ENSG00000154719.13	54148
LINC00649	7.46	7.30E-05	antisense	ENSG00000237945.7	100506334
HSPA13	7.42	1.53E-04	protein_coding	ENSG00000155304.5	6782
PCNT	7.36	3.30E-05	protein_coding	ENSG00000160299.16	5116
HUNK	7.36	3.27E-05	protein_coding	ENSG00000142149.8	30811
N6AMT1	7.35	7.30E-05	protein_coding	ENSG00000156239.11	29104
CH507-9B2.5	7.31	6.86E-05	protein_coding	ENSG00000275464.4	102724159
MAP3K7CL	7.28	1.74E-04	protein_coding	ENSG00000156265.15	56911
CLDN14	7.15	2.95E-03	protein_coding	ENSG00000159261.10	23562
PKNOX1	7.14	1.76E-05	protein_coding	ENSG00000160199.14	5316
C21orf33	7.12	7.30E-05	protein_coding	ENSG00000160221.16	8209
SAMSN1	7.01	1.37E-02	protein_coding	ENSG00000155307.17	64092
AP001065.15	6.93	1.59E-04	lincRNA	ENSG00000228709.1	NA
WDR4	6.89	2.69E-04	protein_coding	ENSG00000160193.11	10785
MRPS6	6.87	3.13E-04	protein_coding	ENSG00000243927.5	64968
C21orf58	6.86	6.53E-05	protein_coding	ENSG00000160298.17	54058
S100B	6.80	9.01E-03	protein_coding	ENSG00000160307.9	6285
LINC00479	6.78	4.60E-02	lincRNA	ENSG00000236384.7	150135
ITGB2-AS1	6.73	1.09E-03	antisense	ENSG00000227039.6	100505746
YBEY	6.54	1.10E-04	protein_coding	ENSG00000182362.13	54059
MIS18A	6.44	4.11E-04	protein_coding	ENSG00000159055.3	54069
URB1-AS1	6.41	2.63E-03	lincRNA	ENSG00000256073.3	84996
DNAJC28	6.36	1.74E-04	protein_coding	ENSG00000177692.11	54943
LTN1	6.22	2.70E-04	protein_coding	ENSG00000198862.13	26046
C21orf59	6.21	1.33E-04	protein_coding	ENSG00000159079.18	56683
TSPEAR	6.06	1.91E-02	protein_coding	ENSG00000175894.14	54084
TRPM2-AS	5.96	1.27E-03	antisense	ENSG00000230061.2	NA
DSCR8	5.95	2.41E-04	protein_coding	ENSG00000198054.11	84677
U2AF1	5.93	2.27E-03	protein_coding	ENSG00000160201.11	7307
TFF3	5.91	4.05E-03	protein_coding	ENSG00000160180.15	7033
DSCR4	5.80	1.13E-03	protein_coding	ENSG00000184029.9	10281
CYYR1	5.80	4.62E-04	protein_coding	ENSG00000166265.11	116159
JAM2	5.49	9.67E-03	protein_coding	ENSG00000154721.14	58494
ADARB1	5.47	4.66E-04	protein_coding	ENSG00000197381.15	104
LINC01426	5.44	2.99E-02	antisense	ENSG00000234380.1	100506385
LCA5L	5.44	6.96E-03	protein_coding	ENSG00000157578.13	150082
RPL23P2	5.34	1.75E-03	processed_pseudogene	ENSG00000176054.6	NA
KCNJ15	5.26	4.96E-02	protein_coding	ENSG00000157551.17	3772
SSR4P1	5.24	1.12E-03	transcribed_processed_pseudogene	ENSG00000235374.2	728039
LINC01547	5.08	1.13E-03	lincRNA	ENSG00000183250.11	84536
C21orf91-OT1	5.07	1.70E-03	lincRNA	ENSG00000240770.5	246312
RPL39P40	5.07	2.71E-03	processed_pseudogene	ENSG00000226580.1	NA
ANKRD30BP2	4.97	1.56E-02	transcribed_unprocessed_pseudogene	ENSG00000224309.7	NA
BRWD1-AS2	4.90	1.05E-02	antisense	ENSG00000255568.3	103091865
CHAF1B	4.75	4.98E-02	protein_coding	ENSG00000159259.7	8208
TIAM1	4.72	3.17E-03	protein_coding	ENSG00000156299.13	7074
LINC00189	4.70	3.34E-02	sense_overlapping	ENSG00000215533.8	193629
C21orf2	4.70	5.70E-03	protein_coding	ENSG00000160226.15	755
ABCG1	4.60	1.20E-02	protein_coding	ENSG00000160179.18	9619
DSTNP1	4.29	1.81E-02	processed_pseudogene	ENSG00000230982.1	NA
PAXBP1-AS1	4.21	1.75E-02	antisense	ENSG00000238197.5	100506215
LINC00205	4.16	8.46E-03	lincRNA	ENSG00000223768.1	NA
CH507-9B2.1	4.01	1.62E-02	protein_coding	ENSG00000277117.4	102723996
AP000302.58	3.73	4.09E-02	antisense	ENSG00000231355.1	NA
LINC01436	3.69	4.35E-02	lincRNA	ENSG00000231106.2	NA
SNORA81	3.64	4.39E-02	snoRNA	ENSG00000238390.1	NA

Mouse up-regulated genes

Gene Symbol	Log(Fold Change)	Adj.p-Value	Gene Type	GencodeID	EntrezID
Mirg	8.40	0.016	lincRNA	ENSMUSG00000097391.8	NA
Lrtm2	7.46	0.022	protein_coding	ENSMUSG00000055003.14	211187
Ehf	7.03	0.017	protein_coding	ENSMUSG00000012350.15	13661
Rab25	5.71	0.001	protein_coding	ENSMUSG00000008601.12	53868
BC067074	5.66	0.015	protein_coding	ENSMUSG00000021763.16	408066
Gm11454	5.18	0.050	lincRNA	ENSMUSG00000086152.1	NA
Muc13	5.13	0.015	protein_coding	ENSMUSG00000022824.12	17063
Ccdc198	5.08	0.050	protein_coding	ENSMUSG00000021850.13	67082
Rasal1	4.96	0.037	protein_coding	ENSMUSG00000029602.11	19415
Zfp365	4.65	0.017	protein_coding	ENSMUSG00000037855.15	216049
Tubb4a	4.58	0.009	protein_coding	ENSMUSG00000062591.5	22153
Ncam1	4.15	0.037	protein_coding	ENSMUSG00000039542.15	17967
Dpf1	3.97	0.009	protein_coding	ENSMUSG00000030584.14	29861
Oip5	3.89	0.049	protein_coding	ENSMUSG00000072980.3	70645
Bhlha15	3.86	0.041	protein_coding	ENSMUSG00000052271.7	17341
Cxcl14	3.45	0.027	protein_coding	ENSMUSG00000021508.10	57266
Nucb2	3.41	0.016	protein_coding	ENSMUSG00000030659.14	53322
Zfp641	3.06	0.050	protein_coding	ENSMUSG00000022987.11	239652
Catsperd	2.95	0.042	protein_coding	ENSMUSG00000040828.9	106757
Tnfrsf23	2.69	0.033	protein_coding	ENSMUSG00000037613.16	79201
Oas2	2.65	0.047	protein_coding	ENSMUSG00000032690.16	246728
Cttnbp2nl	1.75	0.038	protein_coding	ENSMUSG00000062127.11	80281
Dusp10	1.74	0.022	protein_coding	ENSMUSG00000039384.8	63953
Fat1	1.52	0.047	protein_coding	ENSMUSG00000070047.13	14107
Alcam	0.92	0.037	protein_coding	ENSMUSG00000022636.13	11658

Mouse down-regulated genes

Gene Symbol	Log(Fold Change)	Adj.p-Value	Gene Type	GencodeID	EntrezID
Acot3	-2.94	0.037	protein_coding	ENSMUSG00000021228.14	171281
Gm42796	-2.77	0.037	TEC	ENSMUSG00000105192.1	NA
Crym	-2.34	0.030	protein_coding	ENSMUSG00000030905.5	12971
Tmem256	-1.58	0.020	protein_coding	ENSMUSG00000070394.10	69186
Impg2	-1.39	0.037	protein_coding	ENSMUSG00000035270.15	224224
Hint2	-1.34	0.012	protein_coding	ENSMUSG00000028470.10	68917
Ttc36	-1.31	0.047	protein_coding	ENSMUSG00000039438.6	192653
Cox7c	-1.10	0.047	protein_coding	ENSMUSG00000017778.14	12867
Slc40a1	-1.04	0.047	protein_coding	ENSMUSG00000025993.10	53945

bioRxiv preprint doi: https://doi.org/10.1101/2023.01.30.526183; this version posted January 31, 2023. The copyright holder for this preprint (which was not certified by peer review) is the author/funder, who has granted bioRxiv a license to display the preprint in perpetuity. It is made available under aCC-BY 4.0 International license.

Table S9

Brown Adipose Tissue (HFD, 22°C)

Human genes expressed

Gene Symbol	Log(Fold Change)	Adj.p-Value	Gene Type	GencodeID	EntrezID
PDXK	11.14	4.20E-05	protein_coding	ENSG00000160209.18	8566
AATBC	10.77	4.20E-05	antisense	ENSG00000215458.8	284837
SLC5A3	10.17	2.12E-06	protein_coding	ENSG00000198743.6	6526
NDUFV3	10.16	4.06E-05	protein_coding	ENSG00000160194.17	4731
ATP5O	10.01	6.31E-05	protein_coding	ENSG00000241837.6	539
CLIC6	9.89	3.42E-06	protein_coding	ENSG00000159212.12	54102
ATP5J	9.85	1.42E-04	protein_coding	ENSG00000154723.12	522
CSTB	9.82	1.94E-05	protein_coding	ENSG00000160213.5	1476
S100B	9.76	6.31E-05	protein_coding	ENSG00000160307.9	6285
SH3BGR	9.71	1.08E-04	protein_coding	ENSG00000185437.13	6450
ANKRD20A11P	9.61	2.49E-05	transcribed_unprocessed_pseudogene	ENSG00000215559.8	391267
PTTG1IP	9.60	7.18E-06	protein_coding	ENSG00000183255.11	754
CCT8	9.55	4.09E-05	protein_coding	ENSG00000156261.12	10694
DSCR3	9.54	1.98E-06	protein_coding	ENSG00000157538.13	10311
C2CD2	9.53	1.86E-06	protein_coding	ENSG00000157617.16	25966
WRB	9.44	8.11E-05	protein_coding	ENSG00000182093.14	7485
CH507-9B2.3	9.36	7.56E-05	protein_coding	ENSG00000280071.3	102724023
POFUT2	9.21	5.99E-04	protein_coding	ENSG00000186866.16	23275
ITGB2	9.07	1.20E-05	protein_coding	ENSG00000160255.17	3689
TRAPPC10	9.04	3.05E-05	protein_coding	ENSG00000160218.12	7109
CYP4F29P	8.92	1.95E-05	transcribed_unprocessed_pseudogene	ENSG00000228314.1	54055
USP16	8.87	4.20E-05	protein_coding	ENSG00000156256.14	10600
MCM3AP	8.79	1.32E-05	protein_coding	ENSG00000160294.10	8888
PRMT2	8.77	5.89E-05	protein_coding	ENSG00000160310.16	3275
UBE2G2	8.76	6.19E-04	protein_coding	ENSG00000184787.18	7327
MX2	8.74	2.73E-05	protein_coding	ENSG00000183486.12	4600
DIP2A	8.69	7.88E-05	protein_coding	ENSG00000160305.17	23181
PIGP	8.67	2.49E-05	protein_coding	ENSG00000185808.13	51227
SON	8.66	3.69E-04	protein_coding	ENSG00000159140.18	6651
GART	8.64	4.09E-05	protein_coding	ENSG00000159131.16	2618
C21orf33	8.63	7.24E-04	protein_coding	ENSG00000160221.16	8209
URB1	8.60	6.83E-05	protein_coding	ENSG00000142207.6	9875
BRWD1	8.56	3.58E-04	protein_coding	ENSG00000185658.13	54014
URB1-AS1	8.54	4.00E-05	lincRNA	ENSG00000256073.3	84996
RWDD2B	8.54	2.12E-06	protein_coding	ENSG00000156253.6	10069
MAP3K7CL	8.53	1.85E-04	protein_coding	ENSG00000156265.15	56911
GABPA	8.49	2.12E-06	protein_coding	ENSG00000154727.10	2551
RRP1B	8.47	2.73E-05	protein_coding	ENSG00000160208.12	23076
DYRK1A	8.44	8.94E-05	protein_coding	ENSG00000157540.19	1859
APP	8.43	8.35E-04	protein_coding	ENSG00000142192.20	351
AGPAT3	8.39	8.91E-04	protein_coding	ENSG00000160216.18	56894
HMGN1	8.36	1.53E-04	protein_coding	ENSG00000205581.10	3150
RRP1	8.35	1.02E-05	protein_coding	ENSG00000160214.12	8568
IFNAR1	8.35	5.89E-05	protein_coding	ENSG00000142166.12	3454
IFNGR2	8.34	2.12E-06	protein_coding	ENSG00000159128.14	3460
TTC3	8.34	1.24E-04	protein_coding	ENSG00000182670.13	7267
LINC00649	8.29	3.42E-06	antisense	ENSG00000237945.7	100506334
BTG3	8.27	2.61E-05	protein_coding	ENSG00000154640.14	10950
ITSN1	8.26	6.09E-05	protein_coding	ENSG00000205726.14	6453
ZBTB21	8.21	2.58E-06	protein_coding	ENSG00000173276.13	49854
CH507-9B2.5	8.20	2.12E-06	protein_coding	ENSG00000275464.4	102724159
TMEM50B	8.17	3.05E-05	protein_coding	ENSG00000142188.16	757
BACE2	8.08	2.12E-06	protein_coding	ENSG00000182240.15	25825
MRPL39	8.03	7.56E-05	protein_coding	ENSG00000154719.13	54148
HLCS	7.98	1.98E-04	protein_coding	ENSG00000159267.14	3141
CRYZL1	7.90	1.94E-04	protein_coding	ENSG00000205758.11	9946
LTN1	7.89	2.12E-06	protein_coding	ENSG00000198862.13	26046
SUMO3	7.89	2.41E-04	protein_coding	ENSG00000184900.15	6612
TSPEAR-AS1	7.87	5.08E-05	antisense	ENSG00000235890.2	NA
N6AMT1	7.85	5.08E-05	protein_coding	ENSG00000156239.11	29104
MRPS6	7.76	7.88E-05	protein_coding	ENSG00000243927.5	64968
CYYR1	7.75	7.76E-05	protein_coding	ENSG00000166265.11	116159
TSPEAR-AS2	7.74	7.51E-06	antisense	ENSG00000182912.6	NA
CBR1	7.73	8.00E-04	protein_coding	ENSG00000159228.12	873
SYNJ1	7.73	2.12E-06	protein_coding	ENSG00000159082.17	8867
PFKL	7.50	8.00E-04	protein_coding	ENSG00000141959.16	5211
PCNT	7.49	3.81E-05	protein_coding	ENSG00000160299.16	5116
PAXBP1	7.49	5.95E-05	protein_coding	ENSG00000159086.14	94104
ETS2	7.47	1.98E-04	protein_coding	ENSG00000157557.11	2114
HSPA13	7.47	3.42E-06	protein_coding	ENSG00000155304.5	6782
MX1	7.37	8.72E-03	protein_coding	ENSG00000157601.13	4599
SETD4	7.34	3.58E-04	protein_coding	ENSG00000185917.13	54093
FAM207A	7.22	4.09E-05	protein_coding	ENSG00000160256.12	85395
PSMG1	7.22	3.40E-05	protein_coding	ENSG00000183527.11	8624
IFNAR2	7.21	2.12E-06	protein_coding	ENSG00000159110.19	3455
DNAJC28	7.17	2.61E-05	protein_coding	ENSG00000177692.11	54943
JAM2	7.07	1.00E-03	protein_coding	ENSG00000154721.14	58494
SNX18P13	7.01	3.40E-05	processed_pseudogene	ENSG00000230965.1	NA
WDR4	6.98	3.42E-06	protein_coding	ENSG00000160193.11	10785
ITGB2-AS1	6.98	3.21E-05	antisense	ENSG00000227039.6	100505746
PRDM15	6.97	9.83E-04	protein_coding	ENSG00000141956.13	63977
SLC37A1	6.93	3.35E-05	protein_coding	ENSG00000160190.13	54020
C21orf58	6.89	7.23E-04	protein_coding	ENSG00000160298.17	54058
PKNOX1	6.87	2.73E-05	protein_coding	ENSG00000160199.14	5316
RUNX1	6.87	3.01E-04	protein_coding	ENSG00000159216.18	861
SCAF4	6.87	1.38E-03	protein_coding	ENSG00000156304.14	57466
SOD1	6.83	4.65E-03	protein_coding	ENSG00000142168.14	6647
RHOT1P2	6.80	2.95E-05	processed_pseudogene	ENSG00000203616.2	NA
MIS18A	6.76	1.36E-04	protein_coding	ENSG00000159055.3	54069
AP000235.2	6.58	1.33E-04	lincRNA	ENSG00000226983.2	339622
TRPM2	6.58	7.53E-05	protein_coding	ENSG00000142185.16	7226
BACH1	6.47	2.59E-04	protein_coding	ENSG00000156273.15	571
BRWD1-AS2	6.39	3.56E-05	antisense	ENSG00000255568.3	103091865
DSCR8	6.32	1.71E-05	protein_coding	ENSG00000198054.11	84677
TFF3	6.27	5.89E-05	protein_coding	ENSG00000160180.15	7033
MRAP	6.25	3.77E-04	protein_coding	ENSG00000170262.12	56246
ADAMTS1	6.25	3.22E-05	protein_coding	ENSG00000154734.14	9510
U2AF1	6.24	8.73E-05	protein_coding	ENSG00000160201.11	7307
DONSON	6.14	1.36E-04	protein_coding	ENSG00000159147.17	29980
C21orf59	6.08	1.95E-05	protein_coding	ENSG00000159079.18	56683
IL10RB	6.04	1.24E-04	protein_coding	ENSG00000243646.9	3588
C21orf2	6.03	8.94E-05	protein_coding	ENSG00000160226.15	755
CH507-9B2.9	5.95	5.89E-05	protein_coding	ENSG00000280433.1	102724200
ADARB1	5.94	2.73E-05	protein_coding	ENSG00000197381.15	104
ANKRD30BP2	5.93	2.59E-05	transcribed_unprocessed_pseudogene	ENSG00000224309.7	NA
AP001347.6	5.90	8.11E-05	antisense	ENSG00000224905.6	NA
LINC01547	5.87	9.20E-05	lincRNA	ENSG00000183250.11	84536
ABCG1	5.77	3.26E-05	protein_coding	ENSG00000160179.18	9619
LRRC3	5.76	1.24E-03	protein_coding	ENSG00000160233.7	81543
PAXBP1-AS1	5.73	5.60E-04	antisense	ENSG00000238197.5	100506215
LCA5L	5.65	4.09E-05	protein_coding	ENSG00000157578.13	150082
AP001065.15	5.46	2.61E-05	lincRNA	ENSG00000228709.1	NA
PPP6R2P1	5.44	1.80E-02	unprocessed_pseudogene	ENSG00000233442.2	NA
RPL23P2	5.43	1.04E-04	processed_pseudogene	ENSG00000176054.6	NA
YBEY	5.37	2.61E-05	protein_coding	ENSG00000182362.13	54059
SAMSN1	5.28	1.00E-03	protein_coding	ENSG00000155307.17	64092
EVA1C	5.25	7.18E-04	protein_coding	ENSG00000166979.12	59271
LINC00205	5.14	6.31E-05	lincRNA	ENSG00000223768.1	NA
FRG2MP	5.13	6.33E-05	processed_pseudogene	ENSG00000275170.1	NA
TSPEAR	5.05	8.00E-04	protein_coding	ENSG00000175894.14	54084
ANKRD20A18P	5.04	7.35E-03	unprocessed_pseudogene	ENSG00000249493.1	NA
RCAN1	4.79	1.37E-03	protein_coding	ENSG00000159200.17	1827
AF015262.2	4.77	1.05E-04	lincRNA	ENSG00000234703.1	NA
BX322557.10	4.74	9.83E-05	processed_transcript	ENSG00000215447.7	NA
TIAM1	4.70	9.70E-04	protein_coding	ENSG00000156299.13	7074
AP001052.1	4.54	1.24E-03	pseudogene	ENSG00000281420.1	NA
AP001627.1	4.50	1.35E-03	antisense	ENSG00000225731.1	NA
TRPM2-AS	4.46	2.67E-03	antisense	ENSG00000230061.2	NA
AP000266.7	4.36	4.84E-03	antisense	ENSG00000232623.1	NA
DSCR4	4.30	2.21E-03	protein_coding	ENSG00000184029.9	10281
LINC01436	4.18	3.58E-04	lincRNA	ENSG00000231106.2	NA
CHAF1B	4.14	9.03E-04	protein_coding	ENSG00000159259.7	8208
AP001412.1	4.12	1.99E-03	antisense	ENSG00000272948.2	NA
SSR4P1	4.03	2.99E-03	transcribed_processed_pseudogene	ENSG00000235374.2	728039
LINC01426	3.92	4.32E-04	antisense	ENSG00000234380.1	100506385
HUNK	3.83	1.63E-02	protein_coding	ENSG00000142149.8	30811
TFF2	3.80	1.22E-02	protein_coding	ENSG00000160181.8	7032
pRNA	3.78	3.54E-03	misc_RNA	ENSG00000277572.1	NA
HSF2BP	3.69	1.98E-03	protein_coding	ENSG00000160207.8	11077
KCNJ15	3.42	5.83E-03	protein_coding	ENSG00000157551.17	3772
RSPH1	3.23	2.14E-02	protein_coding	ENSG00000160188.9	89765
AP000253.1	2.82	8.22E-03	lincRNA	ENSG00000234509.1	NA
AP001437.1	2.74	3.31E-02	antisense	ENSG00000273210.1	NA
AP000254.8	2.56	4.61E-02	antisense	ENSG00000273271.1	NA
AF129075.5	2.44	1.41E-02	sense_intronic	ENSG00000231125.2	NA
H2AFZP1	2.17	3.36E-02	processed_pseudogene	ENSG00000213440.2	NA

Mouse up-regulated genes

Gene Symbol	Log(Fold Change)	Adj.p-Value	Gene Type	GencodeID	EntrezID
Tceal7	8.25	0.007	protein_coding	ENSMUSG00000079428.8	100040972
Mybph	6.91	0.028	protein_coding	ENSMUSG00000042451.12	53311
2310020H05Rik	5.39	0.030	lincRNA	ENSMUSG00000100410.1	NA
Gm13943	3.95	0.048	lincRNA	ENSMUSG00000085277.1	NA
Tmem232	3.77	0.013	protein_coding	ENSMUSG00000045036.14	381107
Emx2os	3.51	0.015	antisense	ENSMUSG00000087095.2	NA
Srpk3	2.83	0.022	protein_coding	ENSMUSG00000002007.5	56504
Aldh1l2	2.73	0.044	protein_coding	ENSMUSG00000020256.14	216188
Klf12	2.54	0.006	protein_coding	ENSMUSG00000072294.4	16597
Hist1h3e	2.45	0.021	protein_coding	ENSMUSG00000069273.2	319151
Hoxa9	1.89	0.032	protein_coding	ENSMUSG00000038227.15	15405
Tpd52l1	1.51	0.027	protein_coding	ENSMUSG00000000296.7	21987
Nudt5	0.82	0.009	protein_coding	ENSMUSG00000025817.12	53893
Cdc123	0.78	0.009	protein_coding	ENSMUSG00000039128.13	98828
Stim1	0.62	0.033	protein_coding	ENSMUSG00000030987.5	20866
Rps27rt	0.53	0.032	protein_coding	ENSMUSG00000050621.6	100043813
Abcb7	0.51	0.026	protein_coding	ENSMUSG00000031333.7	11306
Rnf34	0.50	0.050	protein_coding	ENSMUSG00000029474.7	80751
Sgcb	0.48	0.037	protein_coding	ENSMUSG00000029156.11	24051

Mouse down-regulated genes

Gene Symbol	Log(Fold Change)	Adj.p-Value	Gene Type	GencodeID	EntrezID
Cyp4a12a	-4.00	0.037	protein_coding	ENSMUSG00000066071.6	277753
Cyp2a4	-3.87	0.018	protein_coding	ENSMUSG00000074254.4	13086
C8b	-3.86	0.045	protein_coding	ENSMUSG00000029656.13	110382
Lect2	-3.80	0.047	protein_coding	ENSMUSG00000021539.8	16841
Mmp12	-2.76	0.015	protein_coding	ENSMUSG00000049723.14	17381
Sec1	-2.75	0.032	protein_coding	ENSMUSG00000040364.8	56546
Tmem178	-2.57	0.017	protein_coding	ENSMUSG00000024245.4	68027
Ass1	-2.45	0.027	protein_coding	ENSMUSG00000076441.9	11898
Gm5424	-2.29	0.009	protein_coding	ENSMUSG00000046687.5	NA
Fmn1	-1.60	0.013	protein_coding	ENSMUSG00000044042.18	14260
Epha2	-1.58	0.005	protein_coding	ENSMUSG00000006445.3	13836
Gpnmb	-1.52	0.010	protein_coding	ENSMUSG00000029816.10	93695
Maneal	-1.50	0.032	protein_coding	ENSMUSG00000042763.9	215090
Ccdc85a	-1.27	0.009	protein_coding	ENSMUSG00000032878.16	216613
Pde9a	-1.26	0.009	protein_coding	ENSMUSG00000041119.11	18585
Dnm3	-1.19	0.023	protein_coding	ENSMUSG00000040265.16	103967
Dll1	-1.13	0.031	protein_coding	ENSMUSG00000014773.13	13388
N4bp3	-1.10	0.017	protein_coding	ENSMUSG00000001053.15	212706
Dach1	-1.09	0.024	protein_coding	ENSMUSG00000055639.16	13134
Snai2	-1.07	0.011	protein_coding	ENSMUSG00000022676.6	20583
Prrg3	-1.05	0.025	protein_coding	ENSMUSG00000033361.13	208748
Dock9	-1.04	0.046	protein_coding	ENSMUSG00000025558.16	105445
Gm12922	-1.03	0.026	processed_pseudogene	ENSMUSG00000081170.1	NA
Cd200	-0.98	0.032	protein_coding	ENSMUSG00000022661.14	17470
Tanc1	-0.97	0.022	protein_coding	ENSMUSG00000035168.16	66860
Fmnl1	-0.97	0.031	protein_coding	ENSMUSG00000055805.14	57778
Me2	-0.94	0.004	protein_coding	ENSMUSG00000024556.3	107029
Boc	-0.93	0.041	protein_coding	ENSMUSG00000022687.11	117606
Klf6	-0.91	0.033	protein_coding	ENSMUSG00000000078.6	23849
Pcdh12	-0.87	0.027	protein_coding	ENSMUSG00000024440.12	53601
Jun	-0.84	0.020	protein_coding	ENSMUSG00000052684.4	16476
Lmo2	-0.81	0.043	protein_coding	ENSMUSG00000032698.15	16909
Stxbp1	-0.75	0.034	protein_coding	ENSMUSG00000026797.16	20910
Timp3	-0.74	0.041	protein_coding	ENSMUSG00000020044.13	21859
Myo1b	-0.72	0.039	protein_coding	ENSMUSG00000018417.14	17912
Sytl4	-0.71	0.049	protein_coding	ENSMUSG00000031255.14	27359
Tpst1	-0.69	0.023	protein_coding	ENSMUSG00000034118.15	22021
Sept8	-0.69	0.035	protein_coding	ENSMUSG00000018398.18	20362
1190002N15Rik	-0.67	0.017	protein_coding	ENSMUSG00000045414.7	68861
Slfn5	-0.63	0.024	protein_coding	ENSMUSG00000054404.12	327978
Atp8b2	-0.62	0.046	protein_coding	ENSMUSG00000060671.12	54667
Fam171a1	-0.59	0.046	protein_coding	ENSMUSG00000050530.14	269233
Trim12c	-0.57	0.024	protein_coding	ENSMUSG00000057143.15	319236
Prelp	-0.56	0.036	protein_coding	ENSMUSG00000041577.5	116847
Dusp7	-0.52	0.013	protein_coding	ENSMUSG00000053716.9	235584
Smarcd2	-0.43	0.036	protein_coding	ENSMUSG00000078619.10	83796

bioRxiv preprint doi: https://doi.org/10.1101/2023.01.30.526183; this version posted January 31, 2023. The copyright holder for this preprint (which was not certified by peer review) is the author/funder, who has granted bioRxiv a license to display the preprint in perpetuity. It is made available under aCC-BY 4.0 International license.

Table S10 Skeletal muscle (HFD, 22°C)

Human genes expressed

Gene Symbol	Log(Fold Change)	Adj.p-Value	Gene Type	GencodeID	EntrezID
BRWD1	11.67	1.19E-06	protein_coding	ENSG00000185658.13	54014
SH3BGR	11.39	3.52E-06	protein_coding	ENSG00000185437.13	6450
MAP3K7CL	11.11	3.34E-05	protein_coding	ENSG00000156265.15	56911
ATP5O	11.11	1.19E-06	protein_coding	ENSG00000241837.6	539
AGPAT3	11.05	1.19E-06	protein_coding	ENSG00000160216.18	56894
SOD1	10.92	1.19E-06	protein_coding	ENSG00000142168.14	6647
USP16	10.89	1.19E-06	protein_coding	ENSG00000156256.14	10600
NDUFV3	10.84	1.19E-06	protein_coding	ENSG00000160194.17	4731
PDXK	10.77	1.19E-06	protein_coding	ENSG00000160209.18	8566
ATP5J	10.55	1.19E-06	protein_coding	ENSG00000154723.12	522
UBE2G2	10.51	1.19E-06	protein_coding	ENSG00000184787.18	7327
SON	10.41	1.19E-06	protein_coding	ENSG00000159140.18	6651
PTTG1IP	10.40	3.07E-06	protein_coding	ENSG00000183255.11	754
MCM3AP	10.38	1.19E-06	protein_coding	ENSG00000160294.10	8888
DIP2A	10.34	1.81E-06	protein_coding	ENSG00000160305.17	23181
DSCR3	10.31	1.19E-06	protein_coding	ENSG00000157538.13	10311
SUMO3	10.27	1.81E-06	protein_coding	ENSG00000184900.15	6612
URB1	10.13	1.19E-06	protein_coding	ENSG00000142207.6	9875
HLCS	10.06	1.89E-06	protein_coding	ENSG00000159267.14	3141
RRP1B	10.01	1.45E-06	protein_coding	ENSG00000160208.12	23076
GABPA	9.95	1.19E-06	protein_coding	ENSG00000154727.10	2551
SLC5A3	9.95	3.14E-06	protein_coding	ENSG00000198743.6	6526
GART	9.94	1.19E-06	protein_coding	ENSG00000159131.16	2618
IFNAR1	9.88	1.81E-06	protein_coding	ENSG00000142166.12	3454
TTC3	9.73	3.62E-06	protein_coding	ENSG00000182670.13	7267
PAXBP1	9.72	1.19E-06	protein_coding	ENSG00000159086.14	94104
BACH1	9.66	2.39E-06	protein_coding	ENSG00000156273.15	571
PIGP	9.59	1.19E-06	protein_coding	ENSG00000185808.13	51227
MX2	9.53	1.81E-06	protein_coding	ENSG00000183486.12	4600
LINC00649	9.47	6.36E-06	antisense	ENSG00000237945.7	100506334
ETS2	9.46	8.66E-05	protein_coding	ENSG00000157557.11	2114
POFUT2	9.44	1.19E-06	protein_coding	ENSG00000186866.16	23275
N6AMT1	9.38	1.19E-06	protein_coding	ENSG00000156239.11	29104
TMEM50B	9.35	1.84E-06	protein_coding	ENSG00000142188.16	757
TRAPPC10	9.33	3.29E-06	protein_coding	ENSG00000160218.12	7109
WRB	9.30	1.67E-06	protein_coding	ENSG00000182093.14	7485
ITSN1	9.22	1.81E-06	protein_coding	ENSG00000205726.14	6453
CBR1	9.14	1.41E-06	protein_coding	ENSG00000159228.12	873
SLC37A1	9.14	1.67E-06	protein_coding	ENSG00000160190.13	54020
PCNT	9.12	2.64E-06	protein_coding	ENSG00000160299.16	5116
APP	9.08	5.05E-06	protein_coding	ENSG00000142192.20	351
CCT8	9.06	3.57E-06	protein_coding	ENSG00000156261.12	10694
HMGN1	9.06	2.31E-06	protein_coding	ENSG00000205581.10	3150
C2CD2	8.96	1.88E-06	protein_coding	ENSG00000157617.16	25966
PRMT2	8.80	1.84E-06	protein_coding	ENSG00000160310.16	3275
PLAC4	8.74	2.15E-06	TEC	ENSG00000280109.1	NA
ANKRD30BP2	8.71	3.52E-06	transcribed_unprocessed_pseudogene	ENSG00000224309.7	NA
ADARB1	8.64	1.88E-06	protein_coding	ENSG00000197381.15	104
CLIC6	8.63	5.36E-06	protein_coding	ENSG00000159212.12	54102
CH507-9B2.3	8.62	3.15E-05	protein_coding	ENSG00000280071.3	102724023
SCAF4	8.62	2.75E-06	protein_coding	ENSG00000156304.14	57466
TSPEAR-AS1	8.60	1.84E-06	antisense	ENSG00000235890.2	NA
SYNJ1	8.59	2.67E-06	protein_coding	ENSG00000159082.17	8867
AF121897.4	8.56	7.01E-06	processed_pseudogene	ENSG00000235012.1	NA
MRPL39	8.54	3.29E-06	protein_coding	ENSG00000154719.13	54148
CYYR1	8.54	3.29E-06	protein_coding	ENSG00000166265.11	116159
IFNGR2	8.52	3.11E-06	protein_coding	ENSG00000159128.14	3460
RWDD2B	8.47	3.29E-06	protein_coding	ENSG00000156253.6	10069
SETD4	8.46	3.14E-06	protein_coding	ENSG00000185917.13	54093
PFKL	8.45	4.36E-06	protein_coding	ENSG00000141959.16	5211
PKNOX1	8.44	3.52E-06	protein_coding	ENSG00000160199.14	5316
PRDM15	8.41	4.04E-06	protein_coding	ENSG00000141956.13	63977
MX1	8.37	4.76E-06	protein_coding	ENSG00000157601.13	4599
HSPA13	8.32	3.52E-06	protein_coding	ENSG00000155304.5	6782
RUNX1	8.29	3.29E-06	protein_coding	ENSG00000159216.18	861
CRYZL1	8.28	5.36E-06	protein_coding	ENSG00000205758.11	9946
RRP1	8.28	3.29E-06	protein_coding	ENSG00000160214.12	8568
LTN1	8.17	2.11E-05	protein_coding	ENSG00000198862.13	26046
FAM207A	8.15	3.14E-06	protein_coding	ENSG00000160256.12	85395
ZBTB21	8.14	2.31E-06	protein_coding	ENSG00000173276.13	49854
URB1-AS1	8.12	2.63E-06	lincRNA	ENSG00000256073.3	84996
ADAMTS1	8.07	3.62E-06	protein_coding	ENSG00000154734.14	9510
MRPS6	8.05	5.05E-06	protein_coding	ENSG00000243927.5	64968
PSMG1	8.05	4.70E-06	protein_coding	ENSG00000183527.11	8624
WDR4	7.91	6.19E-06	protein_coding	ENSG00000160193.11	10785
IFNAR2	7.87	1.74E-05	protein_coding	ENSG00000159110.19	3455
CHAF1B	7.84	6.65E-06	protein_coding	ENSG00000159259.7	8208
TSPEAR-AS2	7.82	3.14E-06	antisense	ENSG00000182912.6	NA
JAM2	7.81	3.29E-06	protein_coding	ENSG00000154721.14	58494
DYRK1A	7.79	1.19E-06	protein_coding	ENSG00000157540.19	1859
BACE2	7.72	4.36E-06	protein_coding	ENSG00000182240.15	25825
ITGB2	7.65	4.24E-04	protein_coding	ENSG00000160255.17	3689
DONSON	7.54	4.04E-06	protein_coding	ENSG00000159147.17	29980
DNAJC28	7.44	3.55E-06	protein_coding	ENSG00000177692.11	54943
C21orf33	7.43	4.24E-06	protein_coding	ENSG00000160221.16	8209
S100B	7.27	2.46E-04	protein_coding	ENSG00000160307.9	6285
LCA5L	7.25	4.70E-06	protein_coding	ENSG00000157578.13	150082
DOPEY2	7.22	2.16E-04	protein_coding	ENSG00000142197.12	9980
TIAM1	7.21	1.25E-05	protein_coding	ENSG00000156299.13	7074
UMODL1	7.14	1.61E-05	protein_coding	ENSG00000177398.18	89766
CSTB	7.05	7.03E-05	protein_coding	ENSG00000160213.5	1476
TRPM2	7.04	2.25E-04	protein_coding	ENSG00000142185.16	7226
TSPEAR	7.02	7.01E-06	protein_coding	ENSG00000175894.14	54084
C21orf59	7.02	7.01E-06	protein_coding	ENSG00000159079.18	56683
CH507-9B2.5	6.98	3.24E-04	protein_coding	ENSG00000275464.4	102724159
PAXBP1-AS1	6.82	6.47E-06	antisense	ENSG00000238197.5	100506215
C21orf58	6.79	7.83E-06	protein_coding	ENSG00000160298.17	54058
BX322557.10	6.71	2.91E-05	processed_transcript	ENSG00000215447.7	NA
DSCR4	6.68	4.05E-05	protein_coding	ENSG00000184029.9	10281
BTG3	6.66	1.84E-05	protein_coding	ENSG00000154640.14	10950
EVA1C	6.61	2.11E-05	protein_coding	ENSG00000166979.12	59271
RCAN1	6.56	1.75E-04	protein_coding	ENSG00000159200.17	1827
CYYR1-AS1	6.52	1.67E-05	antisense	ENSG00000197934.8	100996571
POTED	6.47	2.51E-05	protein_coding	ENSG00000166351.10	317754
IL10RB	6.46	3.61E-04	protein_coding	ENSG00000243646.9	3588
LINC00310	6.37	1.26E-04	lincRNA	ENSG00000227456.7	114036
AATBC	6.36	2.07E-05	antisense	ENSG00000215458.8	284837
AP000704.5	6.31	6.58E-05	lincRNA	ENSG00000224790.2	NA
LRRC3	6.30	1.10E-04	protein_coding	ENSG00000160233.7	81543
RSPH1	6.25	6.95E-05	protein_coding	ENSG00000160188.9	89765
AP001065.15	6.20	1.74E-05	lincRNA	ENSG00000228709.1	NA
DSCR8	6.16	1.67E-05	protein_coding	ENSG00000198054.11	84677
MORC3	6.09	1.29E-04	protein_coding	ENSG00000159256.12	23515
KCNE2	6.00	2.26E-05	protein_coding	ENSG00000159197.3	9992
AF015262.2	5.95	4.12E-05	lincRNA	ENSG00000234703.1	NA
ITGB2-AS1	5.93	6.65E-05	antisense	ENSG00000227039.6	100505746
MYL6P2	5.59	5.20E-05	processed_pseudogene	ENSG00000235808.1	NA
SSR4P1	5.50	5.48E-05	transcribed_processed_pseudogene	ENSG00000235374.2	728039
HLCS-IT1	5.47	1.14E-04	sense_intronic	ENSG00000237646.1	NA
HSF2BP	5.46	9.59E-05	protein_coding	ENSG00000160207.8	11077
AP001442.2	5.42	6.76E-05	processed_transcript	ENSG00000233783.7	NA
TFF3	5.34	6.40E-04	protein_coding	ENSG00000160180.15	7033
MIS18A	5.34	1.75E-04	protein_coding	ENSG00000159055.3	54069
AF129075.5	5.27	7.03E-05	sense_intronic	ENSG00000231125.2	NA
AP000469.2	5.26	1.14E-04	lincRNA	ENSG00000224832.1	101927869
AL773572.7	5.21	2.19E-04	antisense	ENSG00000225745.8	NA
AP001625.6	5.19	5.26E-04	antisense	ENSG00000235772.1	NA
LINC01547	5.17	1.32E-04	lincRNA	ENSG00000183250.11	84536
DSCR9	5.15	2.25E-04	lincRNA	ENSG00000230366.9	257203
AP000320.6	5.08	3.61E-04	antisense	ENSG00000225555.1	NA
AP001432.14	5.08	9.59E-05	lincRNA	ENSG00000242553.1	NA
BACH1-IT2	5.06	1.99E-04	lincRNA	ENSG00000228817.4	NA
YBEY	5.04	1.54E-03	protein_coding	ENSG00000182362.13	54059
AP001429.1	5.03	2.68E-04	sense_intronic	ENSG00000270116.1	NA
TRPM2-AS	5.02	3.06E-04	antisense	ENSG00000230061.2	NA
ABCG1	5.01	3.38E-04	protein_coding	ENSG00000160179.18	9619
BRWD1-AS2	4.97	1.52E-04	antisense	ENSG00000255568.3	103091865
RNA5SP491	4.97	1.67E-03	rRNA	ENSG00000199806.1	NA
AP000254.8	4.94	4.15E-04	antisense	ENSG00000273271.1	NA
LINC00205	4.94	1.94E-04	lincRNA	ENSG00000223768.1	NA
AP000692.10	4.84	1.75E-04	antisense	ENSG00000273199.1	NA
BACH1-IT1	4.83	2.66E-04	sense_intronic	ENSG00000248476.1	NA

Mouse up-regulated genes

Gene Symbol	Log(Fold Change)	Adj.p-Value	Gene Type	GencodeID	EntrezID
mt-Tn	7.35	0.001	Mt_tRNA	ENSMUSG00000064348.1	NA
Gm14438	6.08	0.015	processed_pseudogene	ENSMUSG00000084323.4	NA
Th	5.31	0.003	protein_coding	ENSMUSG00000000214.11	21823
mt-Ty	4.71	0.001	Mt_tRNA	ENSMUSG00000064350.1	NA
Fgf21	4.58	0.012	protein_coding	ENSMUSG00000030827.5	56636
mt-Atp8	4.40	0.020	protein_coding	ENSMUSG00000064356.3	17706
H2-Q2	3.96	0.048	protein_coding	ENSMUSG00000091705.8	15013
Sln	3.94	0.004	protein_coding	ENSMUSG00000042045.5	66402
Dbh	3.76	0.040	protein_coding	ENSMUSG00000000889.8	13166
mt-Ts1	3.76	0.007	Mt_tRNA	ENSMUSG00000064352.1	NA
Dmp1	3.74	0.001	protein_coding	ENSMUSG00000029307.7	13406
4930477O15Rik	3.72	0.012	TEC, lincRNA	ENSMUSG00000104999.1	NA
Flywch2	3.67	0.005	protein_coding	ENSMUSG00000023911.12	76917
Ccdc92	3.64	0.000	protein_coding	ENSMUSG00000037979.13	215707
mt-Co2	3.58	0.020	protein_coding	ENSMUSG00000064354.1	17709
Cyp4f14	3.57	0.014	protein_coding	ENSMUSG00000024292.14	64385
Tmem181c-ps	3.52	0.006	unprocessed_pseudogene	ENSMUSG00000093880.2	NA
Rpl17-ps8	3.52	0.033	processed_pseudogene	ENSMUSG00000082035.1	NA
Gm43694	3.49	0.042	TEC	ENSMUSG00000107018.1	NA
Fam174b	3.30	0.000	protein_coding	ENSMUSG00000078670.3	100038347
mt-Atp6	3.18	0.019	protein_coding	ENSMUSG00000064357.1	17705
Gm11127	3.16	0.007	protein_coding	ENSMUSG00000079492.2	100529082
Mthfd2	2.90	0.001	protein_coding	ENSMUSG00000005667.8	17768
mt-Nd4l	2.90	0.050	protein_coding	ENSMUSG00000065947.3	17720
Gm42853	2.80	0.025	TEC	ENSMUSG00000107021.1	NA
Gm22989	2.77	0.021	snRNA	ENSMUSG00000088938.1	NA
Srrm4os	2.76	0.002	antisense	ENSMUSG00000086219.7	76798
Asns	2.74	0.011	protein_coding	ENSMUSG00000029752.12	27053
Myo1a	2.70	0.006	protein_coding	ENSMUSG00000025401.8	432516
Crip3	2.67	0.000	protein_coding	ENSMUSG00000023968.15	114570
Apol9a	2.66	0.011	protein_coding	ENSMUSG00000057346.11	223672
Gm21738	2.61	0.018	protein_coding	ENSMUSG00000095280.1	NA
1810041L15Rik	2.59	0.001	protein_coding	ENSMUSG00000062760.10	72301
Krt18	2.57	0.007	protein_coding	ENSMUSG00000023043.6	16668
Gm43013	2.52	0.031	processed_pseudogene	ENSMUSG00000106058.1	NA
Cilp	2.44	0.010	protein_coding	ENSMUSG00000042254.14	214425
Gm10801	2.43	0.005	protein_coding	ENSMUSG00000075015.3	NA
Apol9b	2.41	0.045	protein_coding	ENSMUSG00000068246.5	71898
Slco4a1	2.39	0.004	protein_coding	ENSMUSG00000038963.15	108115
Mogat1	2.36	0.003	protein_coding	ENSMUSG00000012187.13	68393
E2f2	2.36	0.000	protein_coding	ENSMUSG00000018983.9	242705
Gm5420	2.35	0.003	lincRNA	ENSMUSG00000056716.9	432436
Srrm4	2.31	0.005	protein_coding	ENSMUSG00000063919.10	68955
Gm10720	2.27	0.048	protein_coding	ENSMUSG00000074564.3	NA
Cpne2	2.27	0.000	protein_coding	ENSMUSG00000034361.10	234577
H2-T-ps	2.27	0.024	unprocessed_pseudogene	ENSMUSG00000073405.6	667803
Gm11734	2.26	0.000	lincRNA	ENSMUSG00000085419.1	NA
2310043O21Rik	2.26	0.012	lincRNA	ENSMUSG00000102106.1	69679
1110002O04Rik	2.23	0.033	lincRNA	ENSMUSG00000102531.1	NA
BC035947	2.22	0.007	protein_coding	ENSMUSG00000090486.2	NA
Zdbf2	2.17	0.000	protein_coding	ENSMUSG00000027520.15	73884
Gm10800	2.16	0.000	protein_coding	ENSMUSG00000075014.1	NA
Ifi44	2.16	0.005	protein_coding	ENSMUSG00000028037.13	99899
Mir344c	2.15	0.042	miRNA	ENSMUSG00000077107.1	100526506
A930003A15Rik	2.10	0.002	protein_coding	ENSMUSG00000075330.3	NA
Kcnk1	2.10	0.004	protein_coding	ENSMUSG00000033998.9	16525
Gm10425	2.09	0.001	lincRNA	ENSMUSG00000097081.1	NA
Adamts20	2.07	0.000	protein_coding	ENSMUSG00000022449.14	223838
Tceal7	2.05	0.000	protein_coding	ENSMUSG00000079428.8	100040972
A330076H08Rik	2.02	0.004	processed_transcript	ENSMUSG00000109321.1	320026
Gm29228	1.99	0.041	processed_pseudogene	ENSMUSG00000101682.1	NA
Isg15	1.99	0.030	protein_coding	ENSMUSG00000035692.6	100038882
Irf7	1.94	0.004	protein_coding	ENSMUSG00000025498.15	54123
H2-Q10	1.87	0.030	protein_coding	ENSMUSG00000067235.14	15007
Ankrd1	1.86	0.000	protein_coding	ENSMUSG00000024803.8	107765
AW551984	1.81	0.050	protein_coding	ENSMUSG00000038112.15	244810
3632451O06Rik	1.77	0.004	protein_coding	ENSMUSG00000036242.14	67419
Cdkn1a	1.77	0.005	protein_coding	ENSMUSG00000023067.13	12575
RP23-54G8.4	1.76	0.006	lincRNA	ENSMUSG00000109394.1	319893
1500012K07Rik	1.74	0.047	processed_transcript	ENSMUSG00000097247.2	68954
F830016B08Rik	1.73	0.001	protein_coding	ENSMUSG00000090942.1	240328
Adig	1.71	0.012	protein_coding	ENSMUSG00000044405.4	246747
mt-Th	1.70	0.010	Mt_tRNA	ENSMUSG00000064364.1	NA
Fam69c	1.69	0.036	protein_coding	ENSMUSG00000047992.10	240479
Slc7a5	1.69	0.003	protein_coding	ENSMUSG00000040010.10	20539
Gm2399	1.64	0.024	protein_coding	ENSMUSG00000078141.2	NA
Exoc3l4	1.64	0.004	protein_coding	ENSMUSG00000021280.8	74190
Apln	1.61	0.016	protein_coding	ENSMUSG00000037010.7	30878
Palm2	1.60	0.025	protein_coding	ENSMUSG00000090053.9	677884
Cxcl10	1.57	0.028	protein_coding	ENSMUSG00000034855.13	15945
Acbd7	1.57	0.041	protein_coding	ENSMUSG00000026644.6	78245
Gm15420	1.56	0.047	antisense	ENSMUSG00000087387.1	NA
Gm9825	1.56	0.009	processed_pseudogene	ENSMUSG00000096403.2	NA
Oas2	1.55	0.003	protein_coding	ENSMUSG00000032690.16	246728
Slc1a1	1.55	0.016	protein_coding	ENSMUSG00000024935.10	20510
Oasl1	1.55	0.021	protein_coding	ENSMUSG00000041827.15	231655
Thrsp	1.53	0.027	protein_coding	ENSMUSG00000035686.8	21835
Trib3	1.53	0.006	protein_coding	ENSMUSG00000032715.9	228775
Arf4os	1.52	0.034	antisense	ENSMUSG00000090861.1	NA
Tnfaip2	1.49	0.000	protein_coding	ENSMUSG00000021281.15	21928
Gm4742	1.48	0.032	processed_pseudogene	ENSMUSG00000105461.1	NA
Ift122	1.46	0.000	protein_coding	ENSMUSG00000030323.13	81896
Bdnf	1.46	0.031	protein_coding	ENSMUSG00000048482.14	12064
B230311B06Rik	1.46	0.038	lincRNA	ENSMUSG00000109284.1	381914
Gadd45a	1.44	0.000	protein_coding	ENSMUSG00000036390.8	13197
Gabrr2	1.42	0.013	protein_coding	ENSMUSG00000023267.10	14409
Rtp4	1.40	0.001	protein_coding	ENSMUSG00000033355.6	67775
RP23-354I24.7	1.40	0.005	lincRNA	ENSMUSG00000110656.1	NA
Enkur	1.40	0.046	protein_coding	ENSMUSG00000026679.3	71233
RP24-116C19.5	1.39	0.040	protein_coding	ENSMUSG00000109739.1	NA
Trpv3	1.39	0.005	protein_coding	ENSMUSG00000043029.2	246788
4931431B13Rik	1.38	0.014	processed_transcript	ENSMUSG00000108354.1	70973
Aldh18a1	1.37	0.017	protein_coding	ENSMUSG00000025007.13	56454
Ifit3	1.37	0.013	protein_coding	ENSMUSG00000074896.3	15959
Zfp57	1.36	0.019	protein_coding	ENSMUSG00000036036.15	22715
H2-T10	1.36	0.035	polymorphic_pseudogene	ENSMUSG00000079491.9	NA
Gm42509	1.34	0.017	TEC	ENSMUSG00000105042.1	NA
Clu	1.31	0.000	protein_coding	ENSMUSG00000022037.14	12759
Stk36	1.30	0.018	protein_coding	ENSMUSG00000033276.18	269209
Gm4925	1.26	0.048	transcribed_processed_pseudogene	ENSMUSG00000069540.4	237433
2010300C02Rik	1.25	0.011	protein_coding	ENSMUSG00000026090.16	72097
Gm26704	1.23	0.037	lincRNA	ENSMUSG00000097875.1	NA
Spag5	1.21	0.002	protein_coding	ENSMUSG00000002055.9	54141
Tiam2	1.21	0.004	protein_coding	ENSMUSG00000023800.14	24001
Gm7993	1.19	0.033	processed_pseudogene	ENSMUSG00000107092.1	NA
Hba-ps4	1.19	0.035	unprocessed_pseudogene	ENSMUSG00000084893.3	NA
RP23-385F3.1	1.19	0.003	processed_pseudogene	ENSMUSG00000110131.1	NA
Tbc1d31	1.19	0.002	protein_coding	ENSMUSG00000022364.14	210544
Chrna10	1.18	0.002	protein_coding	ENSMUSG00000066279.3	504186
RP23-385G14.1	1.18	0.007	transcribed_unprocessed_pseudogene	ENSMUSG00000110298.1	NA
Slc47a1	1.17	0.002	protein_coding	ENSMUSG00000010122.14	67473
Oasl2	1.17	0.011	protein_coding	ENSMUSG00000029561.17	23962
Sox4	1.17	0.038	protein_coding	ENSMUSG00000076431.4	20677
Spock3	1.17	0.033	protein_coding	ENSMUSG00000054162.15	72902
Camta1	1.16	0.005	protein_coding	ENSMUSG00000014592.19	100072
Tmem8	1.15	0.012	protein_coding	ENSMUSG00000024180.13	60455
Ak4	1.14	0.047	protein_coding	ENSMUSG00000028527.18	11639
Itga9	1.13	0.002	protein_coding	ENSMUSG00000039115.13	104099
Tnfrsf19	1.13	0.001	protein_coding	ENSMUSG00000060548.12	29820
Gnmt	1.11	0.035	protein_coding	ENSMUSG00000002769.8	14711
5330439K02Rik	1.11	0.046	lincRNA	ENSMUSG00000097827.1	NA
4930412C18Rik	1.11	0.016	processed_transcript	ENSMUSG00000085558.7	NA
6430548M08Rik	1.11	0.003	protein_coding	ENSMUSG00000031824.15	234797
Armcx4	1.11	0.001	protein_coding	ENSMUSG00000049804.9	100503043
Apom	1.10	0.040	protein_coding	ENSMUSG00000024391.7	55938
Tppp3	1.09	0.001	protein_coding	ENSMUSG00000014846.12	67971
Gm45010	1.09	0.037	TEC	ENSMUSG00000109015.1	NA
Pbld2	1.08	0.030	protein_coding	ENSMUSG00000020072.14	67307
Otud1	1.08	0.002	protein_coding	ENSMUSG00000043415.5	71198
Clec18a	1.07	0.006	protein_coding	ENSMUSG00000033633.13	353287
Tmem82	1.07	0.031	protein_coding	ENSMUSG00000043085.14	213989
Gm18301	1.07	0.044	unprocessed_pseudogene	ENSMUSG00000099569.1	NA

Mouse down-regulated genes

Gene Symbol	Log(Fold Change)	Adj.p-Value	Gene Type	GencodeID	EntrezID
Themis3	-4.90	0.033	protein_coding	ENSMUSG00000024105.2	74556
Calm4	-4.83	0.020	protein_coding	ENSMUSG00000033765.4	80796
Gm38081	-4.07	0.008	TEC	ENSMUSG00000104363.1	NA
Ly6g6d	-3.73	0.016	protein_coding	ENSMUSG00000073413.9	114654
Gm4861	-3.67	0.011	protein_coding	ENSMUSG00000055138.9	229862
Dkk3	-3.58	0.000	protein_coding	ENSMUSG00000030772.6	50781
1700001O22Rik	-3.53	0.004	protein_coding	ENSMUSG00000044320.14	73598
Slc35f3	-3.49	0.027	protein_coding	ENSMUSG00000057060.7	210027
Fgfbp1	-3.45	0.014	protein_coding	ENSMUSG00000048373.8	14181
Mss51	-3.41	0.001	protein_coding	ENSMUSG00000021815.3	74843
Cdh4	-3.31	0.000	protein_coding	ENSMUSG00000000305.12	12561
Pld5	-3.24	0.001	protein_coding	ENSMUSG00000055214.15	319455
Gm44400	-3.22	0.021	TEC	ENSMUSG00000107488.1	NA
Gm43521	-3.21	0.029	TEC	ENSMUSG00000106511.1	NA
RP23-110M15.1	-3.19	0.030	lincRNA	ENSMUSG00000109853.1	102635797
Gm1604a	-3.09	0.025	protein_coding	ENSMUSG00000094083.1	381059
Sorcs1	-3.07	0.006	protein_coding	ENSMUSG00000043531.16	58178
Cntnap2	-2.84	0.002	protein_coding	ENSMUSG00000039419.17	66797
Gm20109	-2.80	0.032	lincRNA	ENSMUSG00000097714.1	NA
Neto2	-2.75	0.004	protein_coding	ENSMUSG00000036902.10	74513
Lrtm2	-2.71	0.046	protein_coding	ENSMUSG00000055003.14	211187
Odf3l2	-2.71	0.001	protein_coding	ENSMUSG00000035963.8	382384
Col9a1	-2.66	0.025	protein_coding	ENSMUSG00000026147.16	12839
Fggy	-2.66	0.001	protein_coding	ENSMUSG00000028573.18	75578
Syt9	-2.65	0.017	protein_coding	ENSMUSG00000062542.11	60510
Pla2g7	-2.64	0.000	protein_coding	ENSMUSG00000023913.17	27226
Slc2a3	-2.59	0.001	protein_coding	ENSMUSG00000003153.10	20527
Ighm	-2.58	0.001	IG_C_gene	ENSMUSG00000076617.9	NA
Gm10478	-2.52	0.042	pseudogene	ENSMUSG00000073179.3	NA
Gm38048	-2.49	0.005	sense_overlapping	ENSMUSG00000102437.1	NA
Shc2	-2.44	0.000	protein_coding	ENSMUSG00000020312.11	216148
Mdga1	-2.41	0.000	protein_coding	ENSMUSG00000043557.15	74762
Prima1	-2.41	0.013	protein_coding	ENSMUSG00000041669.15	170952
Cacna2d4	-2.38	0.029	protein_coding	ENSMUSG00000041460.14	319734
Gck	-2.34	0.002	protein_coding	ENSMUSG00000041798.15	103988
Slc4a10	-2.34	0.000	protein_coding	ENSMUSG00000026904.17	94229
Amd1	-2.32	0.000	protein_coding	ENSMUSG00000075232.4	11702
Snph	-2.31	0.003	protein_coding	ENSMUSG00000027457.15	241727
Amd2	-2.25	0.001	protein_coding	ENSMUSG00000063953.2	100041585
Mir694	-2.17	0.034	miRNA	ENSMUSG00000076147.1	751558
Mettl11b	-2.17	0.000	protein_coding	ENSMUSG00000040113.14	240879
9830004L10Rik	-2.16	0.001	lincRNA	ENSMUSG00000099552.1	NA
Gm26995	-2.15	0.050	antisense	ENSMUSG00000098072.1	NA
Tmem100	-2.15	0.002	protein_coding	ENSMUSG00000069763.3	67888
Pla2g5	-2.13	0.009	protein_coding	ENSMUSG00000041193.15	18784
2310065F04Rik	-2.12	0.000	lincRNA	ENSMUSG00000087410.7	74184
Amd-ps1	-2.07	0.005	processed_pseudogene	ENSMUSG00000083823.3	NA
Hist1h4n	-2.05	0.045	protein_coding	ENSMUSG00000069305.3	319161
Exph5	-2.04	0.001	protein_coding	ENSMUSG00000034584.3	320051
Amd-ps4	-2.03	0.020	processed_pseudogene	ENSMUSG00000019836.5	NA
A330015K06Rik	-2.03	0.004	antisense	ENSMUSG00000104093.1	102637797
Stk26	-2.03	0.000	protein_coding	ENSMUSG00000031112.10	70415
Wnt16	-2.01	0.005	protein_coding	ENSMUSG00000029671.15	93735
Nhs	-2.00	0.000	protein_coding	ENSMUSG00000059493.13	195727
Mib1	-1.99	0.000	protein_coding	ENSMUSG00000024294.13	225164
Igfn1	-1.99	0.006	protein_coding	ENSMUSG00000051985.12	226438
Gm38106	-1.98	0.025	TEC	ENSMUSG00000102266.1	NA
Tmem132b	-1.90	0.013	protein_coding	ENSMUSG00000070498.3	208151
Tpx2	-1.86	0.000	protein_coding	ENSMUSG00000027469.16	72119
M1ap	-1.85	0.006	protein_coding	ENSMUSG00000030041.9	110958
1700071M16Rik	-1.84	0.001	antisense	ENSMUSG00000090307.7	73504
Gm15034	-1.83	0.012	processed_pseudogene	ENSMUSG00000081320.1	NA
Gm42855	-1.83	0.047	processed_pseudogene	ENSMUSG00000105706.1	NA
Gdf11	-1.83	0.000	protein_coding	ENSMUSG00000025352.6	14561
Smox	-1.82	0.002	protein_coding	ENSMUSG00000027333.18	228608
Kcnab1	-1.82	0.001	protein_coding	ENSMUSG00000027827.16	16497
RP23-261B5.1	-1.81	0.000	TEC	ENSMUSG00000110411.1	NA
Mstn	-1.81	0.000	protein_coding	ENSMUSG00000026100.6	17700
Sorbs2	-1.77	0.000	protein_coding	ENSMUSG00000031626.17	234214
Amd-ps3	-1.76	0.005	processed_pseudogene	ENSMUSG00000060096.4	NA
Gm38158	-1.74	0.004	processed_pseudogene	ENSMUSG00000103367.1	NA
Uts2r	-1.70	0.037	protein_coding	ENSMUSG00000039321.1	217369
Gm5432	-1.69	0.017	lincRNA	ENSMUSG00000097580.1	NA
Rasd2	-1.69	0.001	protein_coding	ENSMUSG00000034472.13	75141
Fam83d	-1.67	0.007	protein_coding	ENSMUSG00000027654.2	71878
Ctxn3	-1.67	0.000	protein_coding	ENSMUSG00000069372.3	629147
Casr	-1.66	0.035	protein_coding	ENSMUSG00000051980.13	12374
Gm43699	-1.64	0.028	lincRNA	ENSMUSG00000107244.1	NA
Syt3	-1.62	0.002	protein_coding	ENSMUSG00000030731.13	20981
Ighg2c	-1.62	0.050	IG_C_gene	ENSMUSG00000076612.7	NA
Aurka	-1.62	0.000	protein_coding	ENSMUSG00000027496.15	20878
Adamts3	-1.61	0.012	protein_coding	ENSMUSG00000043635.12	330119
Rmi2	-1.61	0.030	protein_coding	ENSMUSG00000037991.9	223970
Pmepa1	-1.60	0.003	protein_coding	ENSMUSG00000038400.15	65112
Ccnk	-1.58	0.000	protein_coding	ENSMUSG00000021258.9	12454
Gm37053	-1.56	0.010	TEC	ENSMUSG00000103348.1	NA
Tiam1	-1.55	0.000	protein_coding	ENSMUSG00000002489.15	21844
Nr4a2	-1.55	0.019	protein_coding	ENSMUSG00000026826.13	18227
Hist1h4m	-1.55	0.003	protein_coding	ENSMUSG00000069306.5	100041230
Ttll7	-1.53	0.001	protein_coding	ENSMUSG00000036745.15	70892
Gm11512	-1.51	0.026	processed_pseudogene	ENSMUSG00000081043.1	NA
2310016D03Rik	-1.50	0.037	lincRNA	ENSMUSG00000102098.1	69566
Tnmd	-1.50	0.011	protein_coding	ENSMUSG00000031250.8	64103
Nos1	-1.49	0.001	protein_coding	ENSMUSG00000029361.18	18125
Sorbs2os	-1.48	0.001	antisense	ENSMUSG00000085440.1	319940
Macrod2os2	-1.47	0.021	antisense	ENSMUSG00000074763.5	NA
Fam65b	-1.46	0.008	protein_coding	ENSMUSG00000036006.19	193385
Mc2r	-1.45	0.004	protein_coding	ENSMUSG00000045569.6	17200
Syp	-1.44	0.008	protein_coding	ENSMUSG00000031144.15	20977
Kcnf1	-1.43	0.049	protein_coding	ENSMUSG00000051726.6	382571
Gdap1	-1.42	0.006	protein_coding	ENSMUSG00000025777.8	14545
Grem2	-1.42	0.009	protein_coding	ENSMUSG00000050069.3	23893
Kcnc3	-1.42	0.006	protein_coding	ENSMUSG00000062785.14	16504
Gm15824	-1.42	0.017	processed_pseudogene	ENSMUSG00000083435.2	NA
Gm7329	-1.42	0.033	processed_pseudogene	ENSMUSG00000100774.1	NA
Hr	-1.41	0.000	protein_coding	ENSMUSG00000022096.14	15460
Hsd17b7	-1.41	0.001	protein_coding	ENSMUSG00000026675.12	15490
Hlf	-1.40	0.001	protein_coding	ENSMUSG00000003949.16	217082
4933415F23Rik	-1.38	0.033	protein_coding	ENSMUSG00000073730.2	66755
Ptx4	-1.37	0.038	protein_coding	ENSMUSG00000044172.6	68509
Ankrd34a	-1.36	0.030	protein_coding	ENSMUSG00000049097.9	545554
Gm12199	-1.36	0.034	antisense	ENSMUSG00000087080.1	NA
Col22a1	-1.33	0.002	protein_coding	ENSMUSG00000079022.9	69700
Thbs4	-1.33	0.001	protein_coding	ENSMUSG00000021702.7	21828
Col24a1	-1.33	0.013	protein_coding	ENSMUSG00000028197.4	71355
Me1	-1.33	0.000	protein_coding	ENSMUSG00000032418.15	17436
Adgrf4	-1.32	0.007	protein_coding	ENSMUSG00000023918.12	78249
Gadl1	-1.32	0.012	protein_coding	ENSMUSG00000056880.12	73748
Pkp2	-1.31	0.002	protein_coding	ENSMUSG00000041957.15	67451
Fat3	-1.31	0.001	protein_coding	ENSMUSG00000074505.4	270120
Cacng7	-1.30	0.002	protein_coding	ENSMUSG00000069806.5	81904
Enox2	-1.30	0.000	protein_coding	ENSMUSG00000031109.16	209224
Aox3	-1.30	0.019	protein_coding	ENSMUSG00000064294.12	71724
Rtn4r	-1.30	0.002	protein_coding	ENSMUSG00000043811.5	65079
Ddah1	-1.29	0.043	protein_coding	ENSMUSG00000028194.15	69219
Gcnt1	-1.28	0.025	protein_coding	ENSMUSG00000038843.17	14537
Morn4	-1.27	0.002	protein_coding	ENSMUSG00000049670.8	226123
RP23-220F20.2	-1.26	0.016	protein_coding	ENSMUSG00000110344.1	NA
Slc16a3	-1.26	0.001	protein_coding	ENSMUSG00000025161.16	80879
Kera	-1.25	0.003	protein_coding	ENSMUSG00000019932.7	16545
Gm14698	-1.25	0.000	protein_coding	ENSMUSG00000071748.2	NA
Gpnmb	-1.23	0.029	protein_coding	ENSMUSG00000029816.10	93695
Gm7135	-1.23	0.045	transcribed_unprocessed_pseudogene	ENSMUSG00000101348.6	634346
Gpcpd1	-1.22	0.000	protein_coding	ENSMUSG00000027346.15	74182
Tubb2b	-1.21	0.048	protein_coding	ENSMUSG00000045136.5	73710
Myl4	-1.21	0.033	protein_coding	ENSMUSG00000061086.12	17896
C530050E15Rik	-1.20	0.010	lincRNA	ENSMUSG00000097838.1	NA
Adra1a	-1.20	0.010	protein_coding	ENSMUSG00000045875.12	11549
Nov	-1.19	0.026	protein_coding	ENSMUSG00000037362.8	18133
Eif3j1	-1.19	0.021	protein_coding	ENSMUSG00000027236.8	78655
Junos	-1.19	0.000	antisense	ENSMUSG00000087366.7	NA
Tbc1d1	-1.18	0.005	protein_coding	ENSMUSG00000029174.18	57915

bioRxiv preprint doi: https://doi.org/10.1101/2023.01.30.526183; this version posted January 31, 2023. The copyright holder for this preprint (which was not certified by peer review) is the author/funder, who has granted bioRxiv a license to display the preprint in perpetuity. It is made available under aCC-BY 4.0 International license.

METTL21AP1	4.82	3.76E-03	processed_pseudogene	ENSG00000229623.1	NA
CBR3	4.81	1.77E-04	protein_coding	ENSG00000159231.5	874
ZNF295-AS1	4.73	4.51E-04	lincRNA	ENSG00000237232.7	150142
AL109761.5	4.69	2.40E-04	antisense	ENSG00000244676.5	NA
GRIK1-AS1	4.68	3.51E-04	antisense	ENSG00000174680.9	NA
H2AFZP1	4.55	3.21E-04	processed_pseudogene	ENSG00000213440.2	NA
BACH1-AS1	4.53	4.86E-04	lincRNA	ENSG00000232118.2	NA
DSTNP1	4.52	3.63E-04	processed_pseudogene	ENSG00000230982.1	NA
RPS26P4	4.49	4.77E-04	processed_pseudogene	ENSG00000228349.1	NA
PCBP2P1	4.49	5.03E-04	processed_pseudogene	ENSG00000235701.1	NA
TTC3-AS1	4.49	7.20E-04	antisense	ENSG00000228677.1	NA
RIMKLBP1	4.46	1.05E-03	processed_pseudogene	ENSG00000189089.5	NA
BRWD1-IT1	4.42	4.90E-04	sense_overlapping	ENSG00000237373.1	NA
DIP2A-IT1	4.40	8.06E-04	sense_intronic	ENSG00000223692.1	100862692
RP5-1023B21.1	4.33	1.74E-03	lincRNA	ENSG00000267857.2	NA
AF015720.3	4.23	7.95E-04	lincRNA	ENSG00000230794.1	NA
AP000240.9	4.21	5.58E-04	sense_intronic	ENSG00000273017.1	NA
LINC01436	4.21	4.29E-03	lincRNA	ENSG00000231106.2	NA
AF064858.11	4.12	6.34E-04	lincRNA	ENSG00000237721.1	NA
RNU6-859P	4.10	7.22E-04	snRNA	ENSG00000199598.1	NA
AP001505.10	4.09	2.75E-03	lincRNA	ENSG00000276529.1	NA
AP000302.58	4.07	1.51E-03	antisense	ENSG00000231355.1	NA
KB-68A7.2	3.99	2.67E-03	lincRNA	ENSG00000277352.1	NA
TIMM9P2	3.96	3.39E-03	processed_pseudogene	ENSG00000232608.1	NA
LINC00189	3.91	4.13E-03	sense_overlapping	ENSG00000215533.8	193629
LINC00334	3.82	1.42E-03	lincRNA	ENSG00000182586.7	114042
HMGN1P2	3.78	2.16E-03	processed_pseudogene	ENSG00000229046.1	NA
RPS20P1	3.75	2.09E-03	processed_pseudogene	ENSG00000229761.1	NA
RPS5P2	3.70	2.45E-03	processed_pseudogene	ENSG00000224598.1	NA
RPL23P2	3.70	1.91E-03	processed_pseudogene	ENSG00000176054.6	NA
SNORA81	3.49	4.34E-03	snoRNA	ENSG00000238390.1	NA
RNU6-614P	3.43	4.75E-03	snRNA	ENSG00000207097.1	NA
BTF3P6	3.43	2.55E-03	processed_pseudogene	ENSG00000233956.1	NA
BACH1-IT3	3.40	3.97E-03	sense_intronic	ENSG00000234293.1	NA
AP001412.1	3.36	3.40E-03	antisense	ENSG00000272948.2	NA
AP001437.1	3.35	9.28E-03	antisense	ENSG00000273210.1	NA
AF124730.4	3.27	9.00E-03	antisense	ENSG00000224649.1	NA
RP1-100J12.1	3.18	5.00E-03	antisense	ENSG00000273254.1	NA
AP001619.2	3.18	4.13E-03	sense_intronic	ENSG00000227698.1	NA
AP001619.3	3.05	8.81E-03	antisense	ENSG00000236545.1	NA
FGF7P2	2.88	2.98E-02	unprocessed_pseudogene	ENSG00000185390.2	NA
DPRXP5	2.79	4.53E-02	processed_pseudogene	ENSG00000270652.1	NA
U3	2.74	1.81E-02	snoRNA	ENSG00000212479.2	NA
AP001625.4	2.59	3.25E-02	sense_intronic	ENSG00000239930.2	101930094
RNU6-123P	2.48	3.98E-02	snRNA	ENSG00000251972.1	NA
SNORA80A	2.40	4.11E-02	snoRNA	ENSG00000200792.1	677846
AP001439.2	2.33	4.77E-02	antisense	ENSG00000224541.1	NA

Serpinb1a	1.06	0.026	protein_coding	ENSMUSG00000044734.15	66222
Gm37691	1.06	0.005	TEC	ENSMUSG00000104348.1	NA
Rapgefl1	1.05	0.033	protein_coding	ENSMUSG00000038020.5	268480
Fam83g	1.04	0.004	protein_coding	ENSMUSG00000042377.8	69640
Sh2d4a	1.04	0.006	protein_coding	ENSMUSG00000053886.4	72281
A830010M20Rik	1.04	0.004	protein_coding	ENSMUSG00000044060.18	NA
Sbsn	1.03	0.031	protein_coding	ENSMUSG00000046056.15	282619
Rapgef4os3	1.03	0.038	antisense	ENSMUSG00000086231.1	NA
Katnal2	1.03	0.015	protein_coding	ENSMUSG00000025420.13	71206
Gm15543	1.02	0.029	antisense	ENSMUSG00000086863.1	105242817
Snai3	1.01	0.001	protein_coding	ENSMUSG00000006587.6	30927
Adam1b	1.01	0.046	protein_coding	ENSMUSG00000062438.4	280667
Atf5	1.01	0.008	protein_coding	ENSMUSG00000038539.15	107503
Mx2	1.00	0.038	polymorphic_pseudogene	ENSMUSG00000023341.14	17858
Tmem116	1.00	0.014	protein_coding	ENSMUSG00000029452.18	77462
Fam166a	1.00	0.047	protein_coding	ENSMUSG00000026969.3	68222
Gm14762	1.00	0.042	lincRNA	ENSMUSG00000078314.2	NA
Zfp69	1.00	0.043	protein_coding	ENSMUSG00000064141.14	381549
0610040B10Rik	1.00	0.004	antisense	ENSMUSG00000089889.2	NA
Gm15860	1.00	0.026	antisense	ENSMUSG00000087336.2	NA
Mterf1a	0.99	0.033	protein_coding	ENSMUSG00000040429.6	545725
Mogat2	0.99	0.032	protein_coding	ENSMUSG00000052396.7	233549
Iigp1	0.99	0.032	protein_coding	ENSMUSG00000054072.11	60440
Arid5a	0.98	0.001	protein_coding	ENSMUSG00000037447.16	214855
Gm38250	0.98	0.049	antisense	ENSMUSG00000104168.1	NA
Vash2	0.97	0.036	protein_coding	ENSMUSG00000037568.12	226841
Lrp2bp	0.97	0.001	protein_coding	ENSMUSG00000031637.13	67620
5031434C07Rik	0.97	0.006	antisense	ENSMUSG00000044574.5	NA
Gm5617	0.97	0.005	protein_coding	ENSMUSG00000042293.7	434402
Rad51	0.97	0.033	protein_coding	ENSMUSG00000027323.10	19361
Osbp2	0.97	0.017	protein_coding	ENSMUSG00000020435.17	74309
Pdp2	0.97	0.006	protein_coding	ENSMUSG00000048371.8	382051
Lsmem1	0.96	0.000	protein_coding	ENSMUSG00000071342.4	380755
Dqx1	0.95	0.030	protein_coding	ENSMUSG00000009145.6	93838
Actn2	0.95	0.006	protein_coding	ENSMUSG00000052374.14	11472
Cmpk2	0.95	0.025	protein_coding	ENSMUSG00000020638.7	22169
Tlcd2	0.94	0.022	protein_coding	ENSMUSG00000038217.13	380712
Prkcz	0.94	0.042	protein_coding	ENSMUSG00000029053.16	18762
Aqp4	0.94	0.021	protein_coding	ENSMUSG00000024411.9	11829
E230016M11Rik	0.94	0.016	processed_transcript	ENSMUSG00000087231.7	320172
1810021B22Rik	0.94	0.011	processed_transcript	ENSMUSG00000087331.8	69120
Dnaja4	0.93	0.005	protein_coding	ENSMUSG00000032285.15	58233
Xirp1	0.93	0.007	protein_coding	ENSMUSG00000079243.2	22437
Gm37759	0.93	0.036	antisense	ENSMUSG00000102717.1	NA
Plin3	0.93	0.002	protein_coding	ENSMUSG00000024197.9	66905
Gm45061	0.93	0.030	TEC	ENSMUSG00000108075.1	NA
Gm42974	0.92	0.033	TEC	ENSMUSG00000104963.1	NA
Bbc3	0.92	0.024	protein_coding	ENSMUSG00000002083.13	170770
4930451G09Rik	0.91	0.019	protein_coding	ENSMUSG00000022543.8	74684
Gm44193	0.91	0.016	TEC	ENSMUSG00000107559.1	NA
Ccdc189	0.90	0.043	protein_coding	ENSMUSG00000057176.4	233899
Tmem71	0.90	0.003	protein_coding	ENSMUSG00000036944.5	213068
Actc1	0.90	0.025	protein_coding	ENSMUSG00000068614.7	11464
Sytl2	0.89	0.009	protein_coding	ENSMUSG00000030616.15	83671
Gm13443	0.88	0.007	processed_pseudogene	ENSMUSG00000075391.6	NA
Trim43a	0.88	0.020	protein_coding	ENSMUSG00000090693.3	547109
Lmcd1	0.88	0.003	protein_coding	ENSMUSG00000057604.9	30937
Nab2	0.87	0.014	protein_coding	ENSMUSG00000025402.12	17937
H2-Q5	0.87	0.037	polymorphic_pseudogene	ENSMUSG00000055413.12	15016
A930018M24Rik	0.87	0.006	protein_coding	ENSMUSG00000091089.1	NA
BC023105	0.86	0.006	pseudogene	ENSMUSG00000063388.4	NA
A330074K22Rik	0.86	0.015	lincRNA	ENSMUSG00000097960.1	434353
Slc7a1	0.86	0.009	protein_coding	ENSMUSG00000041313.14	11987
Slc25a13	0.85	0.033	protein_coding	ENSMUSG00000015112.15	50799
Gm26574	0.85	0.035	antisense	ENSMUSG00000097925.1	NA
Sacs	0.85	0.003	protein_coding	ENSMUSG00000048279.17	50720
Spsb2	0.85	0.006	protein_coding	ENSMUSG00000038451.13	14794
Ephb1	0.85	0.049	protein_coding	ENSMUSG00000032537.14	270190
Ptprq	0.84	0.036	protein_coding	ENSMUSG00000035916.7	237523
Dynlt1c	0.84	0.017	protein_coding	ENSMUSG00000000579.14	100040563
Gm13943	0.84	0.006	lincRNA	ENSMUSG00000085277.1	NA
Nudt5	0.84	0.005	protein_coding	ENSMUSG00000025817.12	53893
Cxx1b	0.83	0.001	protein_coding	ENSMUSG00000067924.4	553127
C130036L24Rik	0.83	0.012	processed_transcript	ENSMUSG00000073627.7	NA
Tspan13	0.83	0.001	protein_coding	ENSMUSG00000020577.15	66109
Egf	0.83	0.001	protein_coding	ENSMUSG00000028017.7	13645
Gm6368	0.83	0.043	processed_pseudogene	ENSMUSG00000080775.1	NA
Parp14	0.82	0.002	protein_coding	ENSMUSG00000034422.14	547253
Abra	0.82	0.011	protein_coding	ENSMUSG00000042895.5	223513
Nxpe3	0.82	0.048	protein_coding	ENSMUSG00000075033.3	385658
Tbx6	0.82	0.037	protein_coding	ENSMUSG00000030699.16	21389
Slc25a34	0.82	0.047	protein_coding	ENSMUSG00000040740.7	384071
Gm9920	0.80	0.048	protein_coding	ENSMUSG00000053749.12	100503794
Sept1	0.80	0.010	protein_coding	ENSMUSG00000000486.13	54204
Ifih1	0.80	0.003	protein_coding	ENSMUSG00000026896.14	71586
Gpt	0.80	0.002	protein_coding	ENSMUSG00000022546.4	76282
Prrxl1	0.80	0.041	protein_coding	ENSMUSG00000041730.13	107751
Gm42972	0.80	0.040	TEC	ENSMUSG00000105445.1	NA
Rhou	0.80	0.025	protein_coding	ENSMUSG00000039960.5	69581
Arhgef2	0.79	0.000	protein_coding	ENSMUSG00000028059.15	16800
Pank1	0.79	0.025	protein_coding	ENSMUSG00000033610.15	75735
Aspa	0.79	0.008	protein_coding	ENSMUSG00000020774.9	11484
Rsad2	0.79	0.049	protein_coding	ENSMUSG00000020641.15	58185
Gm42973	0.78	0.046	TEC	ENSMUSG00000105733.1	NA
Got1	0.78	0.001	protein_coding	ENSMUSG00000025190.13	14718
Galnt7	0.77	0.006	protein_coding	ENSMUSG00000031608.13	108150
Obsl1	0.77	0.012	protein_coding	ENSMUSG00000026211.17	98733
Prkg1	0.77	0.023	protein_coding	ENSMUSG00000052920.14	19091
Gm10524	0.76	0.045	TEC	ENSMUSG00000097141.2	NA
Tal2	0.76	0.038	protein_coding	ENSMUSG00000028417.3	21350
Hmga1-rs1	0.76	0.018	protein_coding	ENSMUSG00000078249.5	111241
Ift27	0.76	0.033	protein_coding	ENSMUSG00000016637.6	67042
Ckmt2	0.75	0.009	protein_coding	ENSMUSG00000021622.3	76722
P2ry1	0.75	0.017	protein_coding	ENSMUSG00000027765.6	18441
Perm1	0.75	0.001	protein_coding	ENSMUSG00000078486.3	74183
2210011C24Rik	0.75	0.013	protein_coding	ENSMUSG00000074217.8	70134
Myog	0.75	0.050	protein_coding	ENSMUSG00000026459.5	17928
Ifit1	0.75	0.035	protein_coding	ENSMUSG00000034459.8	15957
Trp63	0.74	0.001	protein_coding	ENSMUSG00000022510.14	22061
Slc16a1	0.74	0.003	protein_coding	ENSMUSG00000032902.1	20501
Esrrg	0.74	0.033	protein_coding	ENSMUSG00000026610.13	26381
Apobec2	0.74	0.002	protein_coding	ENSMUSG00000040694.3	11811
Gm16216	0.74	0.036	processed_pseudogene	ENSMUSG00000082107.1	NA
Fam83h	0.73	0.012	protein_coding	ENSMUSG00000046761.12	105732
2810032G03Rik	0.73	0.048	lincRNA	ENSMUSG00000037735.7	72669
Zfp870	0.73	0.028	protein_coding	ENSMUSG00000095325.1	240066
Kdr	0.73	0.019	protein_coding	ENSMUSG00000062960.10	16542
Btnl9	0.73	0.032	protein_coding	ENSMUSG00000040283.14	237754
Rad9b	0.72	0.028	protein_coding	ENSMUSG00000038569.13	231724
Gmnn	0.72	0.016	protein_coding	ENSMUSG00000006715.11	57441
Mvd	0.72	0.027	protein_coding	ENSMUSG00000006517.5	192156
Coq8a	0.72	0.001	protein_coding	ENSMUSG00000026489.13	67426
Gm26628	0.72	0.048	antisense	ENSMUSG00000097133.1	102632638
Tbc1d4	0.72	0.015	protein_coding	ENSMUSG00000033083.16	210789
1110020A21Rik	0.72	0.043	lincRNA	ENSMUSG00000097047.1	68531
Habp2	0.72	0.026	protein_coding	ENSMUSG00000025075.14	226243
Evc	0.72	0.005	protein_coding	ENSMUSG00000029122.11	59056
Tmem47	0.71	0.010	protein_coding	ENSMUSG00000025666.16	192216
Gm6736	0.71	0.033	processed_pseudogene	ENSMUSG00000071414.4	NA
Fam69b	0.71	0.033	protein_coding	ENSMUSG00000036186.5	56279
Snn	0.70	0.027	protein_coding	ENSMUSG00000037972.6	20621
Plet1	0.70	0.038	protein_coding	ENSMUSG00000032068.14	76509
Amot	0.70	0.001	protein_coding	ENSMUSG00000041688.16	27494
Gm10390	0.70	0.022	antisense	ENSMUSG00000097368.2	100503427
Deptor	0.70	0.017	protein_coding	ENSMUSG00000022419.15	97998
Stc2	0.70	0.042	protein_coding	ENSMUSG00000020303.2	20856
Cdkl3	0.70	0.005	protein_coding	ENSMUSG00000020389.19	213084
Ptpn14	0.70	0.002	protein_coding	ENSMUSG00000026604.17	19250
Gm35339	0.70	0.026	protein_coding	ENSMUSG00000109179.1	102638882
Ache	0.70	0.013	protein_coding	ENSMUSG00000023328.14	11423
Bche	0.69	0.043	protein_coding	ENSMUSG00000027792.11	12038
Ppm1k	0.69	0.001	protein_coding	ENSMUSG00000037826.5	243382
Lrrc74b	0.69	0.025	protein_coding	ENSMUSG00000022759.13	74685
Gm15545	0.69	0.033	antisense	ENSMUSG00000087138.1	100502630
Stbd1	0.69	0.039	protein_coding	ENSMUSG00000047963.7	52331
Kctd1	0.69	0.047	protein_coding	ENSMUSG00000036225.14	106931

Fxyd2	-1.18	0.030	protein_coding	ENSMUSG00000059412.6	11936
Macrod2os1	-1.18	0.014	antisense	ENSMUSG00000086450.1	NA
Xpr1	-1.16	0.001	protein_coding	ENSMUSG00000026469.14	19775
Styx	-1.16	0.000	protein_coding	ENSMUSG00000053205.8	56291
Nos1ap	-1.16	0.035	protein_coding	ENSMUSG00000038473.14	70729
Tpbgl	-1.15	0.002	protein_coding	ENSMUSG00000096606.2	100503386
Lrtm1	-1.14	0.016	protein_coding	ENSMUSG00000045776.2	319476
Whrn	-1.14	0.014	protein_coding	ENSMUSG00000039137.18	73750
Cdk19	-1.14	0.002	protein_coding	ENSMUSG00000038481.12	78334
4930556M19Rik	-1.14	0.005	lincRNA	ENSMUSG00000096971.1	75259
Gm17218	-1.14	0.011	antisense	ENSMUSG00000091078.1	NA
Wnt4	-1.13	0.002	protein_coding	ENSMUSG00000036856.4	22417
Gm5865	-1.12	0.006	processed_pseudogene	ENSMUSG00000104923.1	NA
Spred3	-1.11	0.002	protein_coding	ENSMUSG00000037239.8	101809
Mylk2	-1.10	0.001	protein_coding	ENSMUSG00000027470.9	228785
Tnc	-1.10	0.001	protein_coding	ENSMUSG00000028364.15	21923
Sema3a	-1.09	0.006	protein_coding	ENSMUSG00000028883.17	20346
Mfap3l	-1.09	0.003	protein_coding	ENSMUSG00000031647.10	71306
Ngf	-1.08	0.034	protein_coding	ENSMUSG00000027859.10	18049
Comp	-1.08	0.038	protein_coding	ENSMUSG00000031849.9	12845
Stab2	-1.08	0.005	protein_coding	ENSMUSG00000035459.15	192188
Fmod	-1.08	0.002	protein_coding	ENSMUSG00000041559.7	14264
Cpeb1	-1.07	0.004	protein_coding	ENSMUSG00000025586.17	12877
Spns2	-1.07	0.000	protein_coding	ENSMUSG00000040447.15	216892
Bcl2	-1.06	0.001	protein_coding	ENSMUSG00000057329.7	12043
Calm3	-1.06	0.000	protein_coding	ENSMUSG00000019370.10	12315
Wbscr17	-1.05	0.004	protein_coding	ENSMUSG00000034040.16	212996
Ccdc146	-1.04	0.041	protein_coding	ENSMUSG00000064280.13	75172
Smarca1	-1.04	0.038	protein_coding	ENSMUSG00000031099.16	93761
AI854703	-1.03	0.004	protein_coding	ENSMUSG00000053297.14	NA
Stim2	-1.03	0.000	protein_coding	ENSMUSG00000039156.19	116873
Mc5r	-1.02	0.049	protein_coding	ENSMUSG00000007480.3	17203
Xylt1	-1.02	0.002	protein_coding	ENSMUSG00000030657.11	233781
Shisa3	-1.02	0.023	protein_coding	ENSMUSG00000050010.8	330096
Pde4b	-1.02	0.003	protein_coding	ENSMUSG00000028525.16	18578
Tspyl5	-1.01	0.013	protein_coding	ENSMUSG00000038984.3	239364
Actr3b	-1.01	0.000	protein_coding	ENSMUSG00000056367.14	242894
Npnt	-1.01	0.031	protein_coding	ENSMUSG00000040998.18	114249
Net1	-1.01	0.000	protein_coding	ENSMUSG00000021215.14	56349
Fam78a	-1.01	0.005	protein_coding	ENSMUSG00000050592.8	241303
Gm7694	-1.01	0.046	protein_coding	ENSMUSG00000102752.1	665574
Fras1	-1.00	0.036	protein_coding	ENSMUSG00000034687.8	231470
Lrrc55	-1.00	0.049	protein_coding	ENSMUSG00000075224.8	241528
Nkain1	-1.00	0.000	protein_coding	ENSMUSG00000078532.9	67149
Phex	-1.00	0.022	protein_coding	ENSMUSG00000057457.11	18675
Zfp52	-1.00	0.008	protein_coding	ENSMUSG00000051341.5	22710
Gm14680	-0.99	0.005	processed_pseudogene	ENSMUSG00000081752.3	NA
Calm1	-0.99	0.000	protein_coding	ENSMUSG00000001175.13	12313
Slc30a2	-0.98	0.031	protein_coding	ENSMUSG00000028836.14	230810
Phka1	-0.98	0.000	protein_coding	ENSMUSG00000034055.16	18679
Slc16a10	-0.98	0.001	protein_coding	ENSMUSG00000019838.10	72472
Gm36962	-0.97	0.007	TEC	ENSMUSG00000103931.1	NA
Gm10605	-0.97	0.023	lincRNA	ENSMUSG00000097974.1	NA
Gm5881	-0.97	0.008	processed_pseudogene	ENSMUSG00000107747.1	NA
Rnase13	-0.97	0.042	protein_coding	ENSMUSG00000068392.2	497071
Tmem233	-0.97	0.000	protein_coding	ENSMUSG00000079278.1	545798
Tifa	-0.96	0.042	protein_coding	ENSMUSG00000046688.14	211550
Tcte2	-0.96	0.017	protein_coding	ENSMUSG00000038347.14	21646
Smarcd3	-0.96	0.000	protein_coding	ENSMUSG00000028949.13	66993
Gm10118	-0.96	0.032	protein_coding	ENSMUSG00000062561.3	NA
Fam69a	-0.96	0.004	protein_coding	ENSMUSG00000029270.10	67266
Fbp2	-0.95	0.001	protein_coding	ENSMUSG00000021456.7	14120
D1Ertd622e	-0.95	0.004	protein_coding	ENSMUSG00000044768.16	52392
D430041D05Rik	-0.94	0.004	protein_coding	ENSMUSG00000068373.14	241589
Gm11451	-0.94	0.035	processed_pseudogene	ENSMUSG00000083567.3	NA
Wnk3	-0.94	0.004	protein_coding	ENSMUSG00000041245.13	279561
Tppp	-0.93	0.003	protein_coding	ENSMUSG00000021573.14	72948
Nek5	-0.93	0.045	protein_coding	ENSMUSG00000037738.10	330721
Adgre1	-0.93	0.041	protein_coding	ENSMUSG00000004730.14	13733
Nmbr	-0.93	0.039	protein_coding	ENSMUSG00000019865.9	18101
Gpr155	-0.93	0.000	protein_coding	ENSMUSG00000041762.16	68526
Map9	-0.92	0.030	protein_coding	ENSMUSG00000033900.13	213582
Ccdc6	-0.92	0.000	protein_coding	ENSMUSG00000048701.13	76551
Vasp	-0.92	0.003	protein_coding	ENSMUSG00000030403.9	22323
Gm15820	-0.92	0.033	processed_pseudogene	ENSMUSG00000107105.1	NA
Hoxa11	-0.92	0.040	protein_coding	ENSMUSG00000038210.10	15396
Gm15511	-0.91	0.037	antisense	ENSMUSG00000086648.1	102642202
Slc38a4	-0.91	0.005	protein_coding	ENSMUSG00000022464.13	69354
Map2k6	-0.91	0.002	protein_coding	ENSMUSG00000020623.11	26399
D930007P13Rik	-0.90	0.050	lincRNA	ENSMUSG00000097003.1	432989
Fgf7	-0.90	0.017	protein_coding	ENSMUSG00000027208.14	14178
Aebp1	-0.90	0.029	protein_coding	ENSMUSG00000020473.13	11568
Ptgfr	-0.90	0.048	protein_coding	ENSMUSG00000028036.6	19220
Scamp5	-0.89	0.033	protein_coding	ENSMUSG00000040722.6	56807
Pdgfc	-0.89	0.043	protein_coding	ENSMUSG00000028019.9	54635
Itga4	-0.89	0.000	protein_coding	ENSMUSG00000027009.18	16401
Slc37a4	-0.89	0.010	protein_coding	ENSMUSG00000032114.8	14385
Col11a1	-0.89	0.012	protein_coding	ENSMUSG00000027966.20	12814
Il1rl1	-0.88	0.036	protein_coding	ENSMUSG00000026069.15	17082
Fam189b	-0.88	0.007	protein_coding	ENSMUSG00000032657.15	68521
Gcnt2	-0.88	0.006	protein_coding	ENSMUSG00000021360.15	14538
Vit	-0.88	0.030	protein_coding	ENSMUSG00000024076.9	74199
Col8a2	-0.88	0.046	protein_coding	ENSMUSG00000056174.8	329941
Polk	-0.87	0.000	protein_coding	ENSMUSG00000021668.14	27015
Tbx18	-0.87	0.037	protein_coding	ENSMUSG00000032419.8	76365
Odc1	-0.87	0.024	protein_coding	ENSMUSG00000011179.7	18263
Gm38393	-0.87	0.003	protein_coding	ENSMUSG00000000948.16	NA
Fibin	-0.87	0.012	protein_coding	ENSMUSG00000074971.4	67606
Mylk4	-0.86	0.004	protein_coding	ENSMUSG00000044951.13	238564
Tigd4	-0.86	0.009	protein_coding	ENSMUSG00000047819.3	403175
Chodl	-0.86	0.049	protein_coding	ENSMUSG00000022860.14	246048
Tfcp2l1	-0.86	0.016	protein_coding	ENSMUSG00000026380.10	81879
Auts2	-0.85	0.008	protein_coding	ENSMUSG00000029673.17	319974
Srcin1	-0.85	0.004	protein_coding	ENSMUSG00000038453.16	56013
Tdrkh	-0.85	0.005	protein_coding	ENSMUSG00000041912.12	72634
Zfp467	-0.85	0.003	protein_coding	ENSMUSG00000068551.12	68910
Mpp3	-0.85	0.031	protein_coding	ENSMUSG00000052373.14	13384
Aph1c	-0.84	0.047	protein_coding	ENSMUSG00000053040.13	68318
Smtnl2	-0.84	0.005	protein_coding	ENSMUSG00000045667.14	276829
Sms	-0.84	0.003	protein_coding	ENSMUSG00000071708.11	20603
Adgrl1	-0.83	0.007	protein_coding	ENSMUSG00000013033.16	330814
Hoxa13	-0.83	0.013	protein_coding	ENSMUSG00000038203.18	15398
Pdlim7	-0.83	0.000	protein_coding	ENSMUSG00000021493.15	67399
Klf2	-0.83	0.045	protein_coding	ENSMUSG00000055148.7	16598
Ccdc50	-0.82	0.000	protein_coding	ENSMUSG00000038127.14	67501
mt-Tp	-0.82	0.001	Mt_tRNA	ENSMUSG00000064372.1	NA
Ppp1r14c	-0.82	0.001	protein_coding	ENSMUSG00000040653.5	76142
Ccnyl1	-0.82	0.003	protein_coding	ENSMUSG00000070871.10	227210
Rnf126	-0.82	0.003	protein_coding	ENSMUSG00000035890.8	70294
Gnao1	-0.82	0.002	protein_coding	ENSMUSG00000031748.16	14681
Arnt2	-0.82	0.048	protein_coding	ENSMUSG00000015709.9	11864
Ybx3	-0.81	0.000	protein_coding	ENSMUSG00000030189.15	56449
Kmt5a	-0.80	0.001	protein_coding	ENSMUSG00000049327.17	67956
Gm43268	-0.80	0.027	TEC	ENSMUSG00000107182.1	NA
Mtss1l	-0.80	0.004	protein_coding	ENSMUSG00000033763.14	244654
Zfp277	-0.80	0.017	protein_coding	ENSMUSG00000055917.14	246196
Myh4	-0.80	0.003	protein_coding	ENSMUSG00000057003.12	17884
Hacd1	-0.80	0.001	protein_coding	ENSMUSG00000063275.15	30963
Cd109	-0.80	0.013	protein_coding	ENSMUSG00000046186.8	235505
Gnpnat1	-0.80	0.001	protein_coding	ENSMUSG00000037722.7	54342
Cys1	-0.79	0.048	protein_coding	ENSMUSG00000062563.14	12879
Acvr1b	-0.79	0.005	protein_coding	ENSMUSG00000000532.10	11479
Pde7a	-0.79	0.002	protein_coding	ENSMUSG00000069094.12	18583
Gid4	-0.78	0.000	protein_coding	ENSMUSG00000018415.14	66771
Gm43946	-0.78	0.009	TEC	ENSMUSG00000107720.1	NA
Plekhb1	-0.78	0.004	protein_coding	ENSMUSG00000030701.17	27276
Gm14326	-0.78	0.005	protein_coding	ENSMUSG00000078862.10	665211
Cpeb2	-0.77	0.002	protein_coding	ENSMUSG00000039782.14	231207
Sap30	-0.77	0.026	protein_coding	ENSMUSG00000031609.3	60406
Igfbp5	-0.77	0.013	protein_coding	ENSMUSG00000026185.8	16011
Msrb3	-0.77	0.000	protein_coding	ENSMUSG00000051236.13	320183
Gprc5b	-0.76	0.000	protein_coding	ENSMUSG00000008734.9	64297
Gm15943	-0.76	0.046	processed_transcript	ENSMUSG00000086801.1	NA
Syt8	-0.76	0.046	protein_coding	ENSMUSG00000031098.18	55925
Gm43807	-0.76	0.049	sense_intronic	ENSMUSG00000107048.1	NA
Col12a1	-0.76	0.007	protein_coding	ENSMUSG00000032332.17	12816

bioRxiv preprint doi: https://doi.org/10.1101/2023.01.30.526183; this version posted January 31, 2023. The copyright holder for this preprint (which was not certified by peer review) is the author/funder, who has granted bioRxiv a license to display the preprint in perpetuity. It is made available under aCC-BY 4.0 International license.

Gm5113	0.69	0.004	protein_coding	ENSMUSG00000066647.6	330503	*Fermt2*	-0.75	0.000	protein_coding	ENSMUSG00000037712.15	218952
B9d2	0.68	0.027	protein_coding	ENSMUSG00000063439.7	232987	*Slc25a30*	-0.75	0.027	protein_coding	ENSMUSG00000022003.6	67554
Nlrc5	0.68	0.040	protein_coding	ENSMUSG00000074151.13	434341	*Hook1*	-0.75	0.004	protein_coding	ENSMUSG00000028572.13	77963
Iqcg	0.68	0.024	protein_coding	ENSMUSG00000035578.15	69707	*Gas1*	-0.75	0.002	protein_coding	ENSMUSG00000052957.7	14451
Gm7967	0.68	0.036	lincRNA	ENSMUSG00000100009.6	NA	*Bmp2*	-0.75	0.018	protein_coding	ENSMUSG00000027358.6	12156
Cdc14a	0.67	0.041	protein_coding	ENSMUSG00000033502.14	229776	*Dennd2c*	-0.75	0.013	protein_coding	ENSMUSG00000007379.15	329727
Parp10	0.67	0.009	protein_coding	ENSMUSG00000063268.12	671535	*Mab21l1*	-0.75	0.039	protein_coding	ENSMUSG00000056947.5	17116
Tm6sf1	0.67	0.033	protein_coding	ENSMUSG00000038623.9	107769	*Tspan8*	-0.75	0.012	protein_coding	ENSMUSG00000034127.14	216350
Fam13a	0.67	0.012	protein_coding	ENSMUSG00000037709.13	58909	*Mrln*	-0.74	0.012	processed_transcript	ENSMUSG00000019933.7	69563
Evc2	0.67	0.001	protein_coding	ENSMUSG00000050248.11	68525	*Plekho1*	-0.74	0.002	protein_coding	ENSMUSG00000015745.9	67220
Rfx5	0.66	0.005	protein_coding	ENSMUSG00000005774.12	53970	*Gm22362*	-0.74	0.037	snoRNA	ENSMUSG00000065036.1	115486329
Ldhd	0.66	0.006	protein_coding	ENSMUSG00000031958.16	52815	*Prkab2*	-0.73	0.004	protein_coding	ENSMUSG00000038205.12	108097
App	0.66	0.008	protein_coding	ENSMUSG00000022892.10	11820	*Homer1*	-0.73	0.000	protein_coding	ENSMUSG00000007617.17	26556
Ltbp1	0.65	0.004	protein_coding	ENSMUSG00000001870.15	268977	*Rhobtb1*	-0.73	0.012	protein_coding	ENSMUSG00000019944.14	69288
Fam110a	0.65	0.031	protein_coding	ENSMUSG00000027459.16	73847	*Eya1*	-0.73	0.000	protein_coding	ENSMUSG00000025932.14	14048
Plet1os	0.65	0.024	antisense	ENSMUSG00000101304.1	69631	*Six2*	-0.73	0.016	protein_coding	ENSMUSG00000024134.11	20472
2900052L18Rik	0.65	0.009	processed_transcript	ENSMUSG00000043993.6	NA	*Anxa1*	-0.73	0.046	protein_coding	ENSMUSG00000024659.14	16952
Flnc	0.65	0.014	protein_coding	ENSMUSG00000068699.12	68794	*Osbpl6*	-0.73	0.001	protein_coding	ENSMUSG00000042359.18	99031
Ifi35	0.65	0.013	protein_coding	ENSMUSG00000010358.13	70110	*Shtn1*	-0.73	0.045	protein_coding	ENSMUSG00000041362.16	71653
H2-T24	0.65	0.012	protein_coding	ENSMUSG00000053835.17	15042	*Capg*	-0.73	0.015	protein_coding	ENSMUSG00000056737.14	12332
Klhl33	0.64	0.013	protein_coding	ENSMUSG00000090799.1	546611	*Gm10762*	-0.73	0.034	protein_coding	ENSMUSG00000074807.2	NA
Tpm2	0.64	0.003	protein_coding	ENSMUSG00000028464.16	22004	*Nudt3*	-0.72	0.000	protein_coding	ENSMUSG00000024213.13	56409
AW011738	0.64	0.021	protein_coding	ENSMUSG00000078349.2	100382	*Gfer*	-0.72	0.009	protein_coding	ENSMUSG00000040888.9	11692
Sesn3	0.64	0.004	protein_coding	ENSMUSG00000032009.8	75747	*Prkcd*	-0.72	0.008	protein_coding	ENSMUSG00000021948.15	18753
Pex6	0.64	0.017	protein_coding	ENSMUSG00000002763.16	224824	*Cd24a*	-0.72	0.010	protein_coding	ENSMUSG00000047139.8	12484
Cyp4f13	0.64	0.035	protein_coding	ENSMUSG00000024055.14	170716	*Tgif1*	-0.72	0.017	protein_coding	ENSMUSG00000047407.17	21815
Chrnb1	0.64	0.006	protein_coding	ENSMUSG00000041189.9	11443	*Dagla*	-0.71	0.005	protein_coding	ENSMUSG00000035735.10	269060
Hspb1	0.64	0.010	protein_coding	ENSMUSG00000004951.10	15507	*Gpt2*	-0.71	0.002	protein_coding	ENSMUSG00000031700.11	108682
Mpc1-ps	0.64	0.009	processed_pseudogene	ENSMUSG00000091498.4	NA	*E130308A19Rik*	-0.71	0.038	protein_coding	ENSMUSG00000045071.13	230259
Cox6a2	0.64	0.002	protein_coding	ENSMUSG00000030785.8	12862	*Efemp1*	-0.71	0.016	protein_coding	ENSMUSG00000020467.15	216616
Fam73b	0.64	0.050	protein_coding	ENSMUSG00000026858.18	108958	*Musk*	-0.71	0.015	protein_coding	ENSMUSG00000057280.15	18198
4930513N20Rik	0.63	0.007	TEC	ENSMUSG00000109225.1	NA	*Slc10a7*	-0.71	0.002	protein_coding	ENSMUSG00000031684.11	76775
Gm15337	0.63	0.045	antisense	ENSMUSG00000085289.1	NA	*Tdrp*	-0.70	0.048	protein_coding	ENSMUSG00000050052.12	72148
Gm11613	0.63	0.006	processed_transcript	ENSMUSG00000085586.1	NA	*Samd1*	-0.70	0.033	protein_coding	ENSMUSG00000079003.2	666704
Pnpla2	0.63	0.012	protein_coding	ENSMUSG00000025509.15	66853	*Tab2*	-0.70	0.000	protein_coding	ENSMUSG00000015755.16	68652
4632428C04Rik	0.63	0.047	lincRNA	ENSMUSG00000097184.1	100043102	*Efhd1*	-0.70	0.042	protein_coding	ENSMUSG00000026255.15	98363
Irs1	0.63	0.011	protein_coding	ENSMUSG00000055980.2	16367	*Vps72*	-0.69	0.001	protein_coding	ENSMUSG00000008958.14	21427
Gm17197	0.63	0.013	antisense	ENSMUSG00000090990.1	NA	*Ppp1r14b*	-0.69	0.002	protein_coding	ENSMUSG00000056612.6	18938
Plekhn1	0.63	0.038	protein_coding	ENSMUSG00000078485.2	231002	*Ociad2*	-0.69	0.039	protein_coding	ENSMUSG00000029153.11	433904
Zfp827	0.63	0.029	protein_coding	ENSMUSG00000071064.13	622675	*Ldlrad4*	-0.69	0.010	protein_coding	ENSMUSG00000024544.8	52662
Arhgap4	0.62	0.036	protein_coding	ENSMUSG00000031389.17	171207	*Cmya5*	-0.68	0.000	protein_coding	ENSMUSG00000047419.5	76469
D330041H03Rik	0.62	0.017	processed_transcript	ENSMUSG00000073437.10	654822	*Klf4*	-0.68	0.030	protein_coding	ENSMUSG00000003032.8	16600
Uck1	0.62	0.039	protein_coding	ENSMUSG00000002550.16	22245	*Rxrg*	-0.68	0.005	protein_coding	ENSMUSG00000015843.10	20183
Runx1	0.62	0.023	protein_coding	ENSMUSG00000022952.16	12394	*RP24-343P7.1*	-0.68	0.018	processed_pseudogene	ENSMUSG00000110644.1	NA
Fahd1	0.62	0.004	protein_coding	ENSMUSG00000045316.4	68636	*Ddit4l*	-0.68	0.003	protein_coding	ENSMUSG00000046818.7	73284
Gm43162	0.62	0.027	TEC	ENSMUSG00000104724.1	NA	*9530086O07Rik*	-0.68	0.003	TEC	ENSMUSG00000108076.1	NA
Gm38344	0.62	0.024	TEC	ENSMUSG00000102452.1	NA	*Phf7*	-0.68	0.004	protein_coding	ENSMUSG00000021902.6	71838
Gm37953	0.62	0.005	TEC	ENSMUSG00000104061.1	NA	*Ppp1r3c*	-0.68	0.038	protein_coding	ENSMUSG00000067279.2	53412
Tpra1	0.62	0.032	protein_coding	ENSMUSG00000002871.14	24100	*Zfp788*	-0.67	0.019	protein_coding	ENSMUSG00000074165.10	67607
Fer1l6	0.62	0.010	protein_coding	ENSMUSG00000037106.8	631797	*Snrpn*	-0.67	0.003	protein_coding	ENSMUSG00000102252.5	20646
Fdxr	0.62	0.011	protein_coding	ENSMUSG00000018861.8	14149	*Bhlhe40*	-0.67	0.031	protein_coding	ENSMUSG00000030103.11	20893
Dmpk	0.62	0.002	protein_coding	ENSMUSG00000030409.15	13400	*Tmod2*	-0.67	0.004	protein_coding	ENSMUSG00000032186.14	50876
Lmod3	0.62	0.010	protein_coding	ENSMUSG00000044086.8	320502	*Mllt11*	-0.66	0.002	protein_coding	ENSMUSG00000053192.9	56772
Mettl21a	0.61	0.004	protein_coding	ENSMUSG00000025956.10	67099	*Usp6nl*	-0.66	0.010	protein_coding	ENSMUSG00000039046.15	98910
Trim7	0.61	0.002	protein_coding	ENSMUSG00000040350.15	94089	*Hmga1*	-0.65	0.023	protein_coding	ENSMUSG00000046711.15	15361
Cdc42ep2	0.61	0.003	protein_coding	ENSMUSG00000045664.4	104252	*Fgf2*	-0.65	0.033	protein_coding	ENSMUSG00000037225.13	14173
Stat1	0.61	0.012	protein_coding	ENSMUSG00000026104.14	20846	*Reep1*	-0.65	0.000	protein_coding	ENSMUSG00000052852.8	52250
Abr	0.61	0.002	protein_coding	ENSMUSG00000017631.18	109934	*Gm42651*	-0.65	0.047	TEC	ENSMUSG00000104877.1	NA
Vars2	0.60	0.002	protein_coding	ENSMUSG00000038838.15	68915	*Gm43953*	-0.65	0.011	TEC	ENSMUSG00000108067.1	NA
Klf5	0.60	0.025	protein_coding	ENSMUSG00000005148.7	12224	*Ankrd44*	-0.65	0.007	protein_coding	ENSMUSG00000052331.14	329154
Ppif	0.60	0.009	protein_coding	ENSMUSG00000021868.6	105675	*Eps15*	-0.65	0.000	protein_coding	ENSMUSG00000028552.13	13858
Cntrob	0.60	0.039	protein_coding	ENSMUSG00000032782.19	216846	*Rsph3b*	-0.65	0.029	protein_coding	ENSMUSG00000023806.9	100037282
Fut2	0.60	0.049	protein_coding	ENSMUSG00000055978.5	14344	*Tnfrsf21*	-0.65	0.021	protein_coding	ENSMUSG00000023915.4	94185
B3galt1	0.60	0.024	protein_coding	ENSMUSG00000034780.6	26877	*Fhl3*	-0.65	0.010	protein_coding	ENSMUSG00000032643.12	14201
Fv1	0.60	0.042	protein_coding	ENSMUSG00000070583.1	14349	*Mcm3*	-0.65	0.042	protein_coding	ENSMUSG00000041859.10	17215
Ndc1	0.60	0.002	protein_coding	ENSMUSG00000028614.14	72787	*5330439B14Rik*	-0.65	0.050	antisense	ENSMUSG00000104234.1	321015
Calr3	0.60	0.037	protein_coding	ENSMUSG00000019732.14	73316	*Gm26911*	-0.64	0.037	antisense	ENSMUSG00000097834.2	NA
Neat1	0.59	0.001	lincRNA	ENSMUSG00000092274.2	66961	*Pcdhga11*	-0.64	0.030	protein_coding	ENSMUSG00000102742.1	93723
Acsl1	0.59	0.030	protein_coding	ENSMUSG00000018796.13	14081	*Ubald2*	-0.64	0.008	protein_coding	ENSMUSG00000050628.6	319370
Ttc7	0.59	0.004	protein_coding	ENSMUSG00000036918.15	225049	*Srebf1*	-0.64	0.040	protein_coding	ENSMUSG00000020538.15	20787
Adam1a	0.59	0.046	protein_coding	ENSMUSG00000072647.5	280668	*Otub2*	-0.64	0.007	protein_coding	ENSMUSG00000021203.15	68149
Acot9	0.59	0.009	protein_coding	ENSMUSG00000025287.15	56360	*Csde1*	-0.64	0.000	protein_coding	ENSMUSG00000068823.10	229663
Tnfsf10	0.58	0.024	protein_coding	ENSMUSG00000039304.11	22035	*Abca1*	-0.64	0.009	protein_coding	ENSMUSG00000015243.4	11303
Nme1	0.58	0.004	protein_coding	ENSMUSG00000037601.11	18102	*Hrasls*	-0.64	0.006	protein_coding	ENSMUSG00000022525.13	27281
Cst6	0.58	0.025	protein_coding	ENSMUSG00000024846.5	73720	*Npc1*	-0.64	0.001	protein_coding	ENSMUSG00000024413.13	18145
Rab20	0.58	0.044	protein_coding	ENSMUSG00000031504.6	19332	*Ldha-ps2*	-0.64	0.013	processed_pseudogene	ENSMUSG00000083836.1	NA
Gm42507	0.58	0.035	TEC	ENSMUSG00000107168.1	NA	*Rora*	-0.64	0.001	protein_coding	ENSMUSG00000032238.17	19883
Rnf122	0.58	0.011	protein_coding	ENSMUSG00000039328.10	68867	*Dyrk2*	-0.64	0.017	protein_coding	ENSMUSG00000028630.8	69181
Gtpbp6	0.58	0.035	protein_coding	ENSMUSG00000033434.15	107999	*Fam122a*	-0.63	0.008	protein_coding	ENSMUSG00000074922.1	68034
Art5	0.58	0.010	protein_coding	ENSMUSG00000070424.12	11875	*Ammecr1*	-0.63	0.011	protein_coding	ENSMUSG00000042225.3	56068
Tldc1	0.58	0.020	protein_coding	ENSMUSG00000034105.9	74347	*Vldlr*	-0.63	0.040	protein_coding	ENSMUSG00000024924.14	22359
Gm11361	0.58	0.024	processed_pseudogene	ENSMUSG00000061330.7	NA	*Maoa*	-0.63	0.044	protein_coding	ENSMUSG00000025037.6	17161
L3mbtl3	0.57	0.003	protein_coding	ENSMUSG00000039089.14	237339	*Plcd4*	-0.62	0.025	protein_coding	ENSMUSG00000026173.15	18802
Cars	0.57	0.001	protein_coding	ENSMUSG00000010755.17	27267	*Gm7332*	-0.62	0.043	processed_pseudogene	ENSMUSG00000080875.4	NA
Atrnl1	0.57	0.003	protein_coding	ENSMUSG00000054843.8	226255	*Rab12*	-0.62	0.001	protein_coding	ENSMUSG00000023460.12	19328
Hspb6	0.57	0.029	protein_coding	ENSMUSG00000036854.14	243912	*Smad7*	-0.62	0.007	protein_coding	ENSMUSG00000025880.11	17131
Slc25a22	0.57	0.047	protein_coding	ENSMUSG00000019082.18	68267	*Cbfb*	-0.62	0.004	protein_coding	ENSMUSG00000031885.14	12400
H2-T23	0.56	0.021	protein_coding	ENSMUSG00000067212.8	15040	*Jak2*	-0.62	0.018	protein_coding	ENSMUSG00000024789.12	16452
Cenpq	0.56	0.027	protein_coding	ENSMUSG00000023919.7	83815	*Gm43947*	-0.62	0.002	TEC	ENSMUSG00000107448.1	NA
Trappc12	0.56	0.008	protein_coding	ENSMUSG00000020628.15	217449	*Rai14*	-0.61	0.030	protein_coding	ENSMUSG00000022246.13	75646
Etv5	0.56	0.014	protein_coding	ENSMUSG00000013089.15	104156	*Optn*	-0.61	0.002	protein_coding	ENSMUSG00000026672.11	71648
Prepl	0.56	0.046	protein_coding	ENSMUSG00000024127.14	213760	*Tmcc1*	-0.61	0.001	protein_coding	ENSMUSG00000030126.17	330401
Sh3bgrl3	0.56	0.032	protein_coding	ENSMUSG00000028843.8	73723	*Mylip*	-0.61	0.007	protein_coding	ENSMUSG00000038175.9	218203
Cdc123	0.56	0.006	protein_coding	ENSMUSG00000039128.13	98828	*Dbn1*	-0.61	0.030	protein_coding	ENSMUSG00000034675.17	56320
Dtx3l	0.56	0.003	protein_coding	ENSMUSG00000049502.16	209200	*Lrrn4cl*	-0.61	0.006	protein_coding	ENSMUSG00000071656.6	68852
Ivd	0.55	0.009	protein_coding	ENSMUSG00000027332.11	56357	*Phospho1*	-0.61	0.020	protein_coding	ENSMUSG00000050860.6	237928
Pitx2	0.55	0.022	protein_coding	ENSMUSG00000028023.16	18741	*C2cd2l*	-0.61	0.004	protein_coding	ENSMUSG00000032120.10	71764
Got2	0.55	0.007	protein_coding	ENSMUSG00000031672.8	14719	*Gpd2*	-0.60	0.009	protein_coding	ENSMUSG00000026827.12	14571
Tm7sf2	0.55	0.049	protein_coding	ENSMUSG00000024799.14	73166	*Fmnl2*	-0.60	0.018	protein_coding	ENSMUSG00000036053.16	71409
Fads6	0.55	0.036	protein_coding	ENSMUSG00000044788.10	328035	*Lbx1*	-0.60	0.014	protein_coding	ENSMUSG00000025216.9	16814
Exog	0.55	0.047	protein_coding	ENSMUSG00000042787.13	208194	*Tenm3*	-0.60	0.031	protein_coding	ENSMUSG00000031561.16	23965
Gca	0.55	0.046	protein_coding	ENSMUSG00000026893.4	227960	*Lysmd2*	-0.60	0.033	protein_coding	ENSMUSG00000032184.4	70082
Slc4a3	0.54	0.050	protein_coding	ENSMUSG00000006576.16	20536	*Sub1*	-0.60	0.002	protein_coding	ENSMUSG00000022205.15	20024
Pdzd7	0.54	0.040	protein_coding	ENSMUSG00000074818.12	100503041	*Spin1*	-0.60	0.003	protein_coding	ENSMUSG00000021395.10	20729
Ddit3	0.54	0.013	protein_coding	ENSMUSG00000025408.15	13198	*Vezf1*	-0.60	0.001	protein_coding	ENSMUSG00000018377.10	22344
Ldlrap1	0.54	0.003	protein_coding	ENSMUSG00000037295.7	100017	*Gm28229*	-0.60	0.026	sense_intronic	ENSMUSG00000101335.1	NA
Crem	0.54	0.006	protein_coding	ENSMUSG00000063889.16	12916	*Pole4*	-0.60	0.031	protein_coding	ENSMUSG00000030042.15	66979
Rnf213	0.54	0.010	protein_coding	ENSMUSG00000070327.14	672511	*Tfdp1*	-0.60	0.002	protein_coding	ENSMUSG00000038482.11	21781
Lcmt2	0.53	0.027	protein_coding	ENSMUSG00000074890.4	329504	*Eef1akmt1*	-0.59	0.021	protein_coding	ENSMUSG00000021951.5	68043
Ppp1r11	0.53	0.018	protein_coding	ENSMUSG00000036398.13	76497	*Rab2a*	-0.59	0.004	protein_coding	ENSMUSG00000047187.9	59021
Myom2	0.53	0.003	protein_coding	ENSMUSG00000031461.4	17930	*Pctp*	-0.59	0.032	protein_coding	ENSMUSG00000020553.8	18559
Abcg2	0.53	0.009	protein_coding	ENSMUSG00000029802.13	26357	*Mical2*	-0.59	0.028	protein_coding	ENSMUSG00000038244.13	320878
Gm37314	0.53	0.007	TEC	ENSMUSG00000104094.1	NA	*Gatsl2*	-0.59	0.027	protein_coding	ENSMUSG00000015944.9	80909
Trim35	0.53	0.011	protein_coding	ENSMUSG00000022043.18	66854	*Skil*	-0.59	0.001	protein_coding	ENSMUSG00000027660.16	20482
Rnaseh2c	0.53	0.020	protein_coding	ENSMUSG00000024925.10	68209	*Tbkbp1*	-0.58	0.027	protein_coding	ENSMUSG00000038517.15	73174
RP23-425N12.2	0.52	0.046	lincRNA	ENSMUSG00000106981.1	NA	*Fmr1*	-0.58	0.049	protein_coding	ENSMUSG00000000838.17	14265
F11r	0.52	0.011	protein_coding	ENSMUSG00000038235.4	16456	*Fez2*	-0.58	0.006	protein_coding	ENSMUSG00000056121.15	225020
Eci1	0.52	0.019	protein_coding	ENSMUSG00000024132.5	13177	*Angptl7*	-0.58	0.013	protein_coding	ENSMUSG00000028989.3	654812
Arl6ip5	0.52	0.002	protein_coding	ENSMUSG00000035199.6	65106	*Tmem63a*	-0.58	0.001	protein_coding	ENSMUSG00000026519.16	208795
Ppara	0.52	0.033	protein_coding	ENSMUSG00000022383.13	19013	*Cpeb4*	-0.58	0.002	protein_coding	ENSMUSG00000020300.14	67579
Nudt8	0.52	0.024	protein_coding	ENSMUSG00000024869.9	NA	*Tada2a*	-0.58	0.031	protein_coding	ENSMUSG00000018651.14	217031
Abi2	0.52	0.005	protein_coding	ENSMUSG00000026782.15	329165	*Rxra*	-0.58	0.001	protein_coding	ENSMUSG00000015846.14	20181
Shmt2	0.52	0.009	protein_coding	ENSMUSG00000025403.4	108037	*Spire1*	-0.57	0.019	protein_coding	ENSMUSG00000024533.15	68166
Wbp1	0.52	0.005	protein_coding	ENSMUSG00000030035.14	22377	*2610203C20Rik*	-0.57	0.004	processed_transcript	ENSMUSG00000074415.11	73144
Usp54	0.52	0.045	protein_coding	ENSMUSG00000034235.16	78787	*Phc1*	-0.57	0.030	protein_coding	ENSMUSG00000040669.14	13619
Mrpl47	0.52	0.024	protein_coding	ENSMUSG00000037531.8	74600	*Slc26a6*	-0.57	0.021	protein_coding	ENSMUSG00000023259.16	171429
Kyat3	0.52	0.027	protein_coding	ENSMUSG00000040213.13	229905	*Zfp946*	-0.57	0.029	protein_coding	ENSMUSG00000071266.13	74149
Acss1	0.51	0.039	protein_coding	ENSMUSG00000027452.11	68738	*Ankrd6*	-0.57	0.026	protein_coding	ENSMUSG00000040183.13	140577
Dis3	0.51	0.004	protein_coding	ENSMUSG00000033166.10	72662	*Setbp1*	-0.57	0.001	protein_coding	ENSMUSG00000024548.11	240427
AA474408	0.51	0.029	TEC	ENSMUSG00000073867.2	NA	*Acvr1*	-0.57	0.014	protein_coding	ENSMUSG00000026836.15	11477
Camk2d	0.51	0.017	protein_coding	ENSMUSG00000053819.16	108058	*Eepd1*	-0.57	0.002	protein_coding	ENSMUSG00000036611.5	67484
Lmo7	0.51	0.004	protein_coding	ENSMUSG00000033060.15	380928	*Gm43672*	-0.57	0.010	lincRNA	ENSMUSG00000106019.1	NA
Oxnad1	0.51	0.009	protein_coding	ENSMUSG00000021906.13	218885	*Tnnt3*	-0.57	0.010	protein_coding	ENSMUSG00000061723.18	21957
Sars	0.51	0.002	protein_coding	ENSMUSG00000068739.13	20226	*Ttc7b*	-0.57	0.015	protein_coding	ENSMUSG00000033530.7	104718
Sec61a2	0.51	0.006	protein_coding	ENSMUSG00000025816.15	57743	*Htra1*	-0.57	0.004	protein_coding	ENSMUSG00000006205.13	56213
Parp12	0.51	0.011	protein_coding	ENSMUSG00000038507.6	243771	*Fbxl4*	-0.57	0.016	protein_coding	ENSMUSG00000040410.14	269514
Pacsin2	0.51	0.008	protein_coding	ENSMUSG00000016664.14	23970	*Tmem123*	-0.57	0.005	protein_coding	ENSMUSG00000050912.15	71929
Kri1	0.51	0.041	protein_coding	ENSMUSG00000035047.9	215194	*Hfe2*	-0.57	0.002	protein_coding	ENSMUSG00000038403.10	69585
Ptprd	0.51	0.037	protein_coding	ENSMUSG00000028399.18	19266	*Abi3bp*	-0.57	0.003	protein_coding	ENSMUSG00000035258.14	320712
Mdh1	0.50	0.007	protein_coding	ENSMUSG00000020321.15	17449	*Osbpl10*	-0.57	0.046	protein_coding	ENSMUSG00000040875.12	74486
Gm20712	0.50	0.016	sense_overlapping	ENSMUSG00000093677.1	NA	*Gnptab*	-0.56	0.001	protein_coding	ENSMUSG00000035311.16	432486
Cyb5r1	0.50	0.002	protein_coding	ENSMUSG00000026456.18	72017	*Gm15446*	-0.56	0.032	protein_coding	ENSMUSG00000090015.8	100642166
Ifitm3	0.50	0.037	protein_coding	ENSMUSG00000025492.6	66141	*Arl4a*	-0.56	0.003	protein_coding	ENSMUSG00000047446.18	11861
Pfkfb2	0.50	0.004	protein_coding	ENSMUSG00000026409.14	18640	*Bpgm*	-0.56	0.001	protein_coding	ENSMUSG00000038871.5	12183
						Slc16a9	-0.56	0.021	protein_coding	ENSMUSG00000037762.6	66859
						Inafm2	-0.56	0.019	antisense	ENSMUSG00000074918.4	100043272

bioRxiv preprint doi: https://doi.org/10.1101/2023.01.30.526183; this version posted January 31, 2023. The copyright holder for this preprint (which was not certified by peer review) is the author/funder, who has granted bioRxiv a license to display the preprint in perpetuity. It is made available under aCC-BY 4.0 International license.

Rassf1	-0.56	0.025	protein_coding	ENSMUSG00000010067.13	56289
Serinc3	-0.56	0.015	protein_coding	ENSMUSG00000017707.9	26943
Smad3	-0.56	0.008	protein_coding	ENSMUSG00000032402.12	17127
Lsp1	-0.55	0.014	protein_coding	ENSMUSG00000018819.10	16985
Lgals1	-0.55	0.025	protein_coding	ENSMUSG00000068220.5	16852
Abca6	-0.55	0.024	protein_coding	ENSMUSG00000044749.13	76184
Eif4ebp2	-0.55	0.003	protein_coding	ENSMUSG00000020091.14	13688
Cited4	-0.55	0.027	protein_coding	ENSMUSG00000070803.6	56222
Pip5k1a	-0.55	0.003	protein_coding	ENSMUSG00000028126.16	18720
Lpar1	-0.55	0.027	protein_coding	ENSMUSG00000038668.14	14745
Slc16a2	-0.55	0.028	protein_coding	ENSMUSG00000033965.10	20502
Zmiz2	-0.55	0.003	protein_coding	ENSMUSG00000041164.15	52915
Slc7a8	-0.55	0.034	protein_coding	ENSMUSG00000022180.6	50934
Amigo1	-0.55	0.002	protein_coding	ENSMUSG00000050947.9	229715
Mturn	-0.54	0.048	protein_coding	ENSMUSG00000038065.13	68235
Cd9	-0.54	0.024	protein_coding	ENSMUSG00000030342.8	12527
Klf13	-0.54	0.020	protein_coding	ENSMUSG00000052040.10	50794
Thbs2	-0.54	0.009	protein_coding	ENSMUSG00000023885.8	21826
Kif3c	-0.54	0.003	protein_coding	ENSMUSG00000020668.9	16570
Relt	-0.54	0.025	protein_coding	ENSMUSG00000008318.10	320100
Zfp9	-0.54	0.013	protein_coding	ENSMUSG00000072623.6	22750
RP23-478B10.1	-0.53	0.033	processed_pseudogene	ENSMUSG00000109600.1	NA
9030617O03Rik	-0.53	0.006	protein_coding	ENSMUSG00000021185.15	217830
Tnrc18	-0.53	0.040	protein_coding	ENSMUSG00000039477.16	231861
Stxbp6	-0.53	0.038	protein_coding	ENSMUSG00000046314.15	217517
Slc44a2	-0.53	0.002	protein_coding	ENSMUSG00000057193.7	68682
Ap1ar	-0.53	0.001	protein_coding	ENSMUSG00000074238.6	211556
Fzd7	-0.53	0.018	protein_coding	ENSMUSG00000041075.8	14369
Slc35f5	-0.53	0.020	protein_coding	ENSMUSG00000026342.10	74150
Qser1	-0.53	0.002	protein_coding	ENSMUSG00000074994.4	99003
Mfap5	-0.53	0.045	protein_coding	ENSMUSG00000030116.14	50530
Dlg1	-0.53	0.002	protein_coding	ENSMUSG00000022770.16	13383
Smyd2	-0.53	0.029	protein_coding	ENSMUSG00000026603.13	226830
Gm7236	-0.53	0.043	processed_pseudogene	ENSMUSG00000090389.1	NA
Dcn	-0.53	0.016	protein_coding	ENSMUSG00000019929.15	13179
Casq1	-0.53	0.028	protein_coding	ENSMUSG00000007122.11	12372
Ccl11	-0.52	0.006	protein_coding	ENSMUSG00000020676.2	20292
Gm15910	-0.52	0.016	processed_transcript	ENSMUSG00000087480.1	100504616
Peli2	-0.52	0.046	protein_coding	ENSMUSG00000021846.8	93834
Itpr1	-0.52	0.004	protein_coding	ENSMUSG00000030102.11	16438
St8sia5	-0.52	0.005	protein_coding	ENSMUSG00000025425.17	225742
4930453N24Rik	-0.52	0.001	protein_coding	ENSMUSG00000059920.9	67609
Aven	-0.52	0.038	protein_coding	ENSMUSG00000003604.14	74268
Hoxc10	-0.52	0.001	protein_coding	ENSMUSG00000022484.7	209448
Ccdc134	-0.52	0.015	protein_coding	ENSMUSG00000068114.6	76457
Klhl31	-0.51	0.002	protein_coding	ENSMUSG00000044938.8	244923
Vkorc1l1	-0.51	0.010	protein_coding	ENSMUSG00000066735.8	69568
Prkar2a	-0.51	0.001	protein_coding	ENSMUSG00000032601.13	19087
Sort1	-0.51	0.033	protein_coding	ENSMUSG00000068747.14	20661
Spast	-0.51	0.001	protein_coding	ENSMUSG00000024068.6	50850
Cdkn2aipnl	-0.51	0.019	protein_coding	ENSMUSG00000020392.8	52626
Ppp2cb	-0.51	0.005	protein_coding	ENSMUSG00000009630.10	19053
Lingo3	-0.51	0.029	protein_coding	ENSMUSG00000051067.7	237403
Tmod4	-0.51	0.007	protein_coding	ENSMUSG00000005628.12	50874
Itfg1	-0.51	0.009	protein_coding	ENSMUSG00000031703.8	71927
Polm	-0.51	0.044	protein_coding	ENSMUSG00000020474.11	54125
Dnase1	-0.51	0.047	protein_coding	ENSMUSG00000005980.15	13419
Fgd4	-0.51	0.013	protein_coding	ENSMUSG00000022788.16	224014
Rab10	-0.51	0.001	protein_coding	ENSMUSG00000020671.8	19325
Lpin1	-0.51	0.003	protein_coding	ENSMUSG00000020593.14	14245
Mb21d2	-0.51	0.025	protein_coding	ENSMUSG00000051065.8	239796
Jak1	-0.51	0.002	protein_coding	ENSMUSG00000028530.14	16451
Ergic1	-0.50	0.005	protein_coding	ENSMUSG00000001576.14	67458
Jph1	-0.50	0.009	protein_coding	ENSMUSG00000042686.5	57339
Bmpr1b	-0.50	0.044	protein_coding	ENSMUSG00000052430.15	12167
Ube2r2	-0.50	0.006	protein_coding	ENSMUSG00000036241.3	67615
Ghr	-0.50	0.002	protein_coding	ENSMUSG00000055737.12	14600

Table S11. Indirect calorimetry analysis in thermoneutral conditions (31°C) after two-week acclimatization period of male (55 weeks of age) Euploid and MAC21 mice fed a high-fat diet (18 weeks on diet).

	**Eu**	**MAC21**	***P***	**Eu**	**MAC21**	***P***	**Eu**	**MAC21**	***P***
**High-fat diet (Male)**	***Ad-libitum* (dark cycle)**			***Ad-libitum* (light cycle)**			***Ad-libitum* (24 hr)**		
N	5	4		5	4		5	4	
Body weight (g)							**58.1 ± 6.18**	**36.0 ± 7.39**	**0.0020**
Food intake (kcal)							7.67 ± 3.42	8.27 ± 3.87	0.814
VO2 (mL/kg lean mass/h)	**3185 ± 120**	**4182 ± 103**	**<0.001**	**2480 ± 103**	**3134 ± 144**	**0.007**	**2833 ± 103**	**3658 ± 112**	**<0.001**
VCO2 (mL/kg lean mass/h)	**2230 ± 91**	**3017 ± 150**	**0.002**	**1766 ± 70**	**2360 ± 221**	**0.025**	**1998 ± 76**	**2688 ± 183**	**0.007**
RER (VCO2/VO2)	0.70 ± 0.006	0.72 ± 0.024	0.342	0.71 ± 0.007	0.75 ± 0.033	0.245	0.70 ± 0.006	0.73 ± 0.028	0.282
EE (kcal/kg lean mass/h)	**14.9 ± 0.57**	**19.7 ± 0.55**	**<0.001**	**11.6 ± 0.48**	**14.9 ± 0.82**	**0.009**	**13.3 ± 0.48**	**17.3 ± 0.64**	**0.001**

Eu: Euploid; VO2: rate of oxygen consumption; VCO2: rate of carbon dioxide production; RER: respiratory exchange ratio; EE: energy expenditure

bioRxiv preprint doi: https://doi.org/10.1101/2023.01.30.526183; this version posted January 31, 2023. The copyright holder for this preprint (which was not certified by peer review) is the author/funder, who has granted bioRxiv a license to display the preprint in perpetuity. It is made available under aCC-BY 4.0 International license.

Table S12. With the exeption of *Myl4* , none of the genes are significantly upregulated above log2(FC) of 0.5

Atrophy-related genes

Gene Symbol	Log2(FC)	Adj.p-Value	Gene Type	GencodeID	EntrezID
Becn1	-0.243	0.0567	protein_coding	ENSMUSG00000035086.13	56208
Capn2	-0.018	0.9199	protein_coding	ENSMUSG00000026509.15	12334
Fbxo32	-0.465	0.0045	protein_coding	ENSMUSG00000022358.6	67731
Mustn1	0.461	0.1410	protein_coding	ENSMUSG00000042485.6	66175
Nedd4	-0.052	0.6234	protein_coding	ENSMUSG00000032216.14	17999
Psma6	0.100	0.5439	protein_coding	ENSMUSG00000021024.14	26443
Psmb2	0.196	0.2092	protein_coding	ENSMUSG00000028837.8	26445
Trim55	-0.265	0.0606	protein_coding	ENSMUSG00000060913.6	381485
Trim63	-0.315	0.0572	protein_coding	ENSMUSG00000028834.13	433766

Contractile and Structural genes

Gene Symbol	Log2(FC)	Adj.p-Value	Gene Type	GencodeID	EntrezID
Des	0.027	0.8863	protein_coding	ENSMUSG00000026208.9	13346
Dysf	-0.003	0.9892	protein_coding	ENSMUSG00000033788.15	26903
Mybpc1	0.344	0.2387	protein_coding	ENSMUSG00000020061.17	109272
Mybpc2	-0.152	0.3554	protein_coding	ENSMUSG00000038670.11	233199
Myh1	0.801	0.0879	protein_coding	ENSMUSG00000056328.14	17879
Myh2	-0.045	0.9449	protein_coding	ENSMUSG00000033196.17	17882
Myh3	-0.020	0.9831	protein_coding	ENSMUSG00000020908.14	17883
Myh7	0.420	0.7271	protein_coding	ENSMUSG00000053093.15	140781
Myh7b	0.340	0.7350	protein_coding	ENSMUSG00000074652.3	668940
Myh8	-0.155	0.7391	protein_coding	ENSMUSG00000055775.16	17885
Myl1	0.097	0.6449	protein_coding	ENSMUSG00000061816.15	17901
Myl3	-0.016	0.9928	protein_coding	ENSMUSG00000059741.13	17897
Myl4	**-1.214**	**0.0329**	**protein_coding**	**ENSMUSG00000061086.12**	**17896**
Mypn	0.079	0.4802	protein_coding	ENSMUSG00000020067.7	68802
Tnni2	-0.332	0.1263	protein_coding	ENSMUSG00000031097.15	21953

Fibrosis and Injury Repair related genes

Gene Symbol	Log2(FC)	Adj.p-Value	Gene Type	GencodeID	EntrezID
Col1a1	-0.410	0.1196	protein_coding	ENSMUSG00000001506.10	12842
Col1a2	-0.297	0.0738	protein_coding	ENSMUSG00000029661.16	12843
Col3a1	-0.075	0.8063	protein_coding	ENSMUSG00000026043.18	12825
Col4a1	0.217	0.6216	protein_coding	ENSMUSG00000031502.11	12826
Col4a2	0.114	0.7705	protein_coding	ENSMUSG00000031503.13	12827
Col4a3	0.080	0.7762	protein_coding	ENSMUSG00000079465.8	12828
Col4a3bp	-0.134	0.2699	protein_coding	ENSMUSG00000021669.14	68018
Col4a4	0.024	0.9291	protein_coding	ENSMUSG00000067158.9	12829
Col4a5	0.072	0.7762	protein_coding	ENSMUSG00000031274.16	12830
Col4a6	0.445	0.4745	protein_coding	ENSMUSG00000031273.16	94216
Col6a1	-0.201	0.3954	protein_coding	ENSMUSG00000001119.7	12833
Col6a2	-0.301	0.2191	protein_coding	ENSMUSG00000020241.13	12834
Col6a3	-0.036	0.8902	protein_coding	ENSMUSG00000048126.16	12835
Col6a6	0.099	0.6784	protein_coding	ENSMUSG00000043719.14	245026
Eln	-0.111	0.6890	protein_coding	ENSMUSG00000029675.12	13717
Mmp2	-0.230	0.2256	protein_coding	ENSMUSG00000031740.8	17390
Mmp14	-0.231	0.5334	protein_coding	ENSMUSG00000000957.9	17387
Scx	-0.239	0.5406	protein_coding	ENSMUSG00000034161.7	20289
Timp1	-0.528	0.5704	protein_coding	ENSMUSG00000001131.11	21857
Timp2	-0.467	0.0395	protein_coding	ENSMUSG00000017466.9	21858

Muscle Wasting related genes

Gene Symbol	Log2(FC)	Adj.p-Value	Gene Type	GencodeID	EntrezID
Aldh2	0.418	0.0280	protein_coding	ENSMUSG00000029455.14	11669
Apc	-0.152	0.2303	protein_coding	ENSMUSG00000005871.14	11789
Bnip3	0.176	0.3012	protein_coding	ENSMUSG00000078566.8	12176
Ccl2	0.284	0.8355	protein_coding	ENSMUSG00000035385.5	20296
Foxo1	0.101	0.4669	protein_coding	ENSMUSG00000044167.6	56458
Il6ra	-0.386	0.1657	protein_coding	ENSMUSG00000027947.11	16194
Il6st	0.018	0.9020	protein_coding	ENSMUSG00000021756.12	16195
Ly6c1	-0.160	0.6728	protein_coding	ENSMUSG00000079018.10	17067
Map1lc3a	-0.008	0.9780	protein_coding	ENSMUSG00000027602.9	66734
Ptprc	-0.091	0.8671	protein_coding	ENSMUSG00000026395.16	19264
Rela	-0.016	0.9408	protein_coding	ENSMUSG00000024927.7	19697
Xrcc5	0.135	0.5156	protein_coding	ENSMUSG00000026187.8	22596
Zmpste24	-0.036	0.8016	protein_coding	ENSMUSG00000043207.10	230709

bioRxiv preprint doi: https://doi.org/10.1101/2023.01.30.526183; this version posted January 31, 2023. The copyright holder for this preprint (which was not certified by peer review) is the author/funder, who has granted bioRxiv a license to display the preprint in perpetuity. It is made available under aCC-BY 4.0 International license.

Table S13. Tissue weights of male MAC21 and Euploid littermates (55 weeks of age) housed at thermoneutrality (31°C) and fed an HFD (18 weeks on diet).

	**Eu**	**MAC21**	***P***
N	5	4	
Body weight (g)	**60.3 ± 2.85**	**36.9 ± 3.71**	**0.001**
Gondal fat (gWAT, g)	1.62 ± 0.22	1.07 ± 0.05	0.065
Inguinal fat (iWAT, g)	**1.60 ± 0.11**	**0.58 ± 0.11**	**<0.001**
Liver (g)	**3.03 ± 0.33**	**1.66 ± 0.19**	**0.013**
BAT (g)	**0.25 ± 0.02**	**0.10 ± 0.02**	**<0.001**
Pancreas (g)	**0.44 ± 0.02**	**0.34 ± 0.03**	**0.015**

Eu: Euploid; gWAT: gondal white adipose tissue; iWAT: inguinal white adipose tissue; BAT: brown adipose tissue

bioRxiv preprint doi: https://doi.org/10.1101/2023.01.30.526183; this version posted January 31, 2023. The copyright holder for this preprint (which was not certified by peer review) is the author/funder, who has granted bioRxiv a license to display the preprint in perpetuity. It is made available under aCC-BY 4.0 International license.

Table S14 Skeletal muscle (HFD, 31°C)

Human genes expressed

Gene Symbol	Log(Fold Change)	Adj.p-Value	Gene Type	GencodeID	EntrezID
SH3BGR	12.10	1.374E-05	protein_coding	ENSG00000185437.13	6450
ATP5J	11.77	1.074E-05	protein_coding	ENSG00000154723.12	522
AGPAT3	11.06	1.809E-05	protein_coding	ENSG00000160216.18	56894
MAP3K7CL	10.85	1.826E-03	protein_coding	ENSG00000156265.15	56911
NDUFV3	10.82	1.506E-05	protein_coding	ENSG00000160194.17	4731
USP16	10.60	1.809E-05	protein_coding	ENSG00000156256.14	10600
ATP5O	10.56	1.374E-05	protein_coding	ENSG00000241837.6	539
SON	10.22	4.835E-05	protein_coding	ENSG00000159140.18	6651
DYRK1A	9.79	5.371E-05	protein_coding	ENSG00000157540.19	1859
SOD1	9.73	5.317E-05	protein_coding	ENSG00000142168.14	6647
HLCS	9.69	2.292E-05	protein_coding	ENSG00000159267.14	3141
DIP2A	9.60	2.003E-05	protein_coding	ENSG00000160305.17	23181
SUMO3	9.59	7.614E-05	protein_coding	ENSG00000184900.15	6612
DSCR3	9.48	2.143E-05	protein_coding	ENSG00000157538.13	10311
PIGP	9.48	2.003E-05	protein_coding	ENSG00000185808.13	51227
GABPA	9.38	2.487E-05	protein_coding	ENSG00000154727.10	2551
POFUT2	9.30	2.292E-05	protein_coding	ENSG00000186866.16	23275
ETS2	9.28	2.292E-05	protein_coding	ENSG00000157557.11	2114
IFNAR1	9.23	4.923E-05	protein_coding	ENSG00000142166.12	3454
UBE2G2	9.22	3.893E-04	protein_coding	ENSG00000184787.18	7327
PTTG1IP	9.13	2.331E-04	protein_coding	ENSG00000183255.11	754
CCT8	9.13	7.614E-05	protein_coding	ENSG00000156261.12	10694
BRWD1	9.03	1.774E-04	protein_coding	ENSG00000185658.13	54014
N6AMT1	9.03	2.421E-05	protein_coding	ENSG00000156239.11	29104
TRAPPC10	8.98	2.767E-05	protein_coding	ENSG00000160218.12	7109
IFNGR2	8.94	2.292E-05	protein_coding	ENSG00000159128.14	3460
MCM3AP	8.89	1.277E-04	protein_coding	ENSG00000160294.10	8888
SLC37A1	8.73	3.382E-05	protein_coding	ENSG00000160190.13	54020
CLIC6	8.71	4.700E-05	protein_coding	ENSG00000159212.12	54102
RRP1B	8.68	5.371E-05	protein_coding	ENSG00000160208.12	23076
BACH1	8.54	3.647E-05	protein_coding	ENSG00000156273.15	571
GART	8.46	3.576E-04	protein_coding	ENSG00000159131.16	2618
URB1	8.43	2.435E-04	protein_coding	ENSG00000142207.6	9875
RWDD2B	8.42	4.700E-05	protein_coding	ENSG00000156253.6	10069
MX2	8.37	4.835E-05	protein_coding	ENSG00000183486.12	4600
WRB	8.30	1.779E-04	protein_coding	ENSG00000182093.14	7485
PKNOX1	8.30	7.295E-05	protein_coding	ENSG00000160199.14	5316
ITSN1	8.29	4.700E-05	protein_coding	ENSG00000205726.14	6453
CYYR1	8.28	4.700E-05	protein_coding	ENSG00000166265.11	116159
C21orf33	8.28	9.855E-05	protein_coding	ENSG00000160221.16	8209
MX1	8.25	1.003E-04	protein_coding	ENSG00000157601.13	4599
CSTB	8.23	4.416E-04	protein_coding	ENSG00000160213.5	1476
SLC5A3	8.22	4.700E-05	protein_coding	ENSG00000198743.6	6526
CH507-9B2.3	8.14	1.161E-04	protein_coding	ENSG00000280071.3	102724023
SYNJ1	8.03	4.835E-05	protein_coding	ENSG00000159082.17	8867
PDXK	8.01	8.893E-04	protein_coding	ENSG00000160209.18	8566
APP	7.98	1.770E-03	protein_coding	ENSG00000142192.20	351
HMGN1	7.91	8.444E-04	protein_coding	ENSG00000205581.10	3150
PCNT	7.84	3.454E-04	protein_coding	ENSG00000160299.16	5116
ADAMTS1	7.82	6.390E-05	protein_coding	ENSG00000154734.14	9510
MRPL39	7.81	1.278E-04	protein_coding	ENSG00000154719.13	54148
PSMG1	7.81	1.938E-04	protein_coding	ENSG00000183527.11	8624
SETD4	7.80	1.003E-04	protein_coding	ENSG00000185917.13	54093
CBR1	7.79	1.693E-04	protein_coding	ENSG00000159228.12	873
URB1-AS1	7.77	7.722E-05	lincRNA	ENSG00000256073.3	84996
TMEM50B	7.75	1.265E-03	protein_coding	ENSG00000142188.16	757
CRYZL1	7.68	3.101E-04	protein_coding	ENSG00000205758.11	9946
TTC3	7.67	2.025E-03	protein_coding	ENSG00000182670.13	7267
RRP1	7.58	1.277E-04	protein_coding	ENSG00000160214.12	8568
PRMT2	7.53	5.059E-04	protein_coding	ENSG00000160310.16	3275
RUNX1	7.49	1.934E-04	protein_coding	ENSG00000159216.18	861
TSPEAR-AS1	7.49	2.963E-04	antisense	ENSG00000235890.2	NA
LINC00649	7.47	7.202E-04	antisense	ENSG00000237945.7	100506334
FAM207A	7.46	1.672E-04	protein_coding	ENSG00000160256.12	85395
PAXBP1	7.44	1.431E-03	protein_coding	ENSG00000159086.14	94104
MRPS6	7.39	1.938E-04	protein_coding	ENSG00000243927.5	64968
BACE2	7.36	1.161E-04	protein_coding	ENSG00000182240.15	25825
IFNAR2	7.36	1.505E-04	protein_coding	ENSG00000159110.19	3455
PRDM15	7.34	1.277E-04	protein_coding	ENSG00000141956.13	63977
JAM2	7.29	3.163E-04	protein_coding	ENSG00000154721.14	58494
CH507-9B2.5	7.28	1.743E-04	protein_coding	ENSG00000275464.4	102724159
C21orf59	7.25	1.476E-04	protein_coding	ENSG00000159079.18	56683
ADARB1	7.20	1.804E-04	protein_coding	ENSG00000197381.15	104
S100B	7.20	1.968E-04	protein_coding	ENSG00000160307.9	6285
BTG3	7.16	1.824E-02	protein_coding	ENSG00000154640.14	10950
SCAF4	7.15	2.025E-03	protein_coding	ENSG00000156304.14	57466
TSPEAR-AS2	7.12	1.733E-04	antisense	ENSG00000182912.6	NA
LTN1	7.09	1.733E-04	protein_coding	ENSG00000198862.13	26046
DNAJC28	6.87	3.618E-04	protein_coding	ENSG00000177692.11	54943
HSPA13	6.78	6.158E-04	protein_coding	ENSG00000155304.5	6782
C2CD2	6.71	1.520E-03	protein_coding	ENSG00000157617.16	25966
CHAF1B	6.61	5.064E-04	protein_coding	ENSG00000159259.7	8208
DONSON	6.56	7.755E-04	protein_coding	ENSG00000159147.17	29980
ITGB2	6.42	1.265E-03	protein_coding	ENSG00000160255.17	3689
ZBTB21	6.27	8.513E-03	protein_coding	ENSG00000173276.13	49854
SIM2	6.24	7.158E-04	protein_coding	ENSG00000159263.15	6493
WDR4	6.21	2.038E-03	protein_coding	ENSG00000160193.11	10785
LINC00310	6.13	1.236E-03	lincRNA	ENSG00000227456.7	114036
UMODL1	6.10	2.512E-03	protein_coding	ENSG00000177398.18	89766
PFKL	6.09	9.476E-03	protein_coding	ENSG00000141959.16	5211
TIAM1	6.09	1.250E-03	protein_coding	ENSG00000156299.13	7074
IL10RB	6.04	1.250E-03	protein_coding	ENSG00000243646.9	3588
C21orf58	5.95	1.250E-03	protein_coding	ENSG00000160298.17	54058
RCAN1	5.93	2.088E-03	protein_coding	ENSG00000159200.17	1827
DOPEY2	5.91	4.912E-03	protein_coding	ENSG00000142197.12	9980
EVA1C	5.77	2.323E-03	protein_coding	ENSG00000166979.12	59271
CBSL	5.66	1.484E-02	protein_coding	ENSG00000274276.4	102724560
U2AF1	5.60	3.793E-03	protein_coding	ENSG00000160201.11	7307
LCA5L	5.36	6.233E-03	protein_coding	ENSG00000157578.13	150082
LINC00205	5.14	1.198E-02	lincRNA	ENSG00000223768.1	NA
BX322557.10	5.14	1.428E-02	processed_transcript	ENSG00000215447.7	NA
KCNE2	5.12	1.180E-02	protein_coding	ENSG00000159197.3	9992
TSPEAR	5.06	3.148E-02	protein_coding	ENSG00000175894.14	54084
CBS	5.03	2.947E-02	protein_coding	ENSG00000160200.17	875
MORC3	5.02	4.033E-02	protein_coding	ENSG00000159256.12	23515
DSCR4	4.96	1.823E-02	protein_coding	ENSG00000184029.9	10281
PAXBP1-AS1	4.92	1.428E-02	antisense	ENSG00000238197.5	100506215
LINC01436	4.77	3.027E-02	lincRNA	ENSG00000231106.2	NA
RSPH1	4.77	3.187E-02	protein_coding	ENSG00000160188.9	89765
YBEY	4.46	4.033E-02	protein_coding	ENSG00000182362.13	54059

Mouse up-regulated genes

Gene Symbol	Log(Fold Change)	Adj.p-Value	Gene Type	GencodeID	EntrezID
Eif3s6-ps2	5.04	0.010	processed_pseudogene	ENSMUSG00000091697.1	NA
Gm13534	4.81	0.029	processed_pseudogene	ENSMUSG00000053472.3	NA
RP23-162M13.9	4.76	0.021	processed_pseudogene	ENSMUSG00000110569.1	NA
Dmp1	4.74	0.019	protein_coding	ENSMUSG00000029307.7	13406
Myo1a	4.71	0.040	protein_coding	ENSMUSG00000025401.8	432516
Fut1	4.39	0.040	protein_coding	ENSMUSG00000008461.6	14343
Ccdc92	3.77	0.047	protein_coding	ENSMUSG00000037979.13	215707
Mthfd2	3.70	0.003	protein_coding	ENSMUSG00000005667.8	17768
Slc7a5	3.58	0.002	protein_coding	ENSMUSG00000040010.10	20539
Asns	3.30	0.007	protein_coding	ENSMUSG00000029752.12	27053
Aldh18a1	2.60	0.002	protein_coding	ENSMUSG00000025007.13	56454
E2f2	2.35	0.004	protein_coding	ENSMUSG00000018983.9	242705
A930003A15Rik	2.30	0.029	protein_coding	ENSMUSG00000075330.3	NA
Cpne2	2.19	0.001	protein_coding	ENSMUSG00000034361.10	234577
Ankrd1	2.01	0.001	protein_coding	ENSMUSG00000024803.8	107765
Aldh1l2	1.83	0.045	protein_coding	ENSMUSG00000020256.14	216188
Atf5	1.82	0.001	protein_coding	ENSMUSG00000038539.15	107503
Tceal7	1.81	0.002	protein_coding	ENSMUSG00000079428.8	100040972
Spag5	1.71	0.018	protein_coding	ENSMUSG00000002055.9	54141
Itga9	1.66	0.003	protein_coding	ENSMUSG00000039115.13	104099
Tnfaip2	1.55	0.001	protein_coding	ENSMUSG00000021281.15	21928
Cdkn1a	1.52	0.001	protein_coding	ENSMUSG00000023067.13	12575
Slc7a1	1.49	0.019	protein_coding	ENSMUSG00000041313.14	11987
Sesn2	1.49	0.041	protein_coding	ENSMUSG00000028893.8	NA
Lrp2bp	1.45	0.001	protein_coding	ENSMUSG00000031637.13	67620
Abra	1.35	0.029	protein_coding	ENSMUSG00000042895.5	NA
Otud1	1.33	0.046	protein_coding	ENSMUSG00000043415.5	71198
A930018M24Rik	1.32	0.021	protein_coding	ENSMUSG00000091089.1	NA
Ctnna3	1.32	0.018	protein_coding	ENSMUSG00000060843.11	216033
A830010M20Rik	1.31	0.011	protein_coding	ENSMUSG00000044060.18	NA
Ift122	1.30	0.001	protein_coding	ENSMUSG00000030323.13	81896
6430548M08Rik	1.27	0.024	protein_coding	ENSMUSG00000031824.15	234797
Gadd45a	1.26	0.025	protein_coding	ENSMUSG00000036390.8	13197
Slc47a1	1.24	0.027	protein_coding	ENSMUSG00000010122.14	67473
Zfp870	1.23	0.018	protein_coding	ENSMUSG00000095325.1	240066
Clu	1.16	0.000	protein_coding	ENSMUSG00000022037.14	12759
Nnt	1.16	0.001	protein_coding	ENSMUSG00000025453.16	18115
Angpt1	1.09	0.007	protein_coding	ENSMUSG00000022309.8	11600
Tmem47	1.09	0.049	protein_coding	ENSMUSG00000025666.16	192216
Camta1	1.06	0.039	protein_coding	ENSMUSG00000014592.19	100072
Rcan1	1.05	0.013	protein_coding	ENSMUSG00000022951.15	54720
Iars	1.03	0.029	protein_coding	ENSMUSG00000037851.13	105148
Klf10	0.97	0.025	protein_coding	ENSMUSG00000037465.9	21847
Cars	0.93	0.017	protein_coding	ENSMUSG00000010755.17	27267
Rb1	0.93	0.029	protein_coding	ENSMUSG00000022105.6	19645
Pacsin2	0.92	0.024	protein_coding	ENSMUSG00000016664.14	23970
Extl1	0.87	0.021	protein_coding	ENSMUSG00000028838.11	56219
Clic4	0.85	0.020	protein_coding	ENSMUSG00000037242.8	29876
Cebpg	0.84	0.025	protein_coding	ENSMUSG00000056216.9	12611
Fads6	0.80	0.031	protein_coding	ENSMUSG00000044788.10	NA
Prss23	0.79	0.026	protein_coding	ENSMUSG00000039405.7	76453
Gars	0.76	0.040	protein_coding	ENSMUSG00000029777.10	353172
Lmod3	0.74	0.008	protein_coding	ENSMUSG00000044086.8	320502
Sars	0.73	0.031	protein_coding	ENSMUSG00000068739.13	20226
Palld	0.66	0.017	protein_coding	ENSMUSG00000058056.16	72333
Yars	0.65	0.037	protein_coding	ENSMUSG00000028811.11	NA
Tob1	0.62	0.040	protein_coding	ENSMUSG00000037573.5	22057
Nars	0.60	0.035	protein_coding	ENSMUSG00000024587.9	70223
Dmpk	0.57	0.023	protein_coding	ENSMUSG00000030409.15	13400

Mouse down-regulated genes

Gene Symbol	Log(Fold Change)	Adj.p-Value	Gene Type	GencodeID	EntrezID
Cdh4	-3.28	0.008	protein_coding	ENSMUSG00000000305.12	12561
Shc2	-2.79	0.034	protein_coding	ENSMUSG00000020312.11	216148
Nhs	-2.73	0.021	protein_coding	ENSMUSG00000059493.13	195727
Cntnap2	-2.42	0.020	protein_coding	ENSMUSG00000039419.17	66797
Gdf11	-2.34	0.000	protein_coding	ENSMUSG00000025352.6	NA
Pla2g7	-2.20	0.003	protein_coding	ENSMUSG00000023913.17	27226
Aurka	-2.11	0.029	protein_coding	ENSMUSG00000027496.15	20878
Ctxn3	-1.90	0.001	protein_coding	ENSMUSG00000069372.3	629147
Mss51	-1.80	0.001	protein_coding	ENSMUSG00000021815.3	74843
Col24a1	-1.77	0.023	protein_coding	ENSMUSG00000028197.4	71355
Mib1	-1.69	0.002	protein_coding	ENSMUSG00000024294.13	225164
Ces1d	-1.67	0.047	protein_coding	ENSMUSG00000056973.6	104158
Aebp1	-1.60	0.000	protein_coding	ENSMUSG00000020473.13	11568
Gm45123	-1.56	0.027	lincRNA	ENSMUSG00000108322.1	71368
Nos1	-1.48	0.046	protein_coding	ENSMUSG00000029361.18	18125
Wbscr17	-1.48	0.018	protein_coding	ENSMUSG00000034040.16	212996
Col22a1	-1.46	0.003	protein_coding	ENSMUSG00000079022.9	69700
Sorbs2	-1.43	0.002	protein_coding	ENSMUSG00000031626.17	234214
2310065F04Rik	-1.39	0.029	lincRNA	ENSMUSG00000087410.7	74184
Enox2	-1.35	0.013	protein_coding	ENSMUSG00000031109.16	209224
Tpbgl	-1.35	0.022	protein_coding	ENSMUSG00000096606.2	100503386
mt-Te	-1.29	0.035	Mt_tRNA	ENSMUSG00000064369.1	NA
Mettl11b	-1.26	0.027	protein_coding	ENSMUSG00000040113.14	240879
Mstn	-1.25	0.001	protein_coding	ENSMUSG00000026100.6	17700
Rasd2	-1.24	0.001	protein_coding	ENSMUSG00000034472.13	75141
Cpeb1	-1.20	0.005	protein_coding	ENSMUSG00000025586.17	12877
Clec3b	-1.19	0.014	protein_coding	ENSMUSG00000025784.4	21922
Gpx3	-1.19	0.035	protein_coding	ENSMUSG00000018339.11	14778
Pmepa1	-1.12	0.029	protein_coding	ENSMUSG00000038400.15	65112
Smox	-1.11	0.026	protein_coding	ENSMUSG00000027333.18	228608
Ttll7	-1.10	0.004	protein_coding	ENSMUSG00000036745.15	70892
Calm3	-1.09	0.001	protein_coding	ENSMUSG00000019370.10	12315
Myoc	-1.09	0.016	protein_coding	ENSMUSG00000026697.10	17926
Plekho1	-1.09	0.021	protein_coding	ENSMUSG00000015745.9	67220
Slc16a3	-1.09	0.010	protein_coding	ENSMUSG00000025161.16	80879
Me1	-1.05	0.001	protein_coding	ENSMUSG00000032418.15	17436
Ppp1r14b	-1.01	0.009	protein_coding	ENSMUSG00000056612.6	18938
Vasp	-0.98	0.029	protein_coding	ENSMUSG00000030403.9	22323
Plekhb1	-0.96	0.023	protein_coding	ENSMUSG00000030701.17	27276
Zfp467	-0.92	0.018	protein_coding	ENSMUSG00000068551.12	68910
Net1	-0.90	0.009	protein_coding	ENSMUSG00000021215.14	56349
Sms	-0.88	0.002	protein_coding	ENSMUSG00000071708.11	20603
Ccnk	-0.88	0.013	protein_coding	ENSMUSG00000021258.9	12454
Lgals1	-0.82	0.021	protein_coding	ENSMUSG00000068220.5	16852
Mylk2	-0.81	0.002	protein_coding	ENSMUSG00000027470.9	228785
Calm1	-0.81	0.018	protein_coding	ENSMUSG00000001175.13	12313
Rxrg	-0.78	0.032	protein_coding	ENSMUSG00000015843.10	20183
Drap1	-0.75	0.022	protein_coding	ENSMUSG00000024914.16	66556
Ankrd9	-0.74	0.010	protein_coding	ENSMUSG00000037904.14	74251
Kmt5a	-0.63	0.031	protein_coding	ENSMUSG00000049327.17	67956
Ccdc50	-0.61	0.040	protein_coding	ENSMUSG00000038127.14	67501
Ttc7b	-0.57	0.050	protein_coding	ENSMUSG00000033530.7	104718
Cdc34	-0.55	0.047	protein_coding	ENSMUSG00000020307.14	216150
Csde1	-0.53	0.034	protein_coding	ENSMUSG00000068823.10	229663

bioRxiv preprint doi: https://doi.org/10.1101/2023.01.30.526183; this version posted January 31, 2023. The copyright holder for this preprint (which was not certified by peer review) is the author/funder, who has granted bioRxiv a license to display the preprint in perpetuity. It is made available under aCC-BY 4.0 International license.

Table S15. List of primers used for qPCR analysis of gene expression.

Gene Symbol	Gene Name	Forward Sequence	Reverse Sequence
Acadm, Mcad	Acyl-Coenzyme A dehydrogenase, medium chain	AGGGTTTAGTTTTGAGTTGACGG	CCCCGCTTTTGTCATATTCCG
Acc	Acetyl-CoA carboxylase	TGACAGACTGATCGCAGAGAAAG	TGGAGAGCCCCACACACA
Acox1	Acyl-Coenzyme A oxidase 1, palmitoyl	AGATTGGTAGAAATTGCTGCAAA	ACGCCACTTCCTTGCTCTTC
Adgre1, F4/80	Adhesion G protein-coupled receptor E1	CCCCAGTGTCCTTACAGAGTG	GTGCCCAGAGTGGATGTCT
Arg1	Arginase 1	CTCCAAGCCAAAGTCCTTAGAG	AGGAGCTGTCATTAGGGACATC
Atp2a1	ATPase, Ca2+ transporting, fast twitch 1, Serca1	TGTTTGTCCTATTTCGGGGTG	AATCCGCACAAGCAGGTCTTC
Atp2a2	ATPase C2+ transporting slow twitch 2, Serca2a	GAGAACGCTCACACAAAGACC	CAATTCGTTGGAGCCCCAT
Atp5a1	ATP synthase, H+ transporting, mitochondrial F1 complex, alpha subunit 1	TCTCCATGCCTCTAACACTCG	CCAGGTCAACAGACGTGTCAG
Calm2	Calmodulin 2	ACGGGGATGGGACAATAACAA	CACACGGAATGCTTCTCTAATCT
Ccl3	Chemokine (C-C motif) ligand 3	TTCTCTGTACCATGACACTCTGC	CGTGGAATCTTCCGGCTGTAG
Ccl4	Chemokine (C-C motif) ligand 4	TTCCTGCTGTTTCTCTTACACCT	CTGTCTGCCTCTTTTGGTCAG
Ccr7	Chemokine (C-C motif) receptor 7	TGTACGAGTCGGTGTGCTTC	GGTAGGTATCCGTCATGGTCTTG
CD206	Mannose receptor, C type 1	CTCTGTTCAGCTATTGGACGC	CGGAATTTCTGGGATTCAGCTTC
CD36	CD36 molecule	ATGGGCTGTGATCGGAACTG	AGCCAGGACTGCACCAATAAC
CD68	CD68 antigen	TTCTGCTGTGGAAATGCAAG	CAATGATGAGAGGCAGCAAG
Cebpa	CCAAT/enhancer binding protein alpha	CAAGAACAGCAACGAGTACCG	GTCACTGGTCAACTCCAGCAC
Cidea	Cell death-inducing DNA fragmentation factor, alpha subunit-like effector A	TGACATTCATGGGATTGCAGAC	GGCCAGTTGTGATGACTAAGAC
Cidec	Cell death-inducing DFFA-like effector c	ATGGACTACGCCATGAAGTCT	CGGTGCTAACACGACAGGG
Ckb	Brain/BAT isoform fo CK	AGTTCCCTGATCTGAGCAGC	GAATGGCGTCGTCCAAAGTAA
Ckm	Creatine kinase, muscle	CTGACCCCTGACCTCTACAAT	CATGGCGGTCCTGGATGAT
Col1a1	Collagen, type I, alpha 1	GCTCCTCTTAGGGGCCACT	CCACGTCTCACCATTGGGG
Col3a1	Collagen, type III, alpha 1	GGGTTTCCCTGGTCCTAAAG	CCTGGTTTCCCATTTTCTCC
Col6a1	Collagen, type VI, alpha 1	GATGAGGGTGAAGTGGGAGA	CAGCACGAAGAGGATGTCAA
Cox8b	Cytochrome c oxidase subunit 8B	TGTGGGGATCTCAGCCATAGT	AGTGGGCTAAGACCCATCCTG
Cpt1a	Carnitine palmitoyltransferase 1a, liver	CACCAACGGGCTCATCTTCTA	CAAAATGACCTAGCCTTCTATCGAA
Cpt1b	Carnitine palmitoyltransferase 1b, muscle	GGTCCCATAAGAAACAAGACCTCC	CAGAAAGTACCTCAGCCAGGAAAG
Dgat1	Diacylglycerol O-acyltransferase 1	GCCTTACTGGTTGAGTCTATCAC	GCACCACAGGTTGACATCC
Dgat2	Diacylglycerol O-acyltransferase 2	GCGCTACTTCCGAGACTACTT	GGGCCTTATGCCAGGAAACT
Dio2	Deiodinase, iodothyronine, type II	AATTATGCCTCGGAGAAGACCG	GGCAGTTGCCTAGTGAAAGGT
Dio3	Deiodinase, iodothyronine type III	CACGGCCTTCATGCTCTGG	CGGTTGTCGTCTGATACGCA
F4/80	Adhesion G protein-coupled receptor E1	CCCCAGTGTCCTTACAGAGTG	GTGCCCAGAGTGGATGTCT
Fasn	Fatty acid synthase	GGAGGTGGTGATAGCCGGTAT	TGGGTAATCCATAGAGCCCAG
Gatm	Glycine amidinotransferase (L-arginine:Gglycine amidotransferase), mitochondrial protein nuclear encoded	GCTTCCTCCCGAAATTCCTGT	CCTCTAAAGGGTCCCATTCGT
Gpat1, Gpam	Glycerol-3-phosphate acyltransferase, mitochondrial	CATCCTCTTTTGCCACAACAT	ACAGAATGTCTTTGCGTCCA
Gpat2	Glycerol-3-phosphate acyltransferase 2, mitochondrial	CACTGCTCCAGGTTTTGATG	AGGTTGGCAGCAATTCCATAC
Gpat4	Glycerol-3-phosphate acyltransferase 4	AGCTTGATTGTCAACCTCCTG	CCGTTGGTGTAGGGCTTGT
Hnf4a	Hepatic nuclear factor 4, alpha	CACGCGGAGGTCAAGCTAC	CCCAGAGATGGGAGAGGTGAT
Il-10	Interleukin 10	TAGTCCTTCCTACCCCAATTTCC	TTGGTCCTTAGCCACTCCTTC
Il-1β	Interleukin 1 beta	GCTCTTACTGACTGGCATGAG	CGCAGCTCTAGGAGCATGTG
IL6	Interleukin 6	GCCACCTTTTGACAGTGATGAG	GACAGCCCAGGTCAAAGGTT
Lcad, Acadl	Acyl-Coenzyme A dehydrogenase, long-chain	TCTTTTCCTCGGAGCATGACA	GACCTCTCTACTCACTTCTCCAG
Lipe	Lipase, hormone sensitive	CCAGCCTGAGGGCTTACTG	CTCCATTGACTGTGACATCTCG
Mb	Myoglobin	CTGTTTAAGACTCACCCTGAGAC	GGTGCAACCATGCTTCTTCA
Mcad	Acyl-Coenzyme A dehydrogenase, medium chain	AGGGTTTAGTTTTGAGTTGACGG	CCCCGCTTTTGTCATATTCCG
Mcp1	chemokine (C-C motif) ligand 2	TTAAAAACCTGGATCGGAACCAA	GCATTAGCTTCAGATTTACGGGT
Mct10	Slc16a10, olute carrier family 16, member 10; Tat1	GAGGTGGAGCTGACGAGGT	CATGGACACGAAGAGCACCC
Mct8	Slc16a2, monocarboxylic acid transporter, member 2, Xpct	CGGCTGGATAGTGGTGTTTG	CAGAGTTATGGATGCCGAAGATG
Mgl2	Macrophage galactose N-acetyl-galactosamine specific lectin 2	GCATGAAGGCAGCTGCTATTGGTT	TAGGCCCATCCAGCTAAGCACATT
Mlxipl, ChREBP	MLX interacting protein-like	AGATGGAGAACCGACGTATCA	ACTGAGCGTGCTGACAAGTC
mt-Co2	Mitochondrial cytochrome c oxidase II	GCCGACTAAATCAAGCAACA	CAATGGGCATAAAGCTATGG
mt-Cytb	Mitochondrially encoded cytochrome b	CATTTATTATCGCGGCCCTA	TGTTGGGTTGTTTGATCCTG
myh1	Myosin heavy chain 2x, myosin 1	GCGAATCGAGGCTCAGAACAA	GTAGTTCCGCCTTCGGTCTTG
myh2	Myosin heavy chain 2a, myosin 2	AAGTGACTGTGAAAACAGAAGCA	GCAGCCATTTGTAAGGGTTGAC
Myh4, MyhII2b	Myosin, heavy polypeptide 4, skeletal muscle	CTTTGCTTACGTCAGTCAAGGT	AGCGCCTGTGAGCTTGTAAA
Myh7	Myosin hevy chain polypeptide 7	ACTGTCAACACTAAGAGGGTCA	TTGGATGATTTGATCTTCCAGGG
Myl2	Myosin light poplypeptide 2	ATCGACAAGAATGACCTAAGGGA	ATTTTTCACGTTCACTCGTCCT
Myod1	Myogenic differentiation 1	CCACTCCGGGACATAGACTTG	AAAAGCGCAGGTCTGGTGAG
Myog	Myogenin	GAGACATCCCCCTATTTCTACCA	GCTCAGTCCGCTCATAGCC
Ndufa1	NADH:ubiquinone oxidoreductase subunit A1	ATGTGGTTCGAGATTCTCCCT	TGGTACTGAACACGAGCAACT
Nos2	Nitric oxide synthase 2, inducible	GTTCTCAGCCCAACAATACAAGA	GTGGACGGGTCGATGTCAC
Nr1h4, Fxr	Nuclear receptor subfamily 1, group H, member 4	GCTTGATGTGCTACAAAAGCTG	CGTGGTGATGGTTGAATGTCC
P2rx5	Purinergic receptor P2X, ligand-gated ion channel, 5	TGGAAGGGGTTCGTGTTGTC	AGGGAAGTGTCAATGTCCTGA
Pln	Phospholamban	CTCGCTCGGCTATCAGGAGAG	AGCATCACAATGATGCAGATCAG
Pnpla2, Atgl	Patatin-like phospholipase domain containing 2	TGTGGCCTCATTCCTCCTAC	TCGTGGATGTTGGTGGAGCT
Ppara	Peroxisome proliferator activated receptor alpha	CTATAATTTGCTGTGGAGATCGGC	GGATGGTTGCTCTGCAGGT
Ppard	Peroxisome proliferator activated receptor delta	TCCATCGTCAACAAAGACGGG	ACTTGGGCTCAATGATGTCAC
Ppargc1a, Pgc1a	Peroxisome proliferative activated receptor, gamma, coactivator 1 alpha	TATGGAGTGACATAGAGTGTGCT	CCACTTCAATCCACCCAGAAAG
Ppargc1b, Pgc1b	Peroxisome proliferative activated receptor, gamma, coactivator 1 beta	TCCTGTAAAAGCCCGGAGTAT	GCTCTGGTAGGGGCAGTGA
Pparγ	Peroxisome proliferator activated receptor gamma	TCGCTGATGCACTGCCTATG	GAGAGGTCCACAGAGCTGATT
Prdm16	PR domain containing 16	CAGCACGGTGAAGCCATTC	GCGTGCATCCGCTTGTG
Ptgs2, Cox2 (COXII)	Prostaglandin-endoperoxide synthase 2	GCCGACTAAATCAAGCAACA	CAATGGGCATAAAGCTATGG
Retnla	Resistin like alpha	CCAATCCAGCTAACTATCCCTCC	ACCCAGTAGCAGTCATCCCA
Rplp0, 36B4	Ribosomal protein, large, P0	AGATTCGGGATATGCTGTTGGC	TCGGGTCCTAGACCAGTGTTC
Scd1	Stearoyl-Coenzyme A desaturase 1	TTCTTGCGATACACTCTGGTGC	CGGGATTGAATGTTCTTGTCGT
Sirt1	Sirtuin 1	GCTGACGACTTCGACGACG	TCGGTCAACAGGAGGTTGTCT
Slc6a8	Solute carrier family 6 (neurotransmitter transporter, creatine), member 8	GCAGGGTGTGCATATCTCCAA	TACCCCCACTCACATCAGTCA
Sln	Sarcolipin	TGTGCCCCTGCTCCTCTTC	TGATTGCACACCAAGGCTTG
Srebf1, Srebp1c	Sterol regulatory element binding transcription factor 1	GGAGCCATGGATTGCACATT	GGCCCGGGAAGTCACTGT
Srebf2	Sterol regulatory element binding factor 2	GCGTTCTGGAGACCATGGA	ACAAAGTTGCTCTGAAAACAAATCA
Tbp	TATA-box binding protein	TGGTGTGCACAGGAGCCAAG	TTCACATCACAGCTCCCCAC
Thra1	Thyroid hormone receptor alpha, nr1a1	CTGACCTCCGCATGATCGG	GGTGGGGCACTCGACTTTC
Thrb	Thyroid hormone receptor beta	CCAGAGGTACACGAAGTGTGC	AGGTTTCCAGGGTAACTACAGG
Tnfa	tumor necrosis factor alpha	ATGCTGGGACAGTGACCTGG	CCTTGATGGTGGTGCATGAG
Tnnc1	Troponin C	GCGGTAGAACAGTTGACAGAG	CCAGCTCCTTGGTGCTGAT
Tnni1	Troponin I, skeletal, slow 1	ATGCCGGAAGTTGAGAGGAAA	TCCGAGAGGTAACGCACCTT
Tnnt1	Troponin T1	CCTGTGGTGCCTCCTTTGATT	TGCGGTCTTTTAGTGCAATGAG
Tnnt2	Troponin T2	GAGCTACAGACTCTGATCGAGG	CCGCTCATTGCGAATACGC
Tpm3	Tropomyosin 3, gamma	ACCACCATCGAGGCGGTAA	CCCTTTCCTCCGCATCATCA
Ucp1	Uncoupling protein 1 (mitochondrial, proton carrier)	AGGCTTCCAGTACCATTAGGT	CTGAGTGAGGCAAAGCTGATTT
Ucp3	Uncoupling protein 3 (mitochondrial, proton carrier)	CCGATTTCAAGCCATGATACGC	GGCATCCATAGTCCCTCTGTATT
